# PDE1B and PDE10A as novel targets for schizophrenia: from molecular design and synthesis to therapeutic promise

**DOI:** 10.3389/fphar.2025.1735760

**Published:** 2026-02-27

**Authors:** Jaya Rautela, Anand Gaurav, Veeranoot Nissapatorn, Chung Keat Tan, Ana Paula Girol, Maria De Lourdes Pereira, Vannajan Sanghiran Lee

**Affiliations:** 1 Department of Pharmaceutical Sciences, School of Health Sciences and Technology, UPES, Dehradun, Uttarakhand, India; 2 Faculty of Health Sciences, Villa College, Malé, Maldives; 3 Faculty of Pharmaceutical Sciences, UCSI University, Kuala Lumpur, Malaysia; 4 Futuristic Science Research Center, School of Science, World Union for Herbal Drug Discovery (WUHeDD), Nakhon Si Thammarat, Thailand; 5 Research Excellence Center for Innovation and Health Products (RECIHP), Walailak University, Nakhon Si Thammarat, Thailand; 6 School of Healthy Aging, Aesthetics and Regenerative Medicine, Faculty of Medicine and Health Sciences, UCSI University, Kuala Lumpur, Malaysia; 7 Post Graduate Program in Structural and Functional Biology, Escola Paulista de Medicina (UNIFESP-EPM), Federal University of São Paulo, São Paulo, Brazil; 8 Department of Biology, Institute of Biosciences, Humanities and Exact Sciences (Ibilce), São Paulo State University (UNESP), São Paulo, Brazil; 9 Department of Medical Sciences, University of Aveiro, Aveiro, Portugal; 10 CICECO-Aveiro Institute of Materials, University of Aveiro, Aveiro, Portugal; 11 Center of Excellence for Quantum Information Science and Technology (COE QIST), Department of Chemistry, Faculty of Science, University Malaya, Kuala Lumpur, Malaysia; 12 Center of Excellence in Structural and Computational Biology, Department of Biochemistry, Faculty of Science, Chulalongkorn University, Bangkok, Thailand

**Keywords:** clinical transition, dual inhibitors, phosphodiesterase 10A, phosphodiesterase 1B, schizophrenia

## Abstract

Phosphodiesterase 1B (PDE1B) and phosphodiesterase 10A (PDE10A), members of the phosphodiesterase superfamily, are responsible for cyclic nucleotide hydrolysis, thereby regulating key intracellular signaling pathways such as cAMP response element-binding protein (CREB) activation and brain-derived neurotrophic factor (BDNF) gene transcription. Both enzymes are predominantly expressed in the brain and co-localize with dopamine receptors, positioning them as potential targets for addressing schizophrenia, a disorder characterized by dopamine system dysfunction. PDE1B inhibition enhances D1-receptor signaling, ameliorating negative symptoms and cognitive deficits, while PDE10A inhibition modulates D2-receptor activity, potentially alleviating positive symptoms. Together, these mechanisms suggest that targeting PDE1B and PDE10A could offer an innovative avenue for the comprehensive management of schizophrenia. Recent advancements in structural and synthetic methodologies have significantly facilitated the design of small-molecule PDE1B and PDE10A inhibitors. Among these, ITI-214 (PDE1 inhibitors) and MK-8189 and EVP-6308 (PDE10A inhibitors) have proceeded to clinical trials, demonstrating promising therapeutic agents. Furthermore, dual PDE1B/10A inhibitors remain underexplored, with only compound 2 undergoing limited preclinical evaluation for its pharmacological efficacy and safety. Studies published between 2014 and 2025 were retrieved from the PubMed, Web of Science, and Scopus databases, highlighting advances in PDE1B and PDE10A inhibitors. This review provides a detailed overview of the structural and synthetic strategies employed in developing PDE1B, PDE10A, and dual PDE1/10 inhibitors, with a focus on their binding sites and structure–activity relationships (SARs). By addressing the limitations of current candidates and emphasizing the need for dual inhibitors, this review aims to guide future research efforts toward the discovery of more selective, potent, and clinically viable PDE1B and PDE10A inhibitors for schizophrenia.

## Highlights

- PDE1B and PDE10A are critically implicated in schizophrenia pathophysiology through the disruption of cyclic nucleotides signaling pathways linked to positive, negative, and cognitive impairment.
- The binding interaction studies highlight critical active site residues in PDE1B (L388, F424, H373, F392, and Q421) and PDE10A (F729, I692, F696, Y683, F719, and Q726), which are key determinants for ligand binding and selectivity.
- Dysregulation of the cAMP/PKA/CREB/BDNF axis in schizophrenia can be modulated *via* dual PDE1B/PDE10A inhibition, offering a targeted therapeutic approach.
- Dual inhibitors such as compound 2 enhance cAMP signaling by simultaneously inhibiting PDE1B and PDE10A, showing promise in preclinical models.
- Clinical-stage inhibitors, ITI-214 (PDE1) and MK-8189 (PDE10A), highlight the translational potential of PDE inhibition in the therapeutic management of schizophrenia.

## 1 Introduction

Schizophrenia is a chronic, debilitating neuropsychiatric disorder that affects approximately 0.5%–1.0% of the global population (McGrath et al., 2004; Lane et al., 2018). It is clinically characterized by a group of symptom domains: positive symptoms (such as hallucinations and delusions), negative symptoms (including social withdrawal and apathy), and cognitive impairments (such as disorganized speech and impaired attention) (Hegarty et al., 1994; Insel, 2010; McCutcheon et al., 2023). In aggregate, these symptoms profoundly impair functional capacity, social integration, and independent living among affected individuals (Krause et al., 2018; McCleery and Nuechterlein, 2019).

Dopaminergic dysfunction is a core pathophysiological feature of schizophrenia, mediated through three major brain circuits: the mesolimbic, mesocortical, and nigrostriatal pathways (Luo and Huang, 2016). Hyperactivity in the mesolimbic pathway is associated with the emergence of positive symptoms such as hallucinations and delusions, while hypoactivity in the mesocortical pathway contributes to negative and cognitive symptoms. The nigrostriatal pathway, although primarily responsible for motor control, is often indirectly affected by D2 receptor antagonism and contributes to extrapyramidal side effects observed with antipsychotic treatment (Hietala et al., 1995; 1999; Abi-Dargham et al., 1998; Groenewegen, 2003).

Current antipsychotics primarily target D_2_ receptors and effectively reduce positive symptoms but show limited efficacy in ameliorating cognitive deficits and negative symptoms (Solmi et al., 2017; Cerveri et al., 2019). Moreover, their therapeutic utility is often constrained by side effects and poor long-term outcomes, highlighting the urgent need for mechanistically novel treatments that can effectively address the positive, negative, and cognitive symptoms of schizophrenia (Remington et al., 2016).

**FIGURE 1:**
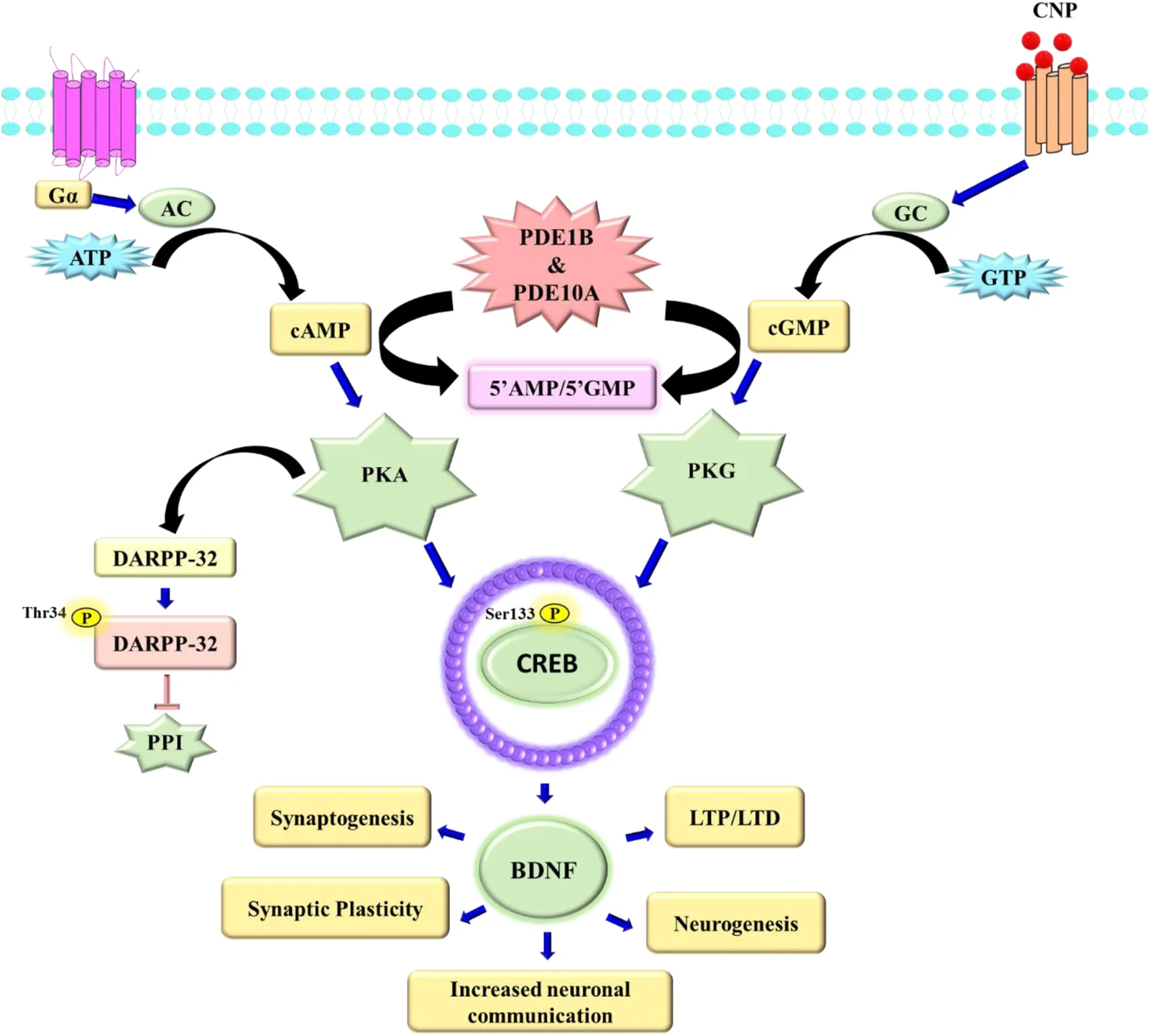
Schematic illustration of the intracellular signaling events controlled by PDE1 and PDE10 in neuronal systems. PDE1 activation reduces intracellular cAMP and cGMP levels downstream of AC, modulating PKA signaling and the phosphorylation of substrates such as DARPP-32, PP1, and CREB. PDE10 integrates dopaminergic inputs from D1/D2 receptor signaling and similarly regulates cAMP-dependent transcriptional events, including BDNF expression. AC, adenosine cyclase; ATP, adenosine triphosphate; PKA, cAMP-dependent protein kinase; DARPP-32, dopamine- and cAMP-regulated neuronal phosphoprotein; PP1, protein phosphatase-1; CREB, cAMP response element-binding protein; BDNF, brain-derived neurotrophic factor; CNP, C-type natriuretic peptide.

Phosphodiesterases (PDEs) comprise a group of intracellular catalysts originally identified in 1962 by Dr. Earl Sutherland and others*.* These enzymes preferentially catalyze the hydrolysis of phosphodiester linkages of cAMP and cGMP (Beavo, 1995; Nadur et al., 2021). Cyclic nucleotides are involved in major intracellular signaling pathways that modulate various physiological processes such as signal transduction, synaptic transmission, and hormonal regulation (Giembycz and Maurice, 2014; Zorn and Baillie, 2023; Kwiatkowski et al., 2024). The PDEs, particularly PDE1B and PDE10A, are abundant in the CNS (Table 1) and exert a crucial influence on the modulation of cyclic nucleotide signaling (Figure 1) (Lakics et al., 2010; Sharma et al., 2023)**.** In this context, PDE1B and PDE10A have emerged as key therapeutic targets in schizophrenia due to their regulatory roles in cAMP/cGMP signaling downstream of dopamine receptors (Al-Nema et al., 2021). By modulating the cAMP/protein kinase A (PKA)/CREB or cGMP/PKG/CREB pathway, these enzymes influence the phosphorylation of CREB at Ser133 and dopamine- and cAMP-regulated phosphoprotein of 32 kDa (DARPP-32) at Thr34, which are pivotal for the transcription of neurotrophic factors such as brain-derived neurotrophic factor (BDNF), thereby impacting neuroplasticity and synaptic integrity (Morimoto and Koshland, 1991; Silva et al., 1998; Mayr and Montminy, 2001; Tully et al., 2003; Rajput et al., 2009; Kandel, 2012; Kim et al., 2013; Shi et al., 2021).

**TABLE 1:** Overview of PDE isoforms, substrate specificity, brain expression, and biological effects.

PDE	Isoform	Substrate	Feature	Brain localization	Physiological effect
PDE1	PDE1A PDE1B PDE1C	cAMP/cGMP	Calcium/calmodulin regulated	Amygdala, cortex, midbrain, hippocampus, nucleus accumbens, cerebellum, thalamus, and olfactory bulb	Mediates cross-talk between calcium and cAMP/cGMP signaling pathways Regulates contraction and influences smooth muscle Locomotor influences Spermatogenesis Cardiac remodeling
PDE2	PDE2A	cAMP/cGMP	cGMP- and cAMP-specific	Amygdala, midbrain, hypothalamus, cortex, and striatum	Intermediate cyclic nucleotide signaling Modulates endothelial cell growth and permeability
PDE3	PDE3A PDE3B	cAMP/cGMP	cGMP-inhibited	Hippocampus and striatum	Regulates cardiac contraction and platelet aggregation Influences vascular and airway smooth muscle contraction Regulates inflammation, fibrosis, insulin response, and cell proliferation
PDE4	PDE4A PDE4B PDE4C PDE4D	cAMP	cAMP-specific	Cortex, midbrain, cerebellum, hippocampus, olfactory bulb, thalamus, and striatum	Regulates inflammatory responses and smooth muscle bronchodilation Controls cardiomyocyte contraction and neuronal functions Modulates fertility
PDE5	PDE5A	cGMP	cGMP-specific	Cerebellum, hippocampus, spinal cord, and cortex	Regulates vascular tone and cardiomyocyte contraction Influences the mitochondrial signaling pathways Controls the neuronal functions and platelet functions
PDE6	PDE6A PDE6B PDE6C	cGMP	Photo-receptor	Retinal rod, cone cells, and pineal gland	Facilitates retinal signal transmission and photoreaction in retinal cells Regulates melatonin release
PDE7	PDE7A PDE7B	cAMP	Rolipram-insensitive	Striatum, midbrain, cerebellum, cortex, thalamus, and hypothalamus	Activates T-lymphocytes Influences bronchoconstriction
PDE8	PDE8A PDE8B	cAMP	cAMP-specific	Striatum, hippocampus, cerebellum, olfactory bulb, cortex, thalamus, and midbrain	Activates T-cells Spermatogenesis Influences Leydig cell function and bronchoconstriction
PDE9	PDE9A	cGMP	cGMP-specific	Cortex, cerebellum, thalamus, hippocampus, amygdala, and striatum	Regulates the neuronal functions Involves in inflammation and bronchoconstriction
PDE10	PDE10A	cAMP/cGMP	cAMP-inhibited	Striatum, cortex, cerebellum, and hippocampus	Influences direct and indirect dopamine pathways Involves in neuronal functions Improves learning and cognation
PDE11	PDE11A	cAMP/cGMP	Dual substrate	Hippocampus	Spermatogenesis

Although cAMP and cGMP pathways generally promote neuroplasticity, their downstream effects can vary significantly depending on neuronal subtype, developmental stage, and disease state. For instance, in glutamatergic neurons, elevated cAMP may enhance synaptic plasticity and memory consolidation, while in GABAergic interneurons, excessive cyclic nucleotide signaling may disturb inhibitory balance, contributing to excitatory–inhibitory dysregulation. Similarly, cGMP-mediated pathways that promote long-term potentiation in the hippocampus may yield opposing effects in other circuits, depending on nitric oxide signaling and phosphatase activation. These dichotomous effects emphasize the need for spatially and temporally targeted modulation of PDE activity, particularly in the complex neuroarchitecture of the schizophrenic brain (Svenningsson et al., 2004; Neef and Palacios, 2021; Rombaut et al., 2021).

However, members of the PDE superfamily share a highly conserved catalytic domain, and the individual PDE isoforms diverge substantially in their substrate preferences, regulatory mechanisms, and regional expression patterns, which together influence their therapeutic relevance. Furthermore, the strong sequence homology within the catalytic core contributes to substantial challenges in achieving isoform-selective inhibition and often results in off-target engagement, a major factor underlying the clinical failure of PDE-targeted therapeutics (Goff et al., 2009; Demartinis et al., 2019). Nevertheless, isoform-specific variations in N-terminal regulatory domains, active-site architecture, and catalytic pocket dynamics impart distinct biochemical properties and differential inhibitor selectivity (Wang et al., 2007). Although multiple PDE isoforms are expressed in the central nervous system (Table 1), only a limited number of isoforms demonstrate a mechanistic alignment with the neurochemical pathways, particularly D_1_/D_2_, central to schizophrenia pathophysiology (Lusardi et al., 2024). PDE10A and PDE1B are highly enriched in MSNs, where they regulate D_1_/D_2_ receptor-dependent cAMP/cGMP signaling and, therefore, represent potential therapeutic targets. In contrast, other CNS-expressed isoforms, including PDE2, PDE4, PDE8, and PDE9, exhibit broader distribution profiles, regulate global rather than microdomain-restricted cyclic nucleotide signaling, and provide limited evidence for disease-specific relevance in schizophrenia. PDE2A shows regionally divergent alterations in schizophrenia, with reductions in the amygdala, cingulate cortex, and orbitofrontal cortex but elevated expression in the hippocampus, suggesting compensatory rather than pathogenic involvement. PDE4, despite its relevance to cortical cAMP signaling, is hindered by intolerable side effects such as nausea and vomiting, which have repeatedly limited clinical progression (Sharma and Purohit, 2023). PDE8 has limited mechanistic linkage to schizophrenia, with most available data restricted to aging and Alzheimer’s disease models. PDE9 similarly lacks connection to dopaminergic circuits and has demonstrated only modest or heterogeneous cognitive effects in early clinical studies. Collectively, the strong neuroanatomical enrichment of PDE1B and PDE10A within schizophrenia-relevant neuronal circuits, coupled with supportive preclinical evidence for PDE1B and more advanced clinical pharmacology for PDE10A, provides a compelling rationale to prioritize these isoforms over PDE2, PDE4, PDE8, and PDE9 in schizophrenia drug development. Furthermore, the mechanistic significance of these pathways, mediated by PDE1B and PDE10A, has guided medicinal chemistry efforts, which, when integrated with computational approaches, have substantially accelerated the discovery of potent, selective, and CNS-penetrant PDE1B and PDE10A inhibitors (Bhardwaj and Purohit, 2021; Sharma and Purohit, 2023; Sharma et al., 2023).

This review provides a comprehensive overview of the PDE superfamily, with particular emphasis on the PDE1B and PDE10A isoforms. We discuss their structural characteristics, binding sites, and the diverse synthetic strategies employed in the design of small-molecule inhibitors. The binding modes of known selective and dual PDE1/PDE10A inhibitors, as revealed by X-ray co-crystal structures, are examined alongside key challenges that complicate isoform-selective drug design, including narrow hydrophobic pockets, pKa-dependent binding preferences, and ligand inversion events that may lead to unintended cross-reactivity. Furthermore, we highlight studies demonstrating that structure-guided approaches, parallel-synthesis-driven SAR exploration, biological evaluation, and integrated ADME/PK analysis collectively advance the identification of potent and selective inhibitors. Notably, most design efforts have targeted the conserved glutamine residue, metal-binding site, and hydrophobic region, while emerging evidence indicates that the Q2 pocket in PDE10A also contributes significantly to isoform selectivity (Yu et al., 2020). Additionally, although the roles of PDE1B and PDE10A in dopaminergic regulation and schizophrenia are well established, relatively little attention has been paid to dual-target inhibition and the associated medicinal chemistry challenges. Recent studies exploring dual inhibition provide a novel perspective for therapeutic development as rational dual-target strategies enable the design of single molecules capable of simultaneously modulating both PDE1B and PDE10A activity. These approaches could overcome the limitations of isoform-selective inhibitors and may enhance therapeutic efficacy by concurrently targeting complementary enzymatic pathways implicated in schizophrenia. By integrating these strategies with structure-guided design and SAR analysis, dual inhibition emerges as a promising avenue for schizophrenia treatment. This review addresses critical gaps in the development of PDE1B and PDE10A inhibitors by integrating structural insights with a comprehensive analysis of chemotype studies. It emphasizes underexplored SAR patterns, persistent challenges in achieving isoform-selective inhibition, and emerging opportunities for rational dual-target strategies. The insights presented herein are intended to guide medicinal chemists in the rational design and optimization of both selective and dual PDE1B/PDE10A inhibitors for schizophrenia.

## 2 PDE isoforms

**FIGURE 2:**
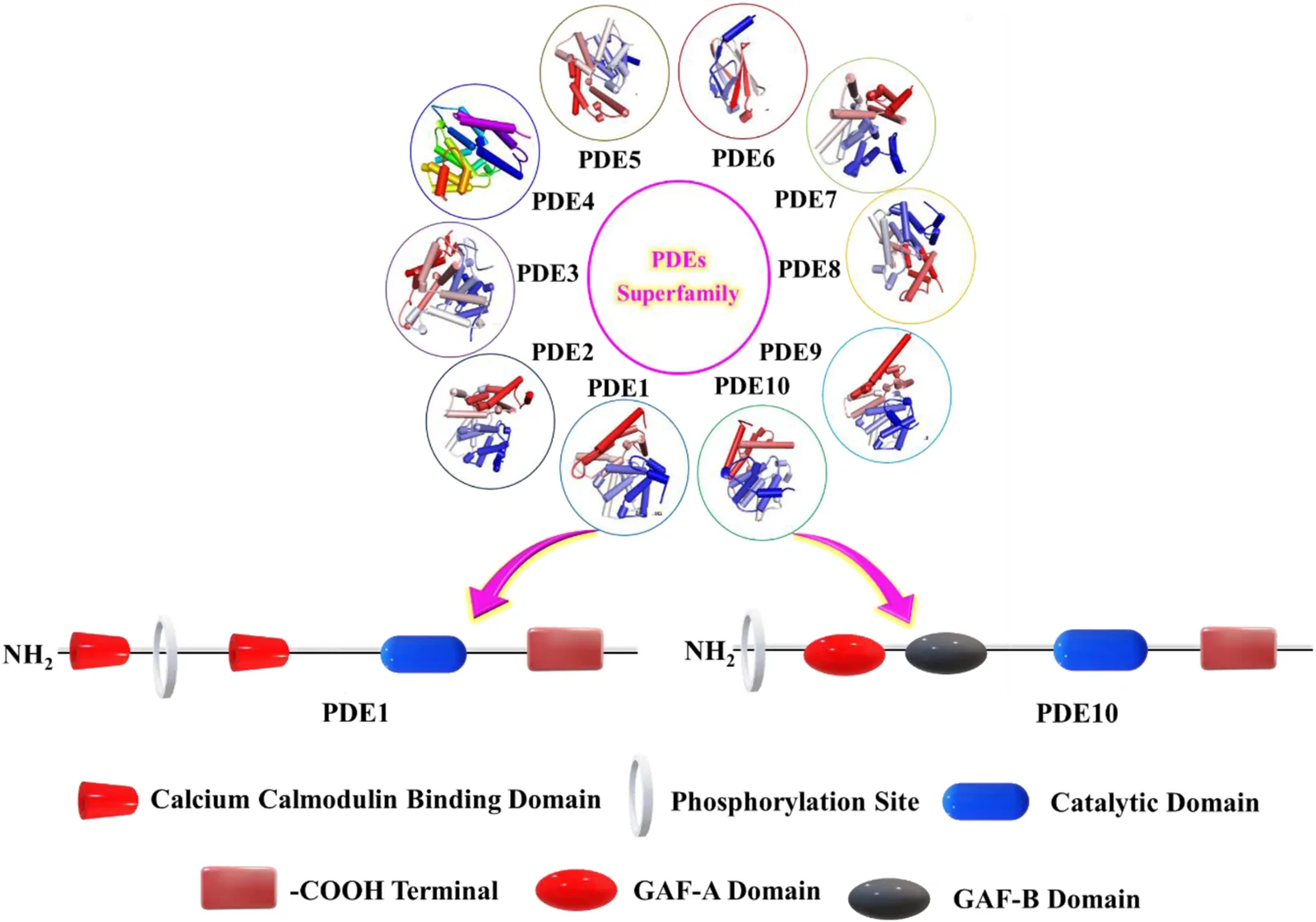
Alignment of PDE1 to PDE10, highlighting the conserved catalytic core and isoform-specific regulatory features. Linear schematics show the domain organization of PDE1 and PDE10 (calmodulin-binding region, phosphorylation site, catalytic domain, GAF-A, GAF-B, and C-terminus). PDB ID: PDE1, 5b25; PDE2, 6ezf; PDE3, 7l27; PDE4, 4kp6; PDE5, 3tge; PDE6, 5ml2; PDE7, 4pm0; PDE8, 7vtv; PDE9, 6a3n; PDE10, 6msa. The visualization was generated using Discovery Studio software 2024.

The human genome has identified 21 PDE genes and 11 morphologically relevant yet functionally different PDE families (PDE1-11). They differ from one another in their molecular sequence, substrate selectivity, tissue distribution, and cellular localization (Figure 2; Table 1) (Maurice et al., 2014; Ricciarelli and Fedele, 2018; Sadeghi et al., 2023). Each family contains discrete genes that subsequently produce over 100 PDE isoforms, categorized according to their encoding genes (PDE4A-C) and alternative mRNA splicing or transcriptional processing (PDE4D1-9) (Conti and Beavo, 2007; Halpin et al., 2008; Francis et al., 2011).

All the PDEs are composed of three functional regions: a C-terminal, an N-terminal, and a conserved catalytic region. The C-terminal is conserved among all PDEs, except for PDE6, showing 18%–46% sequence similarity (Figure 1). It is believed that the C-terminal is associated with dimerization and contains docking regions for PDE-selective kinases (Essayan, 2001). The N-terminal exhibits remarkable variability among PDEs, contributing to their intracellular localization. This domain also includes the calmodulin-binding region identified in PDE1, the GAF region observed in PDE2, 5, 6, 10, and 11, and the UCR1/UCR2 regions identified in PDE4 (Halpin, 2008; Keravis and Lugnier, 2012). The conserved catalytic region is highly homologous across families, with more than 50% sequence identity at the amino acid level (MacKenzie et al., 2000).

All PDEs contain approximately 350 amino acids in their conserved catalytic region with specific substrate affinity toward cyclic nucleotides. PDE families 4, 7, and 8 exhibit affinity for cAMP and PDE5, 6, and 9 are cGMP-specific, whereas the PDE families 1, 2, 3, 10, and 11 are promiscuous (Ke and Wang, 2007). The molecularly invariant catalytic region of PDEs determines the specificities toward nucleotide substrate, but the mechanism behind it is still unknown. However, the “glutamine switch mechanism” assumes that the γ-NH_2_ group of conserved ‘Q’ inside the catalytic site of PDEs can utilize two distinct alignments. In the first alignment, the hydrogen bond network facilitates ‘Q’ binding, leading to specificity toward cGMP, whereas in the second alignment, the network facilitates adenine binding, resulting in specificity toward cAMP. Conversely, in the dual-specific PDEs, the ‘Q’ side chain exhibits versatility by alternating between the two alignments, ensuring the specificity toward both the cyclic nucleotides. Table 1 summarizes the isoforms, substrate specificity, brain expression, and biological effects of PDEs (Zhang et al., 2004; Giembycz and Maurice, 2014; Prickaerts et al., 2017; Argyrousi et al., 2020; Mokra and Mokry, 2021; Sheng et al., 2022; Kamel et al., 2023; Kochoian et al., 2023).

### 2.1 Role of PDEs in schizophrenia

Among the eleven PDE families, PDE1B and PDE10A have emerged as especially relevant in the context of schizophrenia due to their high expression in striatal and cortical circuits governing dopaminergic neurotransmission (Nishi and Snyder, 2010; Al-Nema et al., 2021). PDE1B is enriched in the medium spiny neurons (MSNs) of the direct pathway, while PDE10A is highly expressed in the MSNs of both the direct and indirect pathways (Lakics et al., 2010). These enzymes modulate the cAMP/PKA/CREB signaling cascade, where cAMP activates PKA, leading to the phosphorylation of CREB at Ser133 and DARPP-32 at Thr34. Phosphorylation of CREB promotes expression of neuroprotective factors such as brain-derived neurotrophic factor (BDNF), essential for neuroplasticity and synaptic function (Tully et al., 2003; Rajput et al., 2009). Additionally, PKA-mediated inhibition of protein phosphatase-1 (PP1) alters AMPA and GABAA receptor phosphorylation, influencing neuronal excitability and circuit integrity (Figure 2) (Jancic et al., 2009; Merz et al., 2011). Therefore, PDE1B and PDE10A inhibitors are speculated to be potential targets for schizophrenia as they can address all three symptoms by activating CREB pathways.

Furthermore, genetic evidence also supports PDE involvement in schizophrenia susceptibility (Millar et al., 2005; Pickard et al., 2007; Al-Nema et al., 2022a; Al-Nema et al., 2022b). Notably, PDE10A has been mapped near the 6q26–27 chromosomal region, which is adjacent to 6q25, a locus repeatedly implicated in large pedigree-based linkage studies of schizophrenia. Lindholm et al. (2001) demonstrated significant linkage at marker D6S264 (LOD score 3.45), with fine-mapping in a 3,400-member Swedish pedigree revealing a haplotype in 6q25 that segregates with affected individuals, suggesting that this region may harbor susceptibility variants related to PDE signaling (Loughney et al., 1999; Soderling et al., 1999). Similarly, converging preclinical and genetic data have increasingly implicated PDE1B in schizophrenia pathophysiology. Reed et al. (2002) demonstrated that PDE1B knockout mice exhibit increased cyclic nucleotide signaling due to PDE1B inhibition, which significantly influences dopaminergic pathways and, consequently, affects the behavior and learning abilities of the mice. However, while these findings provide compelling mechanistic rationale, the translational trajectory from genetic association to clinical efficacy remains complex. Genetic studies often suffer from small effect sizes, phenotypic heterogeneity, and population stratification, limiting reproducibility across diverse cohorts. Moreover, clinical trials evaluating PDE1 and PDE10A inhibitors have yielded inconsistent outcomes, with some compounds demonstrating improvements in cognitive or negative symptoms, while others failed to achieve primary endpoints. Several clinical studies have demonstrated the tolerability, safety, and pro-cognitive effect of a selective PDE1B and PDE10A inhibitor in patients with schizophrenia (Li et al., 2016; Tsai et al., 2016; Macek et al., 2019; Layton et al., 2023). However, these favorable observations have not translated into consistent clinical efficacy, a limitation that is discussed in the forthcoming sections.

Despite these challenges, PDE inhibitors, particularly PDE1B and PDE10A inhibitors, have emerged as promising therapeutic targets due to their ability to modulate the cAMP/PKA/CREB signaling cascade. In contrast to antipsychotic agents, which predominantly alleviate positive symptoms of schizophrenia, PDE1B and PDE10A inhibitors hold promise in addressing the broader symptomatology, including the negative and cognitive symptoms that remain inadequately managed by current therapies (Sadeghi et al., 2023).

### 2.2 Targeting PDE1B and PDE10A isoforms in the therapeutic aspect of schizophrenia

PDEs, particularly PDE1B and PDE10A, are predominantly expressed in the striatum, a locus critically involved in dopaminergic neurotransmission. Their spatial distribution closely aligns with regions rich in dopaminergic innervation, implicating them in the regulation of dopamine-mediated signaling pathways central to neuropsychiatric disorders, particularly schizophrenia (Russwurm et al., 2015; Al-Nema and Gaurav, 2020). Owing to their overlapping yet distinct roles within dopaminergic circuits, both enzymes have emerged as potential targets for neuropsychiatric disorders such as schizophrenia (Garcia et al., 2014; Cardinale and Fusco, 2018; Suzuki and Kimura, 2018).

PDE1B has been implicated in the modulation of dopamine D1 receptor signaling, a pathway frequently disrupted in the negative and cognitive symptoms of schizophrenia. As a major enzyme responsible for the hydrolysis of cyclic nucleotides downstream of D1 receptor activation, inhibition of PDE1B is postulated to enhance D1-mediated signaling, thereby ameliorating negative and cognitive symptoms (Heckman et al., 2016; Al-Nema and Gaurav, 2020). Conversely, PDE10A is uniquely expressed in both direct and indirect pathway MSNs of the striatum, with predominant expression in the indirect pathway MSNs. Given its role in modulating D2 receptor signaling, inhibition of PDE10A has shown promise in attenuating the hyperdopaminergic activity associated with the positive symptoms of schizophrenia, such as delusions and hallucinations (Grauer et al., 2009; Hikida et al., 2010).

Despite the promise of selective PDE1B and PDE10A inhibitors, several mechanistic gaps and inconsistencies remain that need further exploration of isoform-targeted strategies. First, PDE1B inhibitors improve cognitive and negative symptoms but fail to address positive symptoms. Conversely, PDE10A inhibitors primarily target positive symptoms and may induce extrapyramidal effects due to overactivation of the indirect pathway. Additionally, the overlapping distribution of PDE1B and PDE10A in MSNs, coupled with complex interactions between direct and indirect dopaminergic pathways, suggests that the net effects of single isoform inhibition may be context-dependent and insufficient for comprehensive symptomatic management. Collectively, these observations highlight the need to explore integrated strategies, such as dual PDE1B/PDE10A inhibition, to achieve balanced modulation of dopaminergic signaling and comprehensive symptomatic management across all three domains of schizophrenia.

### 2.3 Comparison of selective versus dual PDE1B/PDE10A inhibition

Selective inhibition of PDE1B or PDE10A enabled targeted modulation of distinct intracellular signaling cascades. Preclinical investigations have demonstrated that selective PDE1B inhibition enhances D1 receptor-mediated signaling, contributing predominantly to improvements in cognitive and negative symptoms of schizophrenia. However, these inhibitors exhibit limited efficacy against positive symptoms (Dyck et al., 2017). Conversely, PDE10A inhibitors have shown potential in alleviating positive symptoms by modulating striatal signaling *via* the indirect pathway; nevertheless, their impact on cognitive and negative symptoms remains inconclusive (Arakawa et al., 2016). Since schizophrenia is a complex disorder, its management with either selective PDE1B or PDE10A inhibitors has yielded suboptimal therapeutic outcomes. Moreover, PDE10A inhibitors, by attenuating activity in the indirect pathway, mimic D2 receptor antagonists, thereby increasing the risk of extrapyramidal side effects. These adverse events have contributed to the discontinuation of several PDE10A candidates in clinical development (Grauer et al., 2009; Heckman et al., 2016).

Given the complementary roles of PDE1B and PDE10A within striatal dopaminergic circuits, selective inhibition of either enzyme addresses only specific symptom domains of schizophrenia, limiting overall therapeutic efficacy. PDE1B inhibition primarily enhances D1 receptor-mediated signaling, improving cognitive and negative symptoms, whereas PDE10A inhibition targets D2 receptor-mediated hyperactivity to alleviate positive symptoms. However, single-isoform modulation may yield context-dependent and suboptimal outcomes due to overlapping enzyme distribution and the intricate interplay between direct and indirect striatal pathways, and PDE10A inhibitors have been associated with extrapyramidal side effects. Dual PDE1B/PDE10A inhibition offers a mechanistically integrated approach, simultaneously potentiating D1-mediated signaling and tempering D2 overactivity, thereby enabling balanced regulation of striatal output. This strategy also coordinates downstream cyclic nucleotide-dependent cascades, including PKA-mediated phosphorylation of ERK, CREB, and DARPP-32, optimizing synaptic plasticity, neuronal excitability, and behavioral outcomes, and providing a rationale for comprehensive symptom management with reduced off-target effects.

Clinical experience with currently approved PDE inhibitors highlights the relevance of isoform selectivity in mitigating side effects (Bondarev et al., 2022). To date, no selective PDE1B or PDE10A inhibitors have been approved for clinical use, whereas existing PDE inhibitors have been associated with adverse events linked to their isoform profiles. For instance, PDE4 inhibitors such as roflumilast, used in COPD, frequently induce gastrointestinal and CNS-related side effects, including diarrhea, nausea, headache, insomnia, and weight loss (Janjua et al., 2020; Rhee and Kim, 2020; Goonathilake et al., 2022), reflecting widespread PDE4 expression in immune, gastrointestinal, and neural tissues. PDE5 inhibitors such as sildenafil and tadalafil generally exhibit better tolerability but can cause headache, flushing, dizziness, and visual disturbances due to partial inhibition of PDE6 in the retina (Wallis, 1999; Hamzehnejadi et al., 2022). Similarly, PDE3 inhibitors such as milrinone and cilostazol demonstrate cardiovascular adverse effects, including arrhythmias, tachycardia, and palpitations, attributable to PDE3 expression in myocardium and vascular smooth muscle (Kuzmiszyn et al., 2022). Collectively, these clinical observations highlight that isoform-specific targeting is essential not only for efficacy but also for minimizing off-target side effects, emphasizing the need for early-stage ADME/PK and selectivity profiling in the development of novel dual PDE1B/PDE10A inhibitors.

Selective PDE1B and PDE10A inhibitors can specifically target one isoform, which allows them to address a particular symptom of schizophrenia. Although there is substantial evidence supporting the role of PDE1B and PDE10A inhibition in schizophrenia, designing highly selective inhibitors for PDE1B and PDE10A has proven challenging due to the high degree of structural similarity between the catalytic sites of these enzymes. This similarity, including conserved residues such as glutamine, metal-binding sites, and hydrophobic regions within the binding pockets, often results in cross-target activity, limiting the success of selective therapies. Schizophrenia is a complex disorder with positive, negative, and cognitive symptoms, and selective inhibition of either PDE1B or PDE10A may effectively target only one of these domains, making comprehensive treatment difficult and contributing to treatment failure.

To overcome this limitation, dual inhibition of PDE1B and PDE10A has emerged as a promising strategy. By simultaneously targeting both enzymes, dual inhibitors have the potential to address multiple symptom domains of schizophrenia. Although dual PDE1B/PDE10A inhibitors are still in early stages of development, preliminary studies have demonstrated encouraging results. For example, a dual inhibitor, Zinc41306568, effectively prevented and reversed ketamine-induced schizophrenia-like behavioral alterations in rats. Unlike selective PDE1B or PDE10A inhibitors, which typically modulate either positive, negative, or cognitive symptoms, Zinc41306568 suppressed ketamine-induced hyperlocomotion (a model of positive symptoms), attenuated social isolation (negative symptoms), and improved recognition memory in the novel object recognition test (cognitive symptoms). These findings suggest that dual PDE1B/PDE10A inhibitors, such as Zinc41306568, represent a promising approach for comprehensive treatment of schizophrenia. However, further optimization of Zinc41306568 is required to enhance selectivity, reduce effective doses, and optimize its therapeutic profile (Al-Nema et al., 2021).

In this context, dual inhibition of PDE1B and PDE10A emerges as a promising therapeutic strategy. By concurrently enhancing D1 receptor-mediated transmission *via* PDE1B inhibition and attenuating D2 receptor hyperactivity through PDE10A inhibition, a dual inhibitor may achieve balanced modulation of both direct and indirect striatal pathways. Such an approach holds potential for the comprehensive symptomatic relief across the schizophrenia spectrum, including positive, negative, and cognitive symptoms, while minimizing dopaminergic side effects associated with conventional antipsychotics.

## 3 PDE1

PDE1 is a dual substrate enzyme and was first extracted from rat (Kakiuchi and Yamazaki, 1970) and bovine (Cheung, 1970) brain tissue in 1970. It is the only PDE with Ca^2+^-mediated stimulation through calmodulin and is distinctively aligned to facilitate the crosstalk between calcium and cAMP/cGMP cascades (Hayes et al., 2021). PDE1 is categorized into three isoenzymes: PDE1A, PDE1B, and PDE1C. The distribution of these isoenzymes varies across the body, PDE1A and PDE1B are highly expressed within the brain, while PDE1C is found within the lungs, heart, and bladder (Lakics et al., 2010). PDE1B is the isoform with the highest expression levels in the striatum and cortex, and its ability to hydrolyze cAMP (Km: 12 μM) and cGMP (Km: 1.2 μM) has made it a research hot spot for schizophrenia (Goraya and Cooper, 2005). Additionally, PDE1B is co-localized with dopamine receptors and regulates the dopaminergic signaling (Lakics et al., 2010). Given that the negative symptoms and cognitive deficits noted in schizophrenia are linked with reduced D1-dopaminergic signaling, PDE1B inhibitors are considered promising molecules for schizophrenia due to their potential to elevate D1-dopaminergic signaling (Figure 2) (Winterer, 2006). Across the recent decade, numerous investigations have been undertaken, developing the PDE1B in schizophrenia (Reed et al., 2002; Pekcec et al., 2018). However, no approved PDE1 inhibitor for schizophrenia has yet reached the market.

### 3.1 Insight into PDE1 inhibitors

**FIGURE 3:**
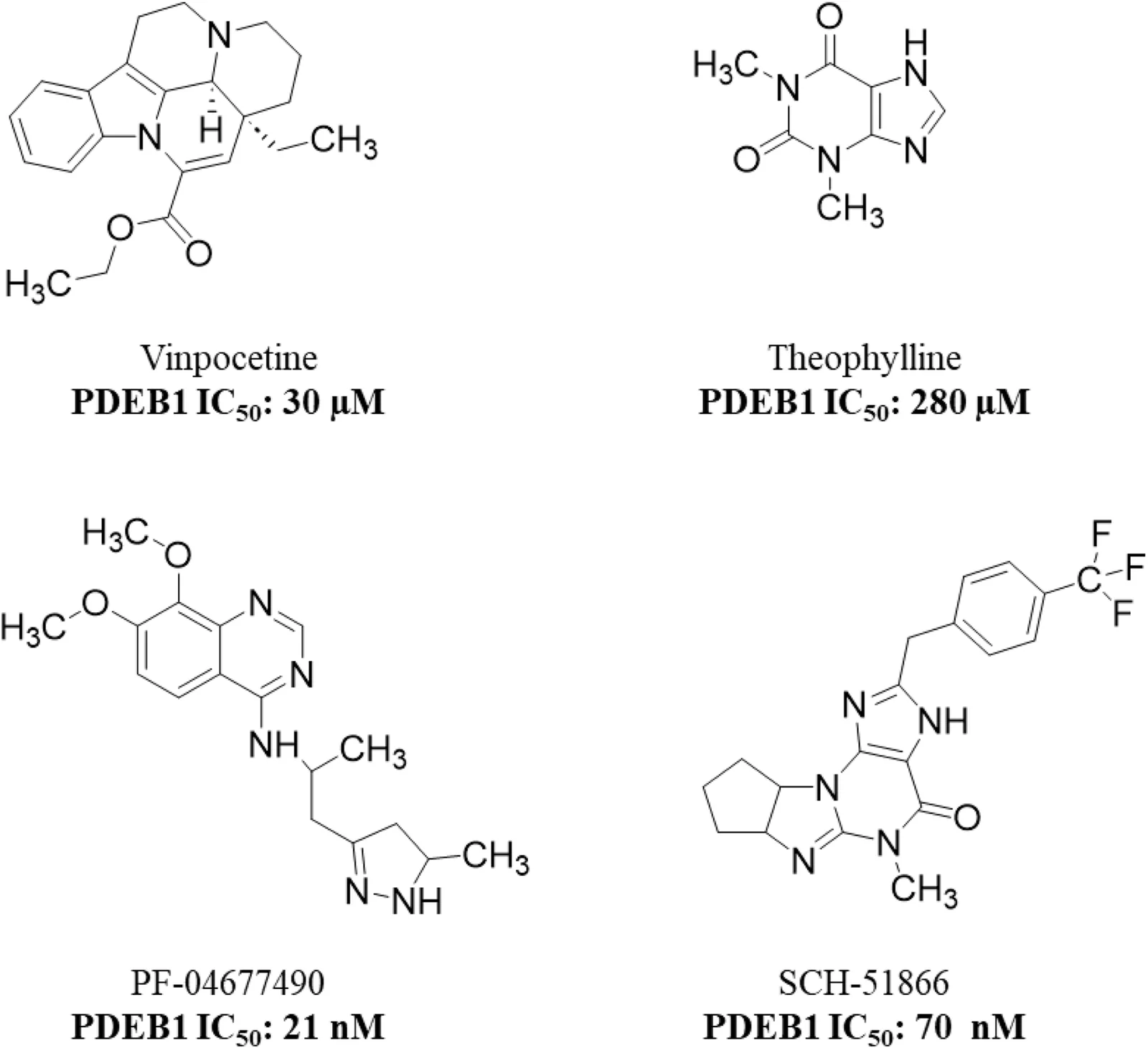
Chemical structures of early PDE1 inhibitors highlighting their inhibitory potency.

PDE1 is one of the earliest identified and most extensively studied PDEs; however, no approved PDE1 inhibitor has been developed to date due to a lack of specificity and efficacy. Several molecules, such as theophylline, vinpocetine, SCH-51866, and PF-04677490, have been investigated as PDE1 inhibitors; however, they exhibit either weak or non-selective PDE1 inhibition (Figure 3) (Hindmarch et al., 1991; Ukena et al., 1993; Vemulapalli et al., 1996). This has prompted continued efforts to discover potent and selective PDE1 inhibitors, and some PDE1 inhibitors have entered clinical trials (Table 2). Some of these efforts are described below.

**TABLE 2:** Small-molecule PDE1 and PDE10A investigated in clinical trials for schizophrenia (data as per ClinicalTrials.gov).

PDE selectivity	Drug name	Sponsor	Chemical structure	NCT	Dosing and treatment	Subject	Enrolment
PDE1	Lenrispodun phosphate (ITI-214)	Takeda	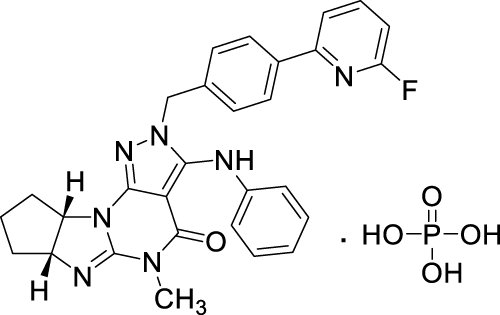	NCT01900522	Undisclosed oral dose, daily for a total of 14 days	Healthy and stable schizophrenia participants	76
PDE10	EVP-6308	FORUM Pharmaceuticals Inc	Structure undisclosed	NCT02037074	Undisclosed ascending oral dose for 14 days	Schizophrenia patients	40
	MK-8189 **(compound 77)**	Merck Sharp & Dohme LLC	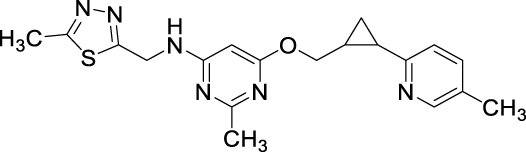	NCT05406440	Orally 48, 60, and 80 mg, once daily for 17 days.	Schizophrenia patients	53
				NCT04624243	Orally 8, 16, and 24 mg, once daily	Subjects with acute episode of schizophrenia	500
	MP-10/PF-2545920	Pfizer	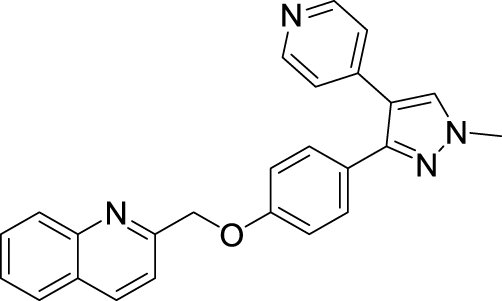	NCT00570063	Orally 15 mg, twice a day for 21 days	Schizophrenia patients	35
	TAK-063	Takeda	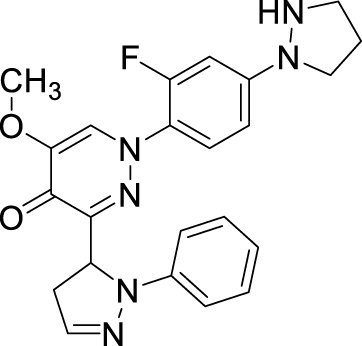	NCT02477020	Orally 20 mg, once daily for up to 6 weeks	Schizophrenia patients	164
	OMS-824	Omeros Corporation	Structure undisclosed	NCT01952132	Undisclosed dose daily for 14 days	Stable schizophrenia patients	40
	Papaverine	University of Copenhagen	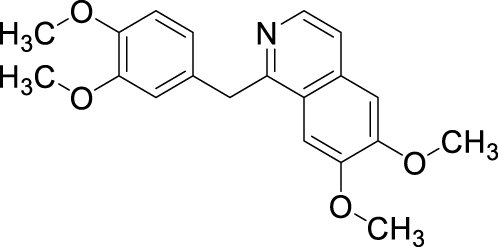	NCT01813955	Orally 300 mg for 1 month	Schizophrenia patients	5

#### 3.1.1 Pyrazolo-pyrimidinone derivatives

**FIGURE 4:**
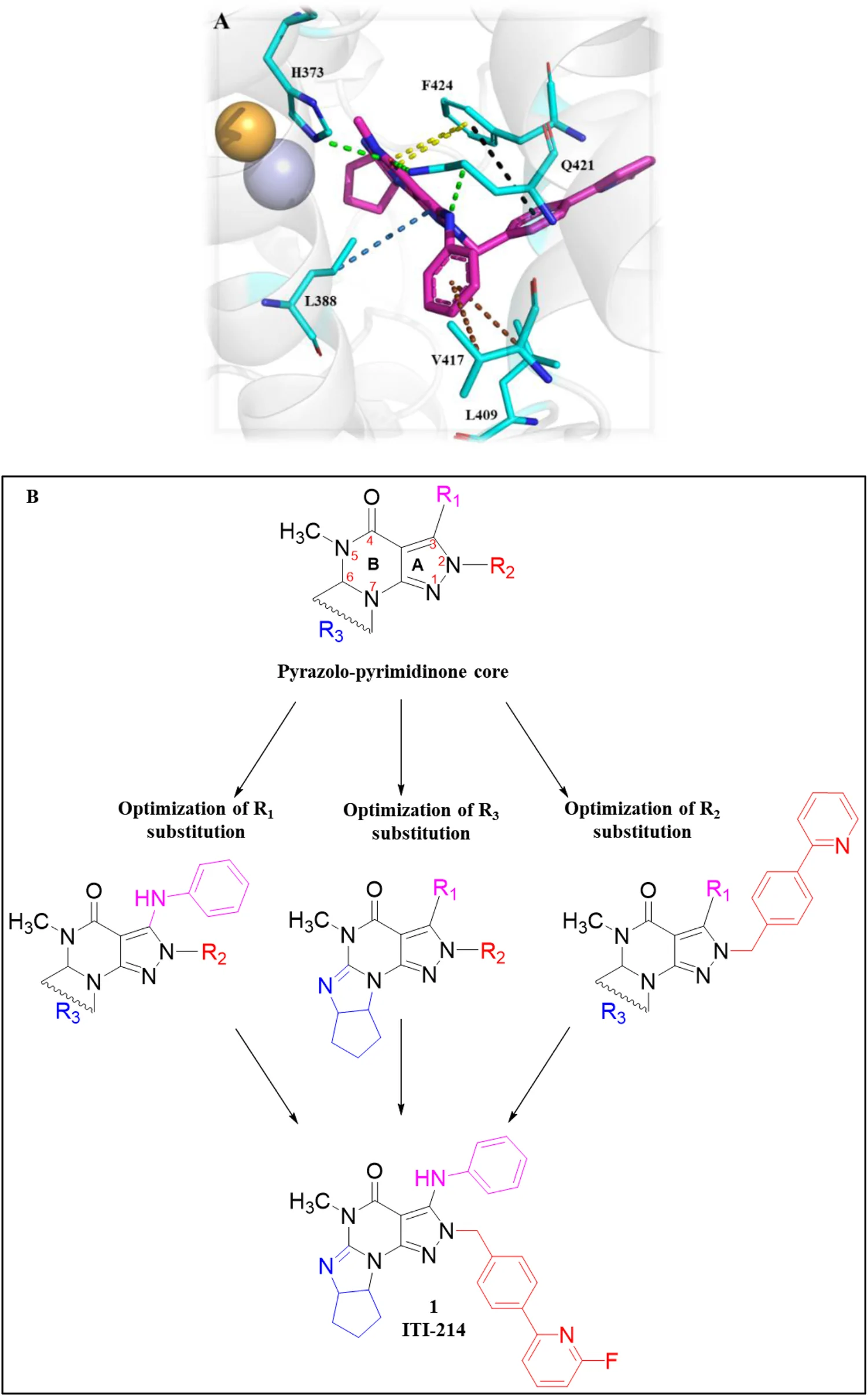
**(A)** Binding interaction of Compound **1** with PDE1B (PDB: 5b25), visualized using PyMOL. Key interactions include hydrogen bonding, π–π stacking, and hydrophobic interactions. The Zn^2+^ (pale yellow) and Mg^2+^ ions (light blue), spheres, occupy their bimetal-binding positions. The color representation of interactions is as follows: conventional hydrogen bond (green), π–π stacking (yellow), π–sigma (sky-blue), π–π T-shaped (black), π–alkyl (brown). **(B)** Structural optimization strategy for the 5-methyl-3H-pyrazolo[3,4-d] pyrimidin-4(5H)-one scaffold leading to Compound **1 (ITI-214).** Systematic modification of the R_1_, R_2_, and R_3_ substituents is illustrated to highlight structural features that improved PDE1B affinity.

**SCHEME 1:**
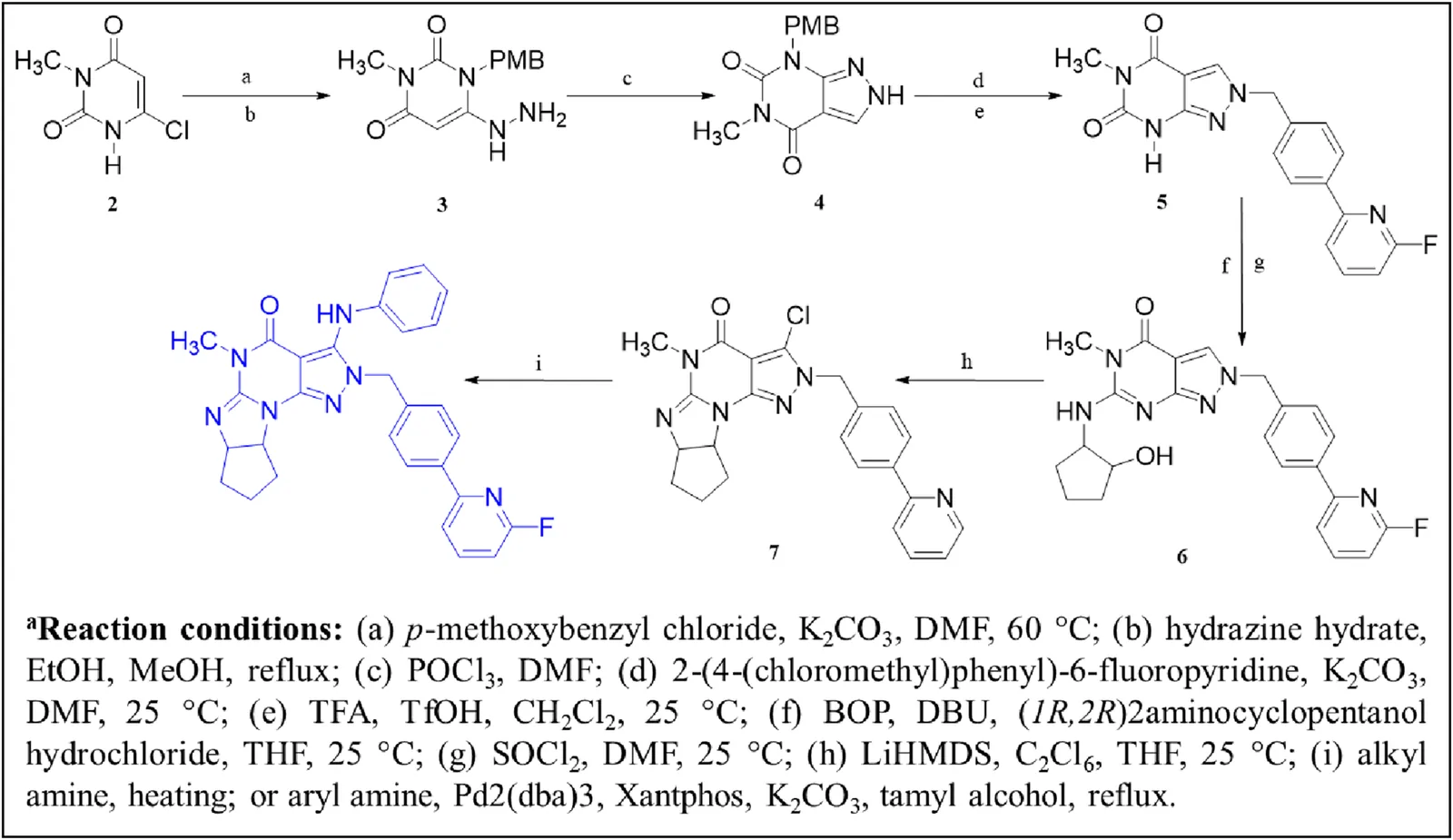
Synthesis of Compound **1**, as reported and executed by Li et al. (2016) *.*

Li et al. (2016) identified a PDE1 inhibitor clinical candidate, Compound **1 (ITI-214)**, *via* systematic pyrazolo-pyrimidinone scaffold optimizations. Compound **1** showed picomolar potency (Ki: 58 pM), excellent selectivity (2759-fold selective over PDE4), and good *in vivo* efficacy in the rat novel object recognition (NOR) test and has been nominated for clinical development. The optimization that led to ITI-214 involved changing substitution patterns at four distinct positions within the pyrazolo-pyrimidinone scaffold: N-2, C-3, C-6, and N-7. Fusing six-membered rings at positions C-6 and N-7 substantially improved PDE1 potency. However, reducing the size of the 6-membered rings to 5-membered rings significantly enhanced inhibitory activity and facilitated additional interactions with PDE1. The best improvement of potency and selectivity at this position was achieved with a cyclopentyl-fused 5-membered ring. Notably, incorporating a 2-(4-methyl-phenyl) pyridine sidechain at the N-2 position resulted in good PDE1 inhibitory potency and enhanced selectivity against PDE4. Amidst the SAR analysis, multiple substitutions were incorporated at the C-3 position. They discovered that larger alkyl groups, such as 2-methylbutyl-NH and cyclohexyl-NH, could enhance PDE1 inhibitory potency. These findings led to the identification of 2-(4-ethylphenyl)-6-fluoropyridine at the C-3 position as the optimal substitution. The combination of optimal substitutions at N-2, C-3, C-6, and N-7 as discussed above is epitomized by Compound **1** with a PDE1 Ki of 58 picomolar, which is over 100-fold more potent than other derivatives. ITI-214 also possesses 2759-fold selectivity for PDE1 over PDE4 (Figures 4A,B). Crystallographic X-ray examination of Compound **1** with PDE1B disclosed divergent interactions with the enzyme. The orientation of Compound **1** inside the catalytic cavity is inverted relative to that of cGMP. The pyrazolo-pyrimidinone core forms hydrophobic interactions and is stabilized by a “hydrophobic clamp.” The C-3 phenylamino group of the A-ring is deeply buried in a hydrophobic sub-pocket, establishing interactions with F392, L409, L388, and V417. The exocyclic C=O moiety of the B-ring and the C-3 amino group of the A-ring formed bidentate H-bonds with conserved Q421 (Figures 4A,B). The molecular weight (MW) of Compound **1** is 507.56 g/mol, exceeding the 500 g/mol threshold of Lipinski’s rules, suggesting potential synthetic challenges (Daina et al., 2017). Therefore, to efficiently synthesize Compound **1**, Compound **2** was secured with a para-methoxybenzyl (PMB) group, followed by the replacement of chlorine to produce Compound **3.** In the presence of DMF, Compound **3** reacted with POCl_3_ to yield Compound **4.** Subsequent benzylation of intermediate Compound **4**, followed by its deprotection, resulted in the formation of N_2_-substituted Compound **5.** Direct amination of Compound **5** mediated by BOP afforded Compound **6.** Further cyclization and chlorination of Compound **6** yielded Compound **7**, which, through a palladium-catalyzed cross-coupling reaction, produced the target molecule, Compound **1** (Scheme 1) (Li et al., 2016).

#### 3.1.2 Thieno[3,2-e]triazolo[1,5-c]pyrimidinone derivatives

**FIGURE 5:**
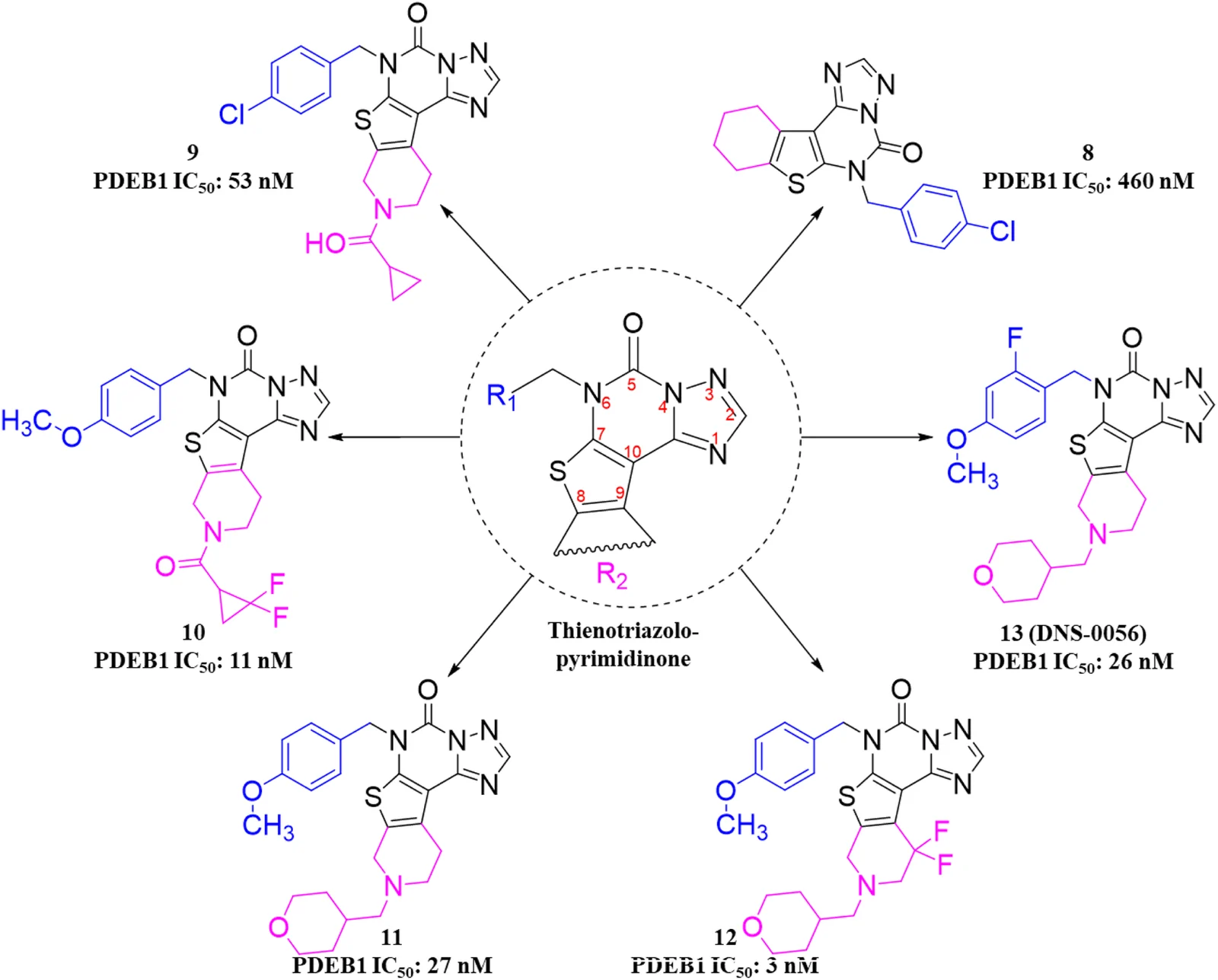
Structural optimization strategy for Compound **13**. Substitution patterns at R_1_, R_2_, and R_3_, respectively, highlight the effects of heterocyclic, alkyl, and fluoroalkoxy substituents on potency.

**FIGURE 6:**
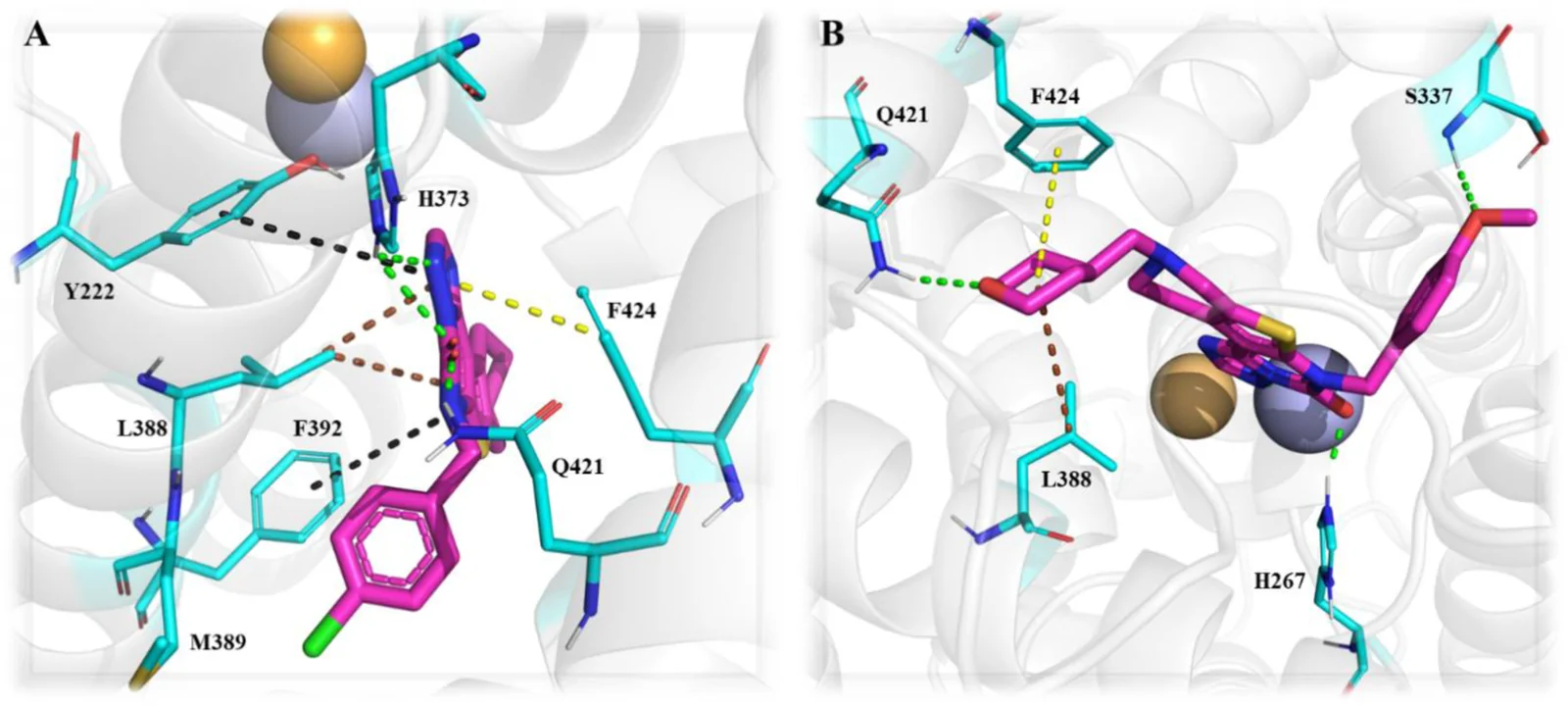
**(A)** Binding interaction of Compound **8** with PDE1B (PDB: 5UP0), highlighting ligand orientation within the active site, and positioning of hydrophobic and solvent-exposed moieties. **(B)** Binding interaction of Compound **11** with PDE1B (PDB: 5UOY), showing a similar binding mode with slight orientation differences in substituents (pyran-methyl group occupying a hydrophobic pocket) and the pyran oxygen forming a stabilizing hydrogen bond.

**SCHEME 2:**
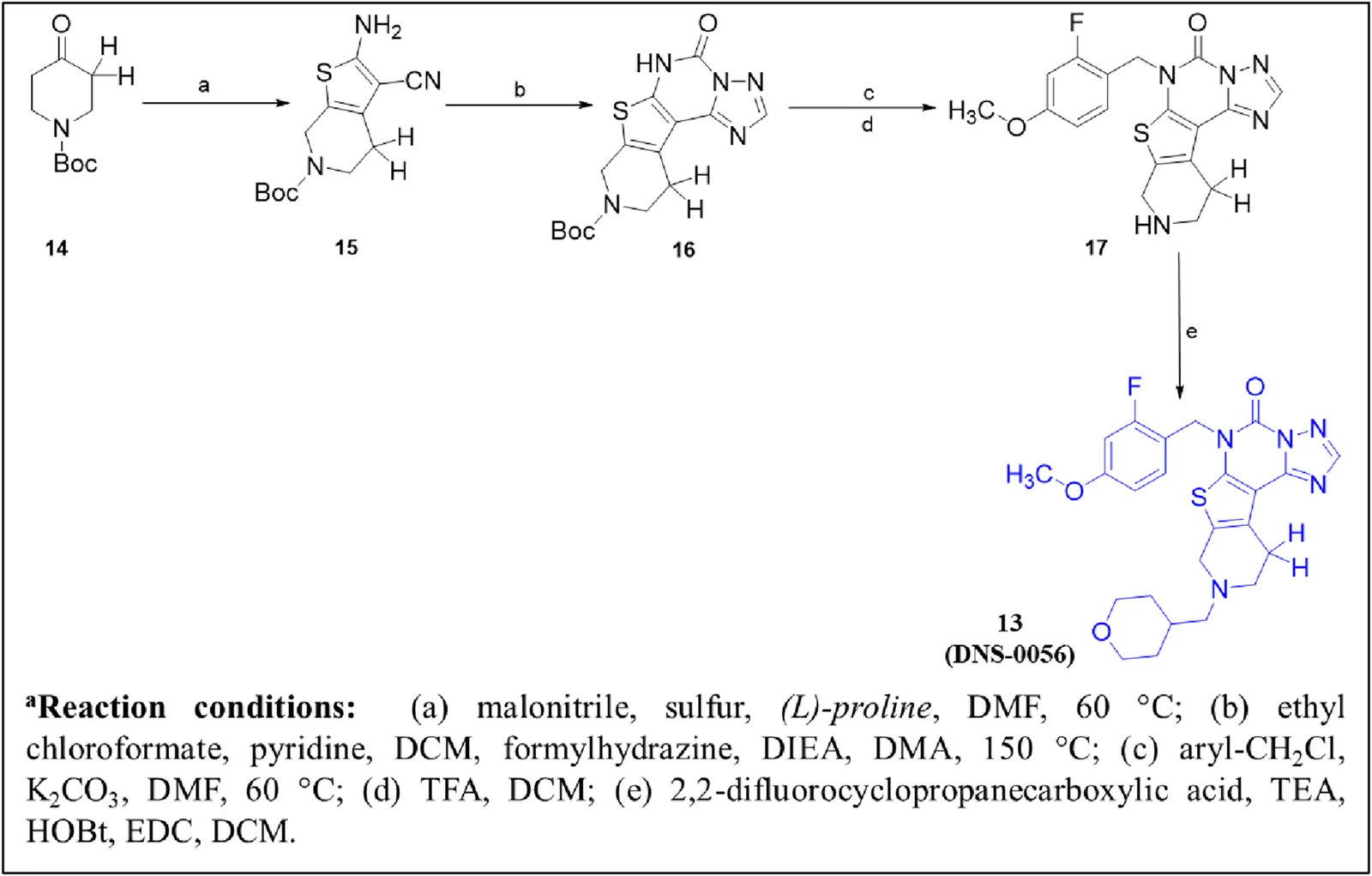
Synthesis of Compound **13**, as reported and employed by Dyck et al. (2017) *.*

The design and synthesis of thienotriazolopyrimidinone derivatives as PDE1 inhibitors were described later, focusing on various substitutions at positions C-6, C-8, and C-9 (Dyck et al., 2017). This led to Compound **13 (DNS-0056),** a nanomolar (IC_50_: 26 nM), orally bioavailable (90%), and brain-penetrating (maximum brain plasma ratio: 1.7) PDE1B inhibitor with good memory-enhancing effects in the rat NOR test. Initially, lead **8** was identified as a potential PDE1B inhibitor by screening a compound library. Further modifications, particularly the fusion of a cyclopropyl-pyridine-methanone ring at the C-8/C-9 positions and chlorobenzyl substitution at the C-6 position, led to Compound **9**, exhibiting enhanced PDE1B inhibitory activity with moderate hERG activity. However, the incorporation of difluorocyclopropyl-pyridin-methanone and methoxybenzyl substitutions at positions C-6/C-8 and C-9, respectively, led to Compound **10**, which not only inhibited PDE1B effectively but also exhibited potent inhibition of PDE10 (IC_50_: 59 nM). Therefore, pyran-methyl-pyridine and methoxy-methylbenzene were substituted at the same positions to enhance selectivity toward PDE1B, leading to Compound **11.** Improved potency and selectivity of Compound **11** toward PDE1B suggest that further substitution, such as difluoro-pyran-methyl-pyridine and methoxybenzyl at the C-6/C-8 and C-9 positions, can be employed to develop more potent PDE1B inhibitors. Such substitutions led to Compound **12** with excellent nanomolar inhibitory activity. However, Compound **12** was unfortunately identified as a substrate of multidrug resistance protein 1 (MDR1, P-gp) in Madin–Darby canine kidney (MDCK) cells with an efflux ratio greater than 5, which precluded its development as a candidate for CNS drugs. Ultimately, Compound **13 (DNS-0056)** with pyran-methyl-pyridine and fluoro-methoxybenzyl substitutions at positions C-6/C-8 and C-9, respectively, emerged as the most potent derivative and was not a substrate of MDR1 (efflux ratio: 1.0) (Figure 5). Evaluation of Compound **8** co-crystallized with unveiled several significant interactions. The carbonyl group of Compound **8** formed a polar interaction with Q421 and H373. This orientation of Compound **8** within the catalytic domain positioned the 4-chlorobenzyl moiety within a newly created lipophilic pocket, arising from the rotation of M389. Additionally, the cyclohexyl ring of Compound **8** protrudes into a solvent-exposed region, contributing to its specificity toward other PDEs (Figure 6A). Furthermore, Compound **11** bound to the enzymatic region of PDE1B exhibited binding mode similarity with Compound **8**, albeit with some differences, such as the pyran-methyl group of Compound **11** sticking out into a pocket made up of P408, F392, and T271. Additionally, the oxygen group of pyran formed a hydrogen interaction with Q421 (Figure 6B). The synthetic accessibility score and MW of DNS-0056 were 3.09 and 483.56 g/mol, respectively, lower than those of ITI-214; this indicates a comparatively simpler synthesis approach and fewer synthetic steps for DNS-0056 compared to ITI-214. The synthesis of DNS-0056 starts with treating Boc-protected piperidinones **14** with **15**. Subsequently, utilizing pyridine under standard conditions, the triazolo-pyrimidinone ring was incorporated, yielding Compound **16**. Compound **17** was obtained through N-alkylation. Substitution on the piperidine nitrogen atom was achieved through reductive alkylation under standard conditions, resulting in the target Compound **13** (Scheme 2) (Dyck et al., 2017).

#### 3.1.3 Pyrazolo[3,4-d]pyrimidone derivatives

**FIGURE 7:**
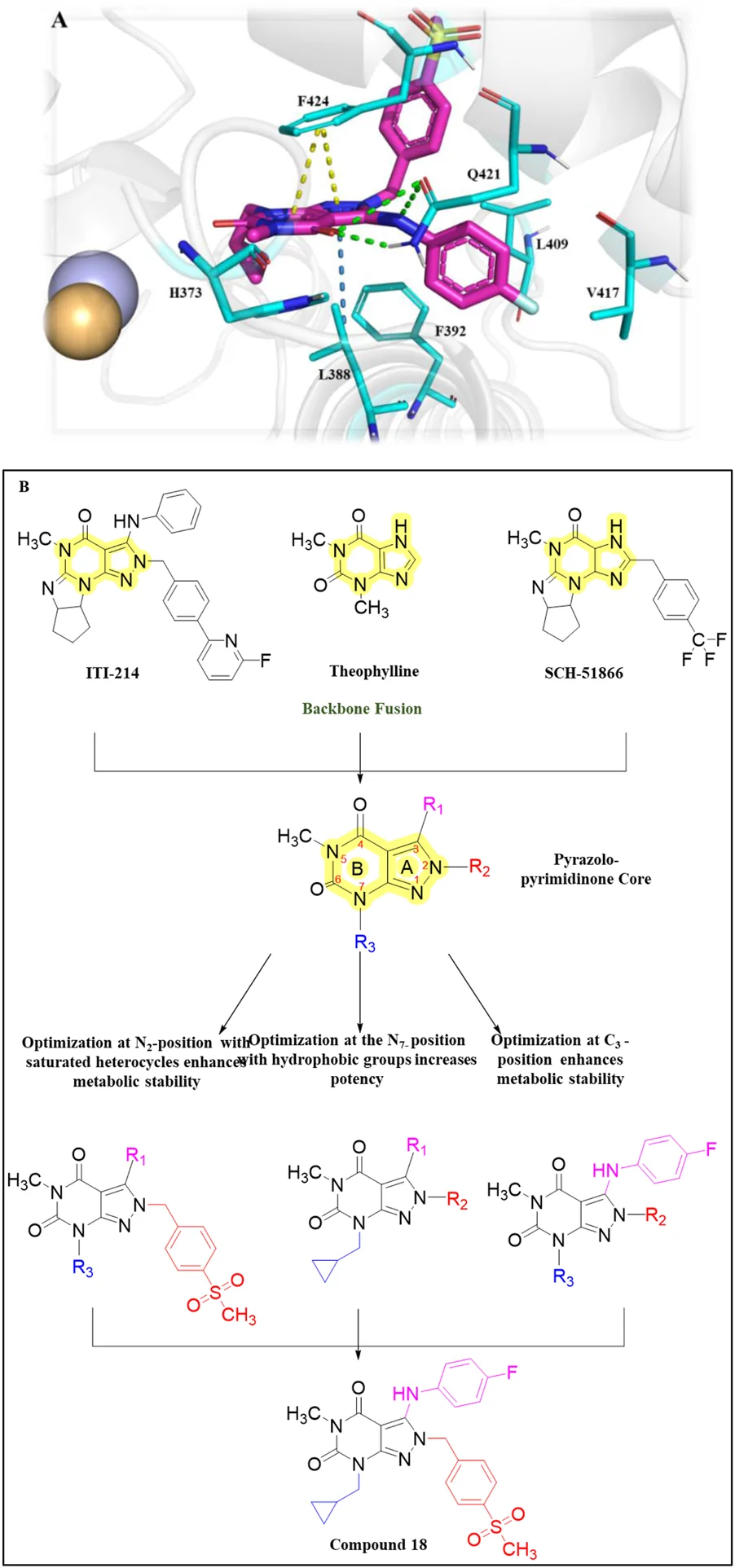
**(A)** Binding interaction of Compound **18** with PDE1B (PDB: 5b25) showing ligand orientation within the active site, hydrogen bonding of the pyrazolo-pyrimidone core with Q421, and positioning of hydrophobic moieties toward F424/F427. **(B)** Structural optimization steps on the discovery of Compound **18.** Incorporation of small hydrophobic N-7, sulfonyl N-2, and (4-fluorophenyl) C-3 groups, respectively, led to potent (IC_50_: 21 nM), selective (>480-fold vs. PDE8), and stable (T_1/2_: 28.5 min) PDE1 inhibition.

**SCHEME 3:**
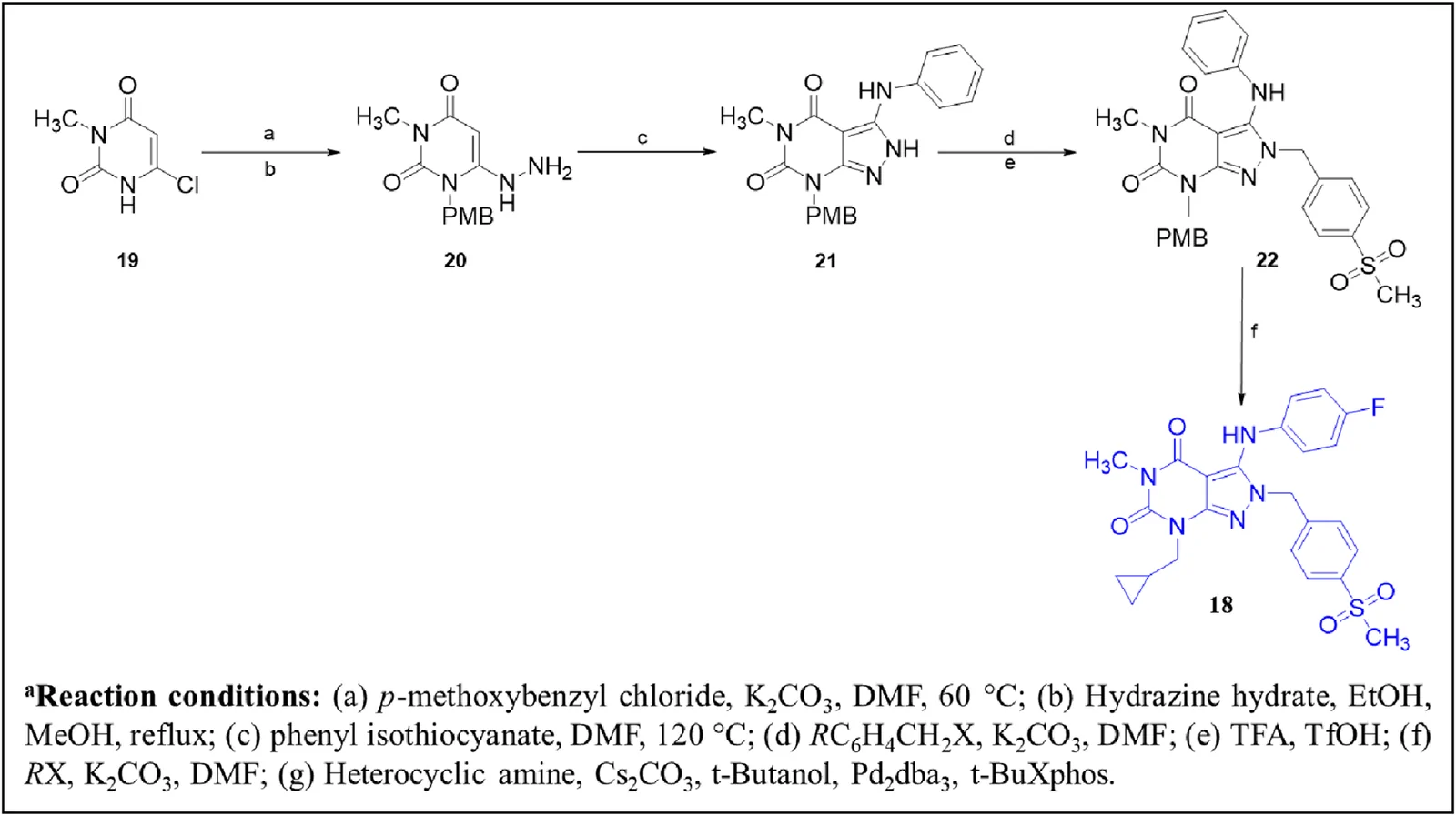
Synthesis of Compound **18**, as reported and executed by Zhang et al. (2021) *.*

The fusion of **theophylline**, **ITI-214**, and **SCH-51866** backbones, followed by SAR exploration at N-7, C-2, and C-3 positions of the pyrazolo-pyrimidone core, resulted in the development of a series of pyrazolo-pyrimidone derivatives. Compound **18 (2j)** emerged as a potent PDE1 inhibitor (PDE1B IC_50_: 21 nM) with good metabolic stability in rat liver microsomes (T_1/2_: 28.5 min). Detailed binding interaction analysis of **ITI-214** and **SCH-51866** with PDE1 revealed that the N_7_-bound nitrogen in the pyrazolo-pyrimidone core acts as an H-bond acceptor, forming an H-bond with Y222 in the PDE1 catalytic site. As the substitution at this position was bulky, it was hypothesized that replacing it with smaller hydrogen bond acceptor groups could enhance inhibitory activity. Following this hypothesis, introducing a cyclopropylmethyl group at the N_7_-position resulted in exceptional PDE1 inhibitory activity. In contrast, larger substitutions, such as the 2-oxo-2-phenylethyl group at the same position, yielded significantly reduced PDE1 inhibitory activity, indicating a preference for smaller groups at this position. However, the compound with a cyclopropylmethyl group at the N_7_-position demonstrated poor microsomal stability, prompting structural optimization at the N_2_-position to improve metabolic stability. It was anticipated that substituting saturated heterocycles such as oxetane at the N_2_-position could modify metabolic pathways and enhance metabolic stability. Furthermore, substituting 4-(methylsulfonyl) benzyl at the N_2_-position increased PDE1 inhibitory activity and improved microsomal stability. Moreover, the SAR investigation indicated that aerobic metabolism of the phenylamino ring at the C_3_-position contributes to structural instability. Replacing this group with (4-fluorophenyl) amino moiety, along with optimal substitutions at the N-7, C-2, and C-3 positions discussed above, resulted in Compound **18**, which exhibited the most potent (IC_50_: 21 nM) and selective (over 480-fold *versus* PDE8) PDE1 inhibitor among the synthesized derivatives, along with good metabolic stability in rat liver microsomes (T_1/2_: 28.5 min). Structural optimization revealed that incorporating small hydrophobic groups, such as cyclopropylmethyl, at the N_7_-position enhanced potency, while a sulfonyl group at the N_2_-position and (4-fluorophenyl) amino at the C_3_-position improved metabolic stability (Figures 7A,B). Binding interaction analysis of Compound **18** with PDE1 revealed that the pyrazolo-pyrimidone core of Compound **18** positioned itself between F424 and L388, establishing H-bonds with Q421. The 4-substituted benzyl chain formed a hydrophobic bond with F424 and F427, while the C3-(4-fluorophenyl) amino side chain occupied a lipophilic pocket made up of F392, L409, and V417 (Figures 7A,B). The synthetic accessibility score and MW of **18** were 3.73 and 497.54 g/mol, respectively, which lie between those of **ITI-214** and **DNS-0056**, indicating a moderate synthesis approach for Compound **18** compared to **ITI-214** and **DNS-0056** (Daina et al., 2017). The synthesis of **18** followed the same method used for **ITI-214** with some modifications, starting with the protection of Compound **19** with a PMB group and subsequent chlorine displacement with hydrazine hydrate to obtain Compound **20**. Phenyl isothiocyanate was then treated with Compound **20** and afforded the pyrazolo-pyrimidinone ring-containing Compound **21**, which was treated with a substituted benzyl halide to afford Compound **22**. Finally, the PMB group was eliminated from Compound **22**, yielding an intermediate which, after reaction with halides in the presence of K_2_CO_3_, produced Compound **18** (Scheme 3) (Zhang et al., 2021).

#### 3.1.4 Dihydrobenzofuran derivatives

**FIGURE 8:**
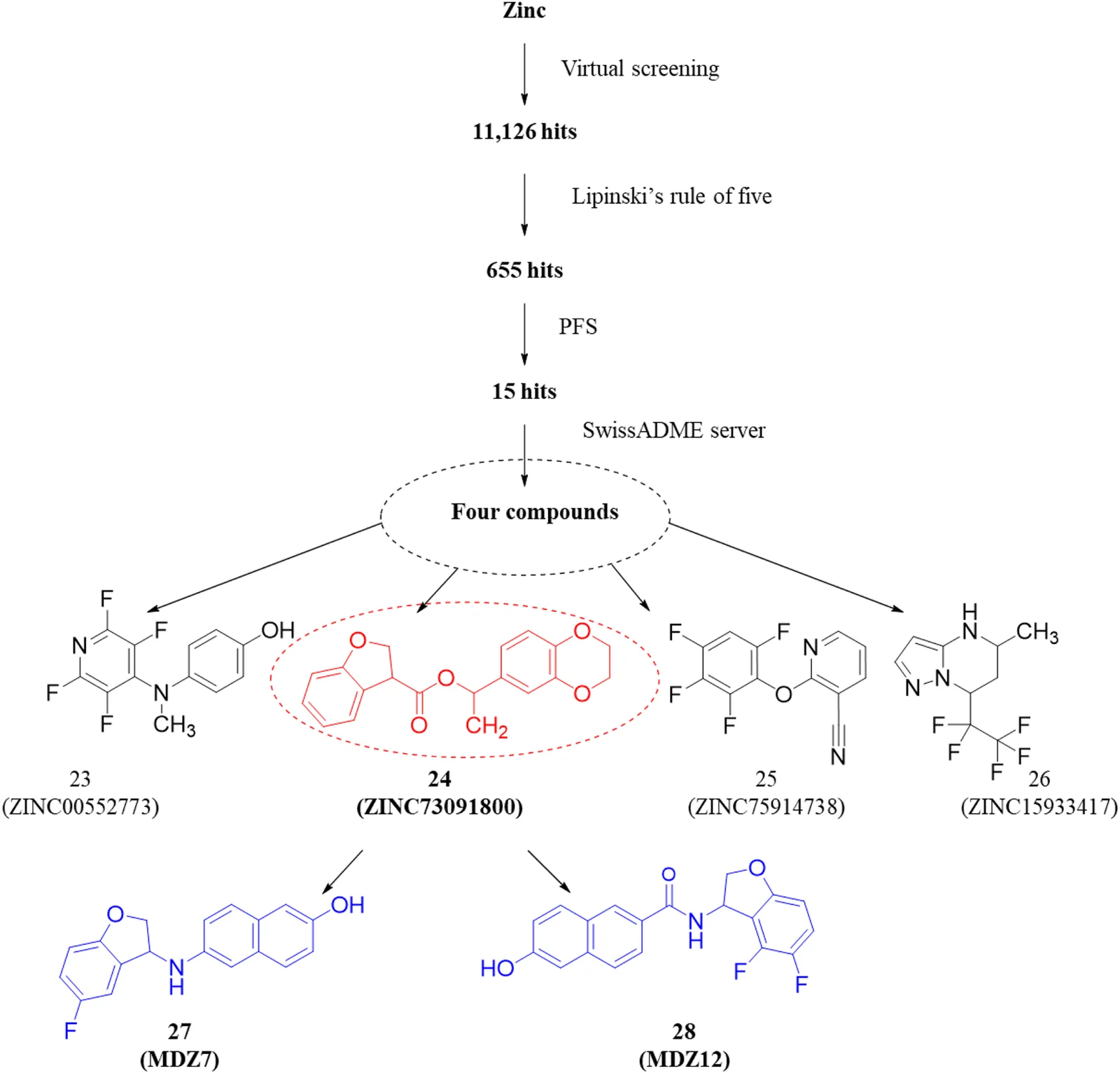
Discovery of new dihydrobenzofuran scaffold containing PDE1 inhibitors. Pharmacophore screening of the ZINC database yielded 11,126 hits, which were narrowed to 2 candidates (compounds **27** and **28**) using an *in silico* approach.

**FIGURE 9:**
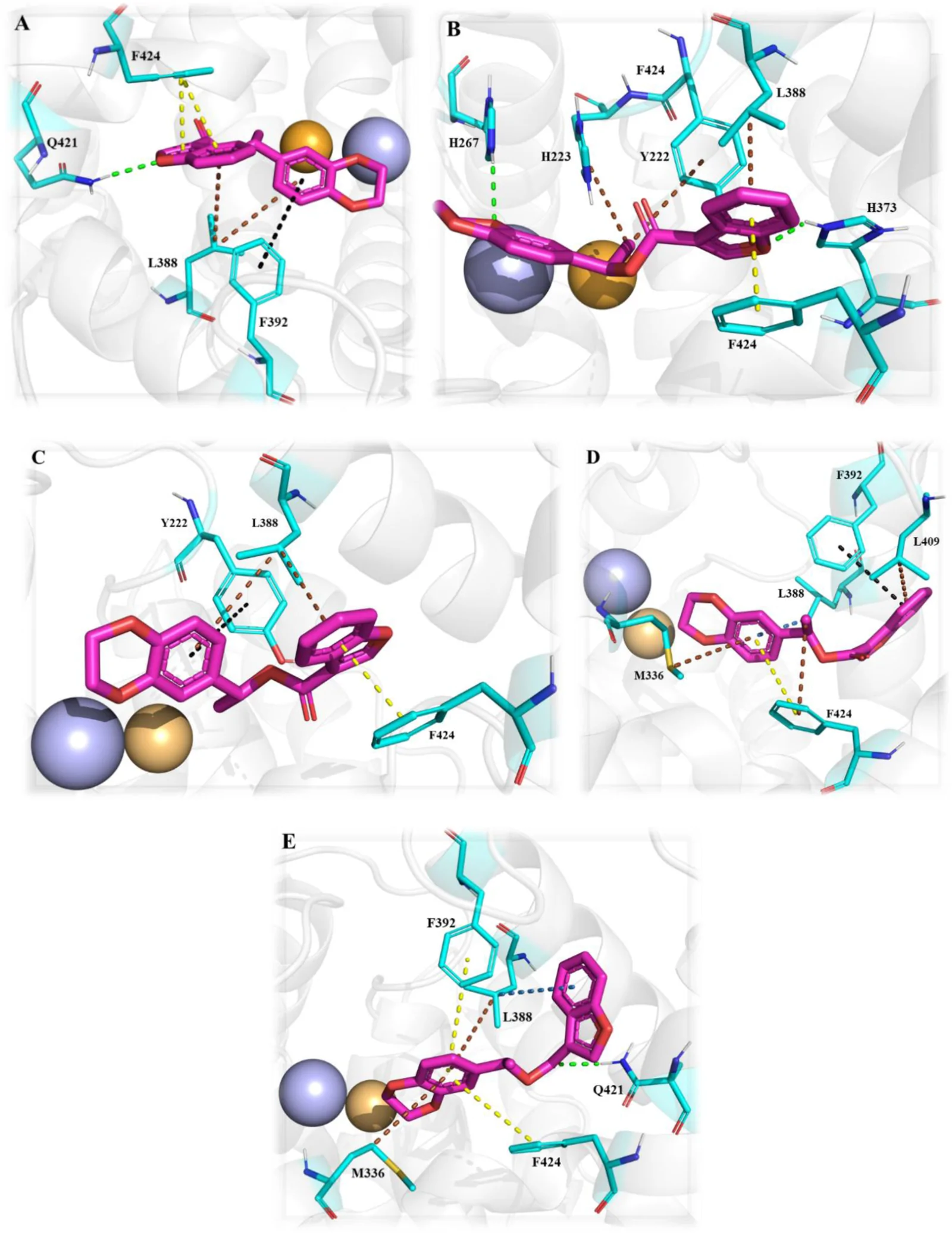
The multi-conformational interaction analysis of Compound **24** with five PDE1B structures. **(A)** PDB: 4npv. **(B)** PDB: 4npw. **(C)** PDB: 5b25. **(D)** PDB: 5uoy. **(E)** PDB: 5up0. The comparison demonstrates that ligand flexibility and pocket plasticity contribute to conserved and divergent interaction motifs across different PDE1B conformations.

Recent computational drug design efforts have discovered novel 2,3-dihydrobenzofuran scaffold-containing molecules, **27** (MDZ7) and **28** (MDZ12), as potential PDE1B inhibitors, exhibiting notable binding affinities of −9.6 and −9.5, respectively, with the protein. A pharmacophore model from the 5UOY–16j complex was used to screen the ZINC database, yielding 11,126 hits, which were narrowed down to 4 candidates, namely, compounds **23**, **24**, **25**, and **26** (ZINC00552773, ZINC75914738, ZINC73091800, and ZINC15933417) using Lipinski’s rule, the PFS filter, and the SwissADME server (Figure 8). These hits were docked with five PDE1B crystal structures (PDB IDs: 5UOY, 5UP0, 4NPW, 4NPV, and 5B25) to analyze protein–ligand interactions. In the context of these co-crystal structures, Compound **24** consistently engaged key conserved hydrophobic residues (M336, L388, F392, and F424) and formed hydrogen bonds with Q421, while additional interactions with H223, Y222, I371, H373, I428, P408, L409, M389, and V417 contributed to specificity (Figures 9A–E).

Compound **24** exhibited various hydrophobic interactions with them, and its planar ring occupation within the active site suggested its potential as a lead molecule for further structural modification. The modifications focused on the 1,4-dioxin moiety and the isopropyl acetate bond as the 2,3-dihydrobenzofuran core and benzyl ring of the 2,3-dihydrobenzodioxin groups were crucial for preserving hydrophobic contacts with the P-clamp residues. Initially, based on the understanding that amine or amide groups could enhance hydrophobic and hydrophilic interactions, the isopropyl acetate linkage was replaced with these groups. Subsequently, to increase hydrogen bond interactions with Q421 and π-stacking interactions with F424 in PDE1B, the 1,4-dioxin group was replaced with phenol or phenoxyamine groups. Additionally, to enhance halogen interaction opportunities, a fluorine group was incorporated at the ortho site of the benzyl group in 2,3-dihydrobenzofuran. Interestingly, through these structural optimizations, compounds **27** and **28** were obtained as the highest-affinity compounds. In compounds **27** and **28**, the 1,4-dioxin group was replaced with phenol, the isopropyl acetate linkage was replaced with amine and amide, respectively, and the benzyl ring of the 2,3-dihydrobenzodioxin groups was replaced with 6-fluoro and 5,6-difluoro substitutions, respectively. This suggests that the phenol ring is most favorable due to its significant hydrophilic and hydrophobic interactions, while the amine and amide groups, being polar, offer hydrogen bonding interactions. Additionally, the fluorine groups facilitate halogen interactions with the active site residues. Therefore, compounds **27** and **28** exhibited the most significant affinity for PDE1B among all the compounds, designating them as potential candidates for synthesis and preclinical assessment to evaluate their PDE1B inhibition and antipsychotic properties (Al-Nema et al., 2023). However, studies related to this have yet to be published.

#### 3.1.5 4-Aminoquinazoline and 4-indanylquinazoline derivatives

**FIGURE 10:**
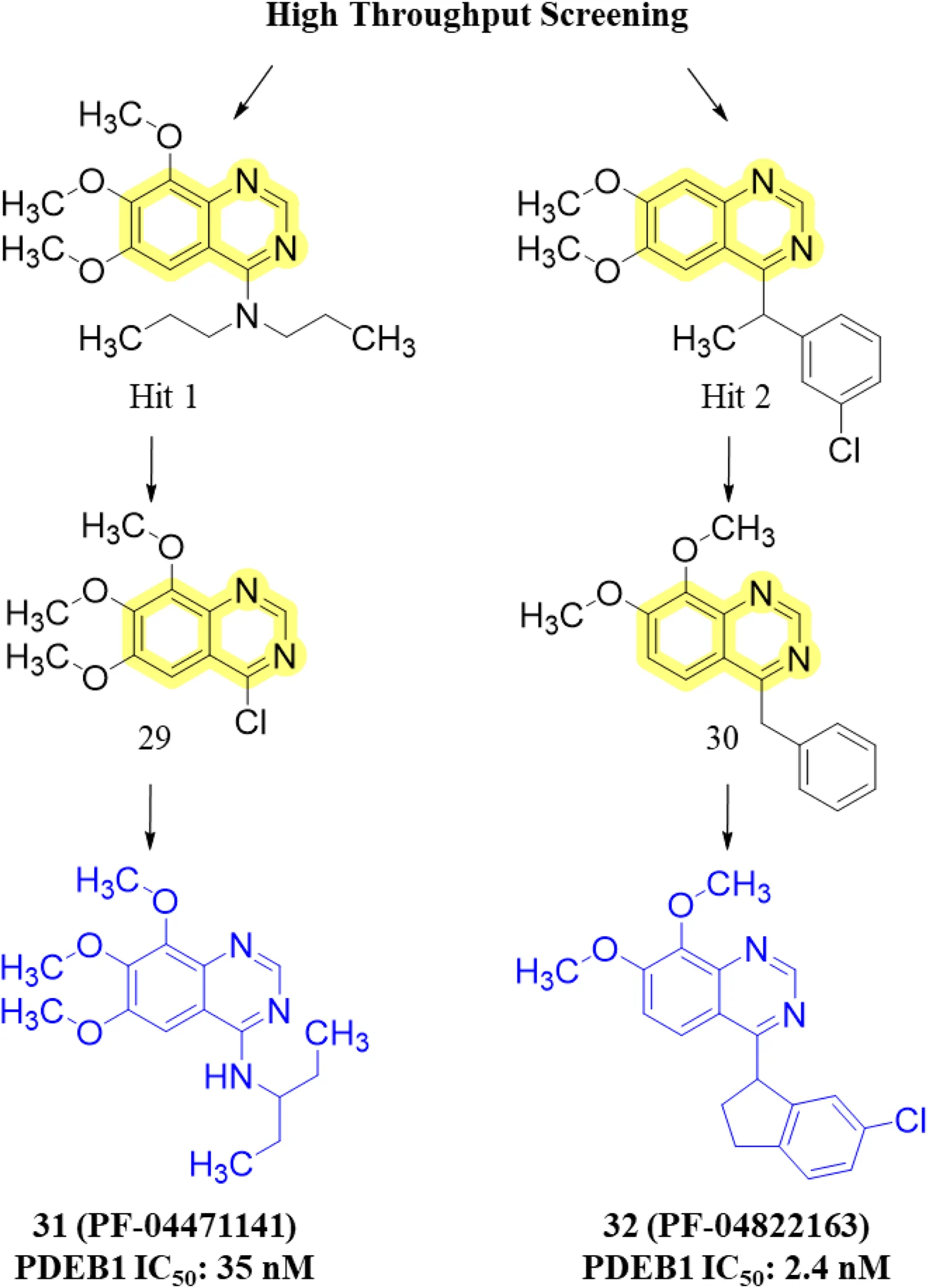
Systematic optimization of the quinazoline core identified Compound **31** as an active PDE1B inhibitor. Further indane incorporation and repositioning of the methoxy group led to Compound **32**, a highly potent PDE1B inhibitor.

**FIGURE 11:**
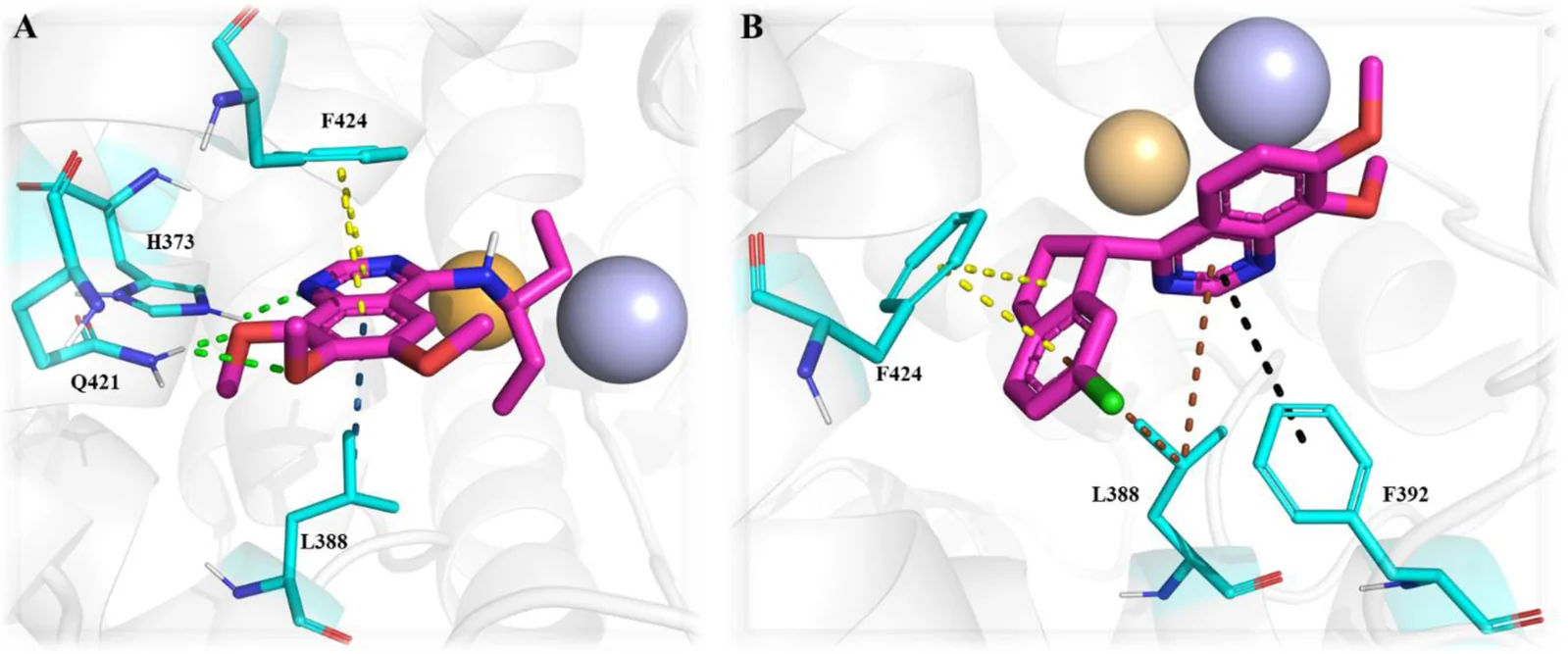
**(A)** Binding interactions of Compound **31** with PDE1B (PDB: 4npv), showing the quinazoline scaffold orientation within the active site, bifurcated hydrogen bonds of 7- and 8-methoxy groups with Q421, hydrogen bond of N-1 with H373, and π–σ/π–π interactions with L388 and F424. **(B)** Binding interactions of Compound **32** with PDE1B, showing a similar ligand orientation to Compound **31**, with the chlorine atom occupying the hydrophobic pocket formed by L388 and F392, contributing to its binding profile.

Another important class of PDE1B inhibitors, containing the quinazoline nucleus, was discovered by Nadur *et al.* Initial high-throughput screening (HTS) led to the discovery of compounds **31 (PF-04471141)** and **32 (PF-04822163)** as potent, brain-penetrating, and selective PDE1B inhibitors. Based on their previous work on PDE10A, it was hypothesized that the 6, 7-dimethoxyquinazoline group of **hits 1** and **2** can form a bidentate hydrogen bonding interaction between a PDE-invariant glutamine residue of PDE1B. Therefore, two different approaches focused on modifying the quinazoline structure. In the first approach, a series of 4-chloro-6,7,8-trimethoxyquinazoline (**29**) derivatives were synthesized and coupled with a variety of amines, which indicated that 7,8-dimethoxy functionalization on the quinazoline ring enhanced PDE1B inhibition, while the presence of a third 6-methoxy group was neither beneficial nor detrimental. The most potent compound from this series, Compound **31**, exhibited an IC_50_ value of 35 nM against PDE1B. In contrast, the second approach explored replacing the quinazoline nucleus with alternative templates such as phthalazine, cinnoline, and quinoline. Although these templates showed activity against PDE10A, they were ineffective against PDE1B, highlighting the essential role of the quinazoline core in PDE1B inhibition. Furthermore, the synthesis and SAR exploration of another series, 4-benzyl-7,8-dimethoxyquinazoline (**30**) derivatives, indicated that removing the chlorine and benzylic methyl group from the quinazoline structure reduced binding affinity toward PDE1B, whereas structural rigidification enhanced potency. Notably, transposing the methoxy group from the 6- to the 8-position on the quinazoline ring significantly increased potency, while replacing the chlorine atom with bromine or fluorine in the indane group decreased potency, likely due to size constraints within the binding pocket. This structural optimization led to the identification of Compound **32**, a highly potent indane derivative with improved PDE1B inhibitory activity (PDE1B IC_50_: 2.4 nM) (Figure 10). The quinazoline scaffold plays a critical role in PDE1B inhibition. In Compound **31**, the 7- and 8-methoxy groups form a bifurcated hydrogen bond with the side chain nitrogen of Q421, while the N_1_ atom engages His373. Hydrophobic interactions, including π–σ and π–π stacking with L388 and F424, further enhance potency (Figure 11A). Compound 32 binds similarly, with the chlorine atom occupying the hydrophobic pocket defined by L388 and F392, which may account for its slightly lower potency compared to Compound **31** (Figure 11B).

**SCHEME 4:**
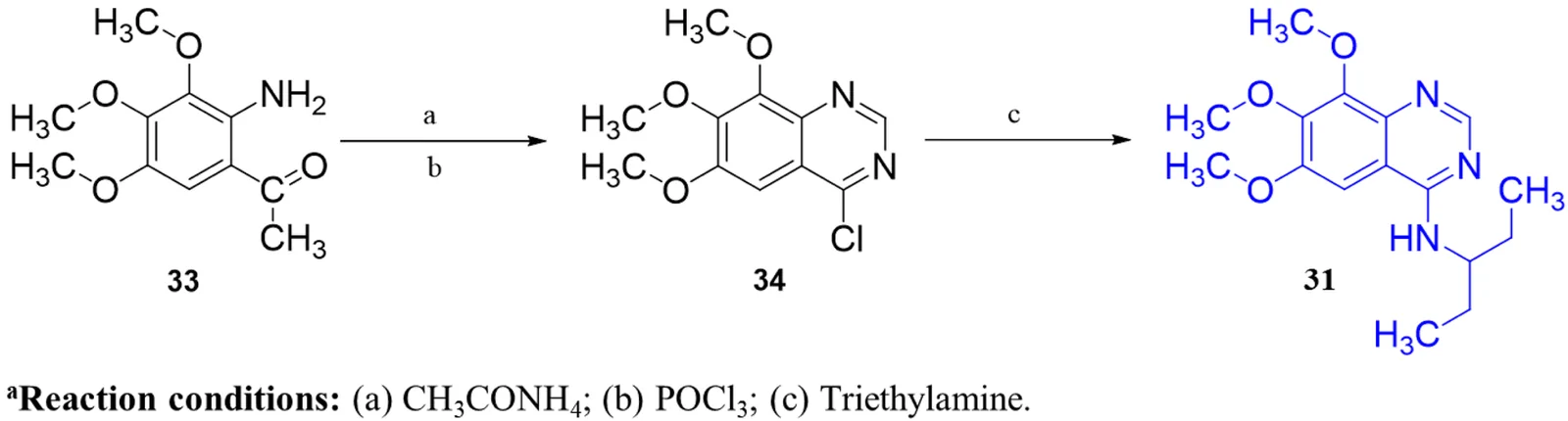
Synthesis of Compound **31**, as reported and employed by Humphrey (2014) *.*

**SCHEME 5:**
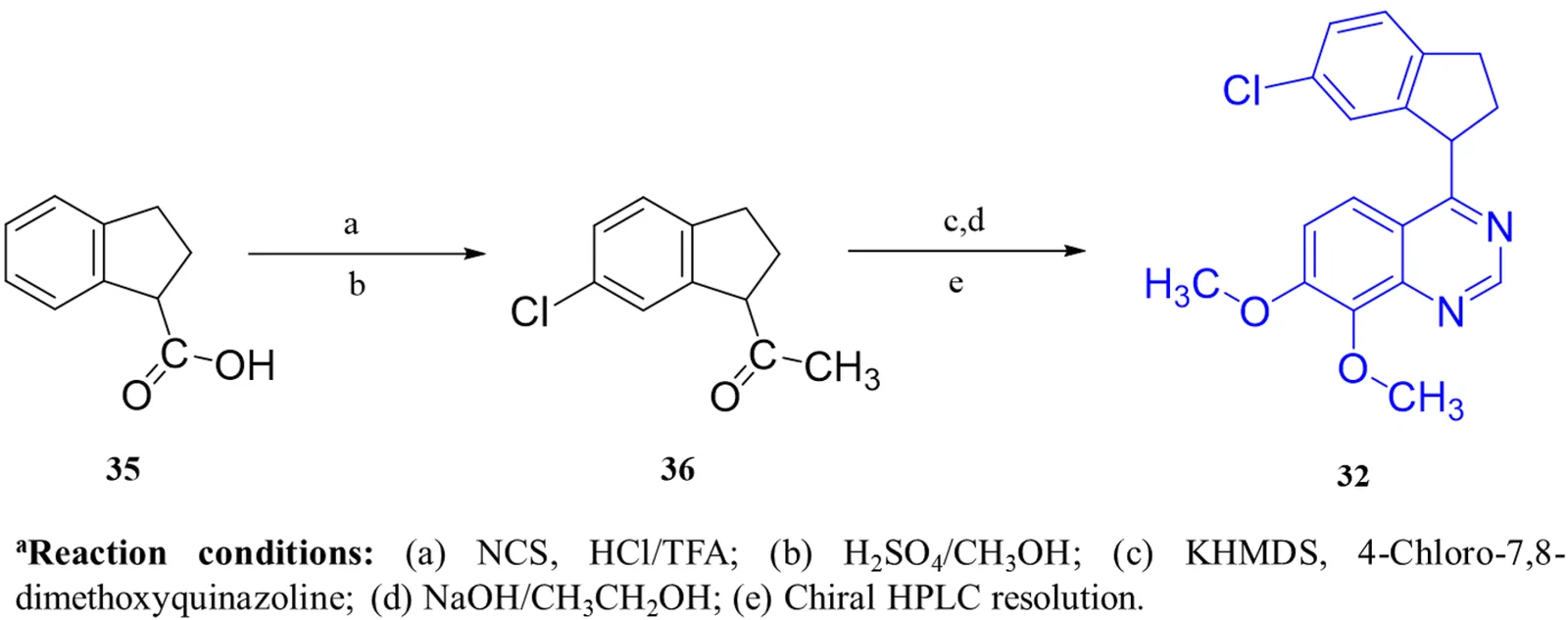
Synthesis of Compound **32**, as reported and executed by Humphrey (2014) *.*

The synthetic accessibility score and MW of Compound **31** were 2.61 and 291.35 g/mol, respectively, marking the lowest values among the molecules discussed above, thereby indicating the most facile synthetic route. In addition, Compound **31** exhibits favorable pharmacokinetic properties, including high GI absorption and significant brain penetration, with no violations of Lipinski’s rules, which also supports its commercial availability from Sigma-Aldrich (Daina et al., 2017). The synthesis of Compound **31** began with the formation of intermediate **34** through the reaction of Compound **33** with ammonium acetate, followed by subsequent treatment with phosphoryl chloride. Intermediate **34** was then refluxed with triethylamine to afford the final product, Compound **31** (Scheme 4). Conversely, Compound **32**, with a synthetic accessibility score of 3.23 and MW of 340.80 g/mol, higher than that of Compound **31**, was synthesized *via* a multi-step reaction (Daina et al., 2017). The synthesis initiated with the direct chlorination of Compound **35**, yielding chloroindane methyl ester **36**, which, after chiral resolution using HPLC, afforded Compound **32** (Scheme 5) (Humphrey, 2014).

## 4 PDE10

PDE10 is a dual-substrate hydrolase that acts on cAMP and cGMP. It has a single subtype, PDE10A, which hydrolyzes cAMP (Km: 0.05 μM) and cGMP (Km: 3 μM) into their inactive forms (Zagorska et al., 2018; Amin et al., 2021). The active site of PDE10A bound to cAMP and cGMP was first reported in 2007, consisting of 340 amino acids (Wang et al., 2007). PDE10A is most highly expressed in the MSNs of the striatum, alongside PDE1B (Xie et al., 2006; Nishi et al., 2008). PDE10A is pivotal in regulating the cAMP/PKA/CREB signaling pathways downstream of dopamine and glutamate receptors, which are notably dysregulated in the brains of individuals with schizophrenia (Threlfell and West, 2013; Calabresi et al., 2014). PDE10A inhibition has been shown to activate the cAMP/PKA cascade in the striatopallidal (indirect) pathway, resulting in the inhibition of thalamocortical circuits, thereby mimicking the effects of a D2-antagonist. Conversely, inhibition of PDE10A also activates cAMP/PKA signaling in the striatonigral (direct) pathway, resulting in the activation of thalamocortical circuits and replicating the effects of a D1-agonist. The equilibrium between these two circuits is crucial for the regulation of motor and executive functions (Figure 2) (Kehler and Nielsen, 2011). Thus, targeting PDE10A holds promise as a therapeutic strategy for schizophrenia.

### 4.1 Insight into PDE10 inhibitors

**FIGURE 12:**
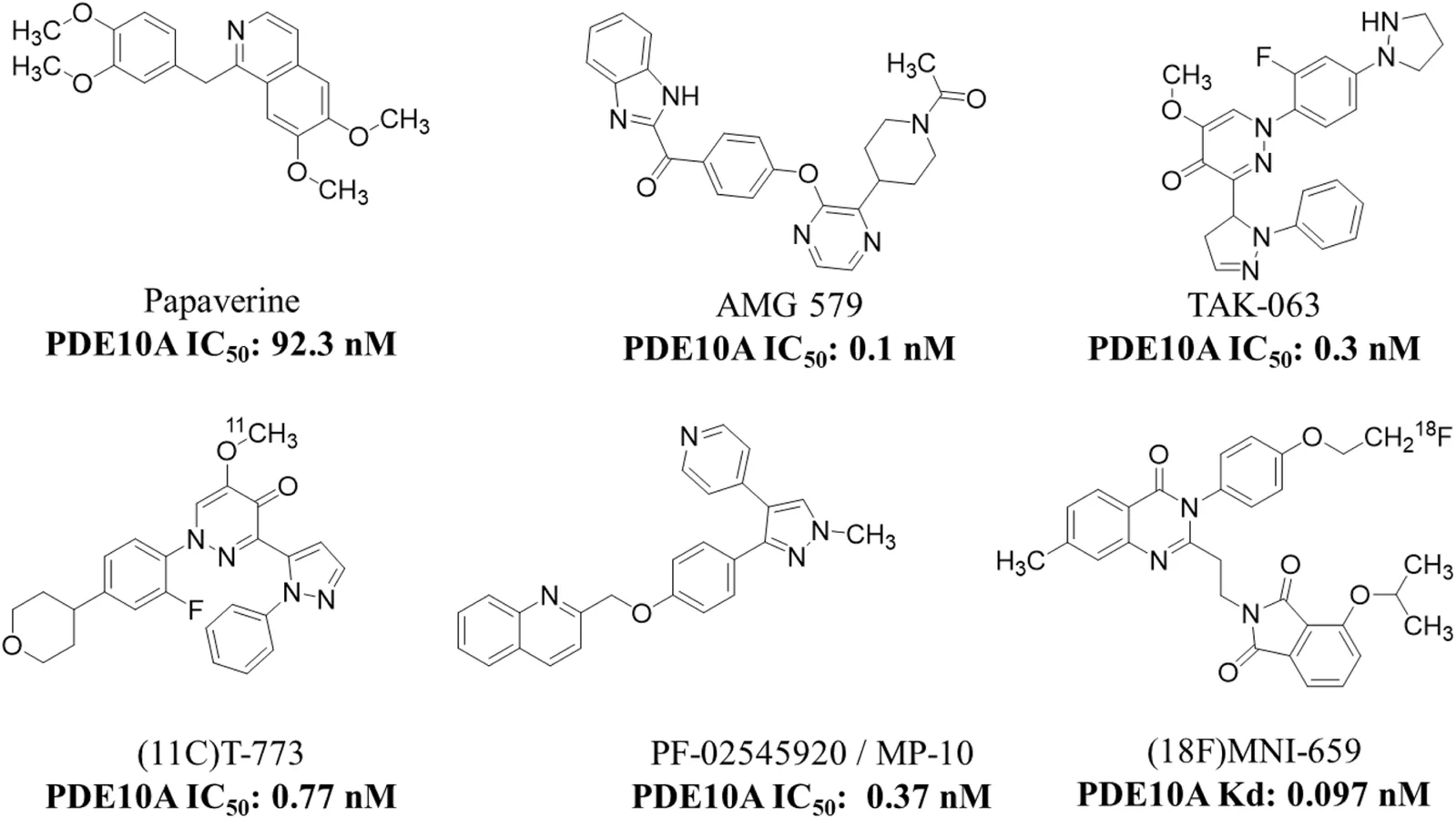
Chemical structure of PDE10A inhibitors evaluated in preclinical and clinical studies.

PDE10A has emerged as a promising druggable target, and its inhibitors are being explored for the treatment of schizophrenia. Over the last 10 years, substantial efforts have been directed toward the discovery of PDE10A inhibitors with diverse chemical scaffolds (Chappie et al., 2012; Zagórska, 2020). Studies, such as Reneerkens et al. (2013), have shown that PDE10A inhibition can ameliorate scopolamine-induced object memory deficits in animal models, highlighting its potential role in addressing schizophrenia. To date, various PDE10A inhibitors, including papaverine, AMG-579, TAK-063, (11C) T-773, MP-10, and (18F) MNI-659, have been developed and evaluated in both preclinical and clinical studies (Figure 12) (Świerczek et al., 2019). However, many of these compounds exhibit limitations such as weak or non-selective PDE10 inhibition. This emphasizes the ongoing necessity for the development of potent and selective PDE10A inhibitors. Some of these efforts are described below.

#### 4.1.1 Imidazo[1,2-a] pyrazine derivatives

**FIGURE 13:**
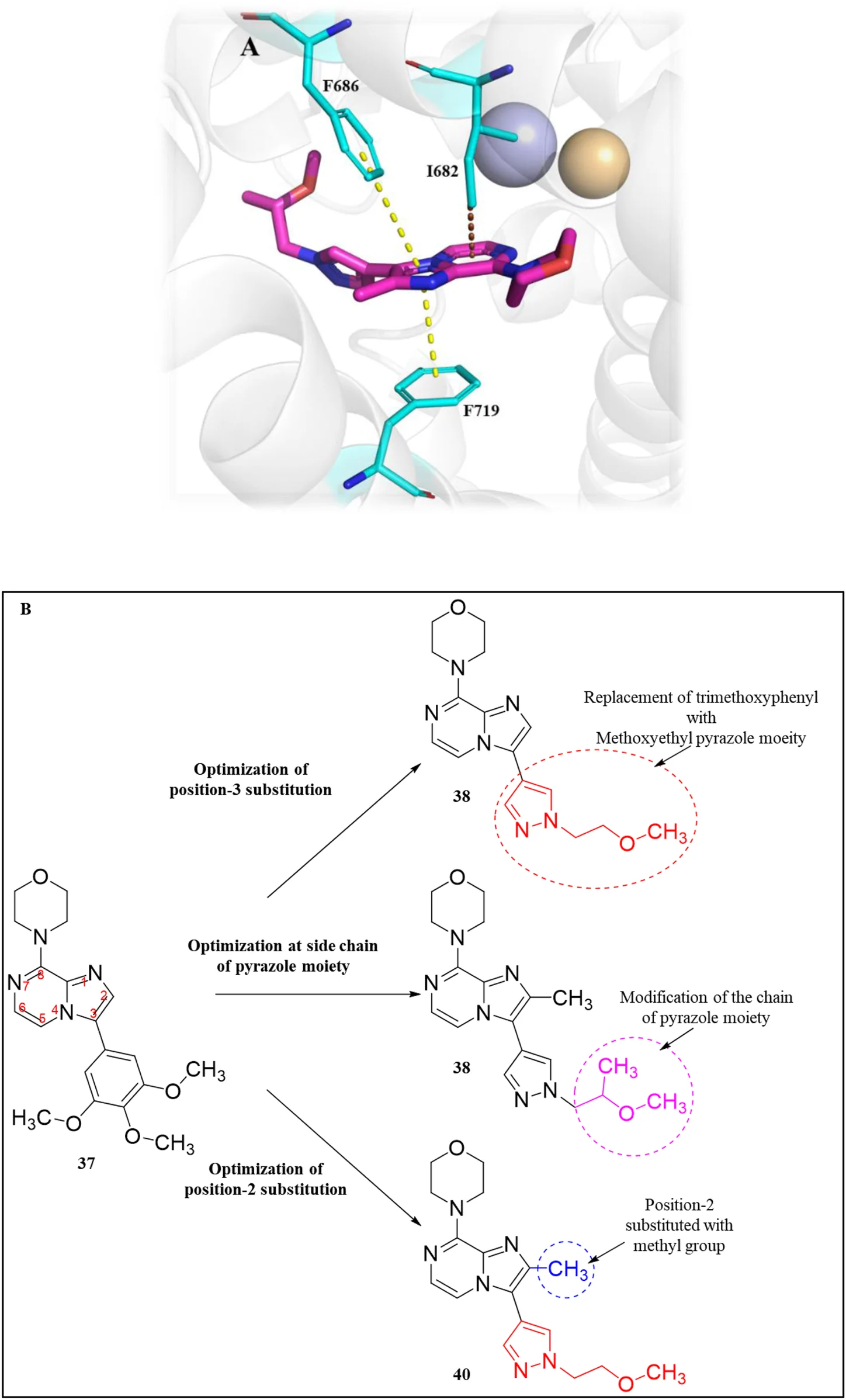
**(A)** Binding interaction of Compound **39** with PDE10A (PDB: 4bbx), exhibiting the imidazo[1,2-a] pyrazine scaffold orientation within the active site, morpholine ring occupancy of the Q1 pocket, and hydrophobic interactions of the pyrazole group with F689, I682, and F719. **(B)** Structural optimization leading to Compound **40**, highlighting modifications at the 2-position of the imidazo[1,2-a] pyrazine scaffold, which results in a potent and selective PDE10A inhibitor with improved *in vivo* efficacy and PK properties.

**SCHEME 6:**
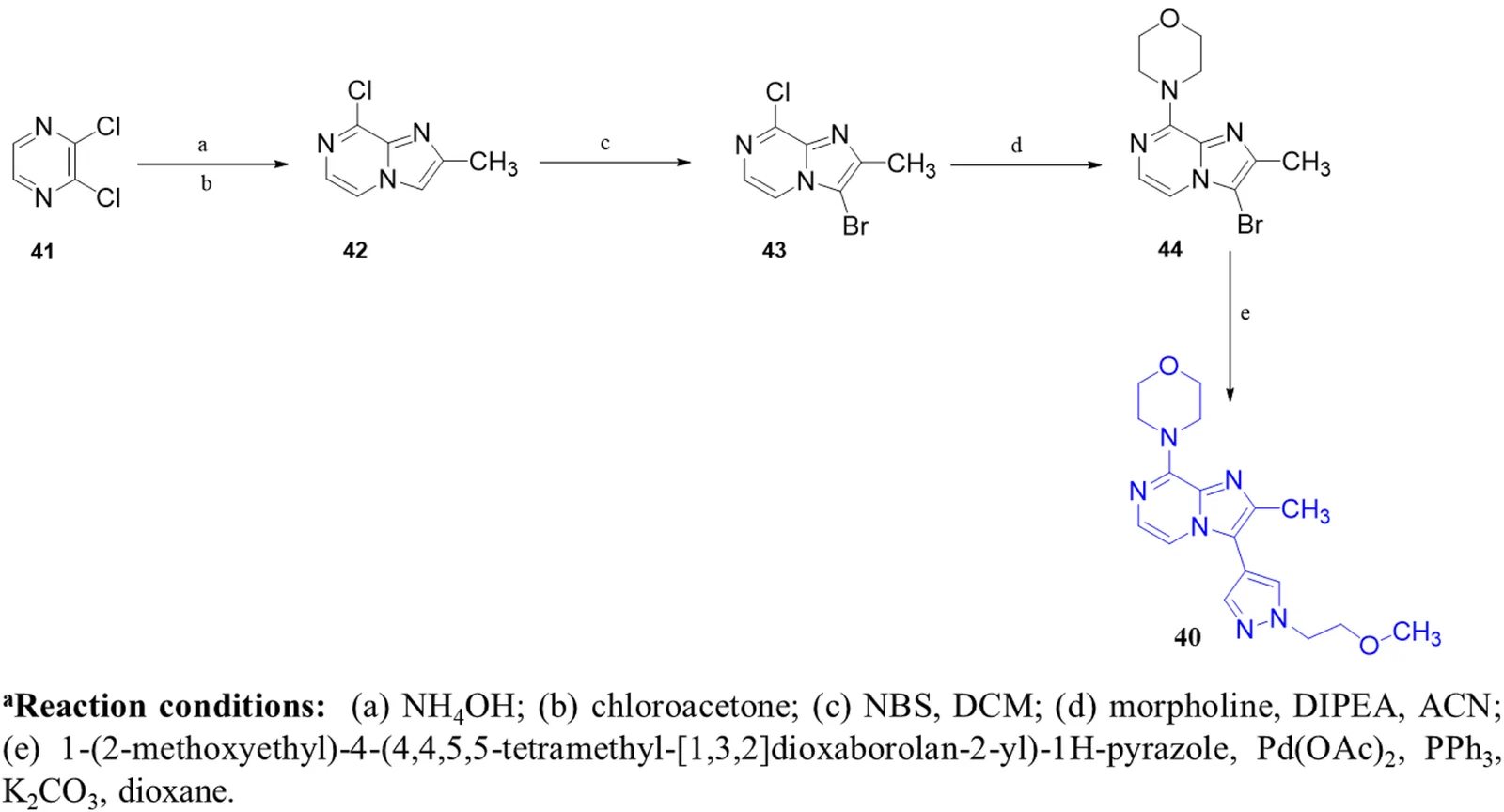
Synthesis of Compound **40**, as reported and employed by Bartolomé-Nebreda et al. (2014)

An HTS campaign and subsequent structural optimization of Compound **37**, an initially identified PDE10A inhibitor, led to the discovery of imidazo[1,2-a] pyrazine-containing Compound **40**, a potent and selective PDE10A inhibitor exhibiting promising *in vivo* efficacy across various rodent behavioral models of schizophrenia, along with favorable pharmacokinetic properties in rats. Compound **37**, though effective, displayed limited selectivity toward PDE1, and this may be attributed to the presence of the trimethoxyphenyl moiety in Compound **37.** As a result, the optimization process focused on substituting the trimethoxyphenyl group with various functional groups. Compound **38**, containing a (methoxyethyl)pyrazole group, emerged as a successful substitution, enhancing selectivity (pIC_50_ for other PDEs <5) while reducing potency toward PDE10A (pIC_50_: 6.0), related to Compound **37**. In order to enhance PDE10A potency, modifications were introduced to the pyrazole moiety, leading to the identification of Compound **39**, which exhibited improved PDE10A inhibitory activity. To gain insight into the critical interaction characteristics of the imidazo[1,2-a] pyrazine series, crystallographic studies of the Compound **39** complex with PDE10A were analyzed. The binding interactions revealed a characteristic mode, where the morpholine ring expanded into the Q1 pocket, while the R-group on the pyrazole moiety projected into the solvent-exposed region, establishing hydrophobic interactions with F689, I682, and F719 (Figure 13A). To further enhance PDE10A potency, diverse substitutions were explored at the 2-position of the bicyclic imidazo[1,2-a] pyrazine scaffold. This effort resulted in the identification of Compound **40**, which exhibited improved PDE10A inhibitory activity compared with other compounds (Figure 13B). Subsequent evaluation of these compounds for *in vivo* PDE10A inhibition using an apomorphine-induced stereotypy model in rats revealed that Compound **40** exhibited exceptional *in vitro* and *in vivo* potency, along with favorable microsomal stability. Given the encouraging results in the apomorphine-induced stereotypy model, Compound **40** was further tested for antipsychotic activity in a phencyclidine (PCP)-induced hyperlocomotion model in rats. Compound **40** effectively reversed PCP-induced hyperlocomotion. Additionally, protein binding in the rat brain was validated by the displacement of the selective PDE10A ligand [3H]MP-10 in the striatum. Finally, pharmacokinetic analysis revealed that Compound **40** possessed a well-balanced profile among all the derivatives. The synthetic accessibility score and MW of Compound **40** were 3.46 and 342.40 g/mol, respectively, indicating a relatively more complex synthetic route compared to other compounds (Daina et al., 2017). The synthesis began with commercially available starting material Compound **41**, which was treated with ammonium hydroxide, followed by condensation with chloroacetone to yield intermediate **42.** Selective bromination at position 3 was achieved using N-bromosuccinimide (NBS), resulting in intermediate **43**. Subsequent reaction of intermediate **43** with morpholine led to the formation of intermediate **44.** Finally, Compound **40** was synthesized *via* a Suzuki−Miyaura palladium-catalyzed cross-coupling reaction between intermediate **44** and 1-(2-methoxyethyl)-4-(4,4,5,5-tetramethyl[1,3,2]dioxaborolan-2-yl)-1H-pyrazole, completing the desired structure (Scheme 6) (Bartolomé-Nebreda et al., 2014).

#### 4.1.2 Dihydro-1H-benzo[d]imidazo[1,2-a]benzimidazole derivatives

**FIGURE 14:**
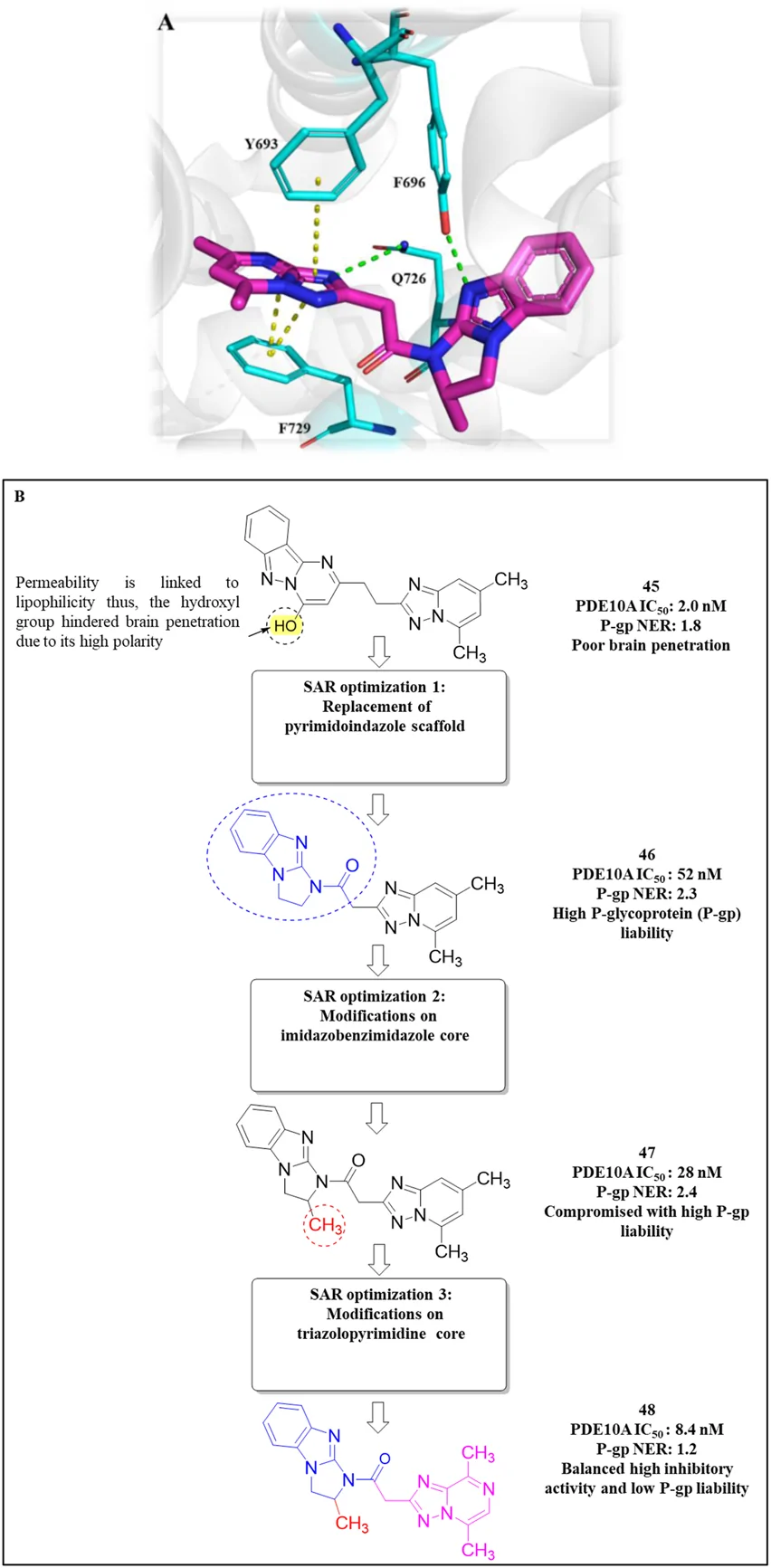
**(A)** Binding interaction of Compound **47** with PDE10A (PDB: 6ke0), showing the dihydro-imidazo[1,2-a]benzimidazole scaffold orientation within the active site and hydrogen bonding of the nitrogen at the 9-position with Y693. **(B)** Structural optimization leading to Compound **48**, illustrating modifications of the triazolopyrimidine/triazolopyrazine moieties to reduce P-gp efflux while maintaining PDE10A inhibitory activity and favorable PK properties.

**SCHEME 7:**
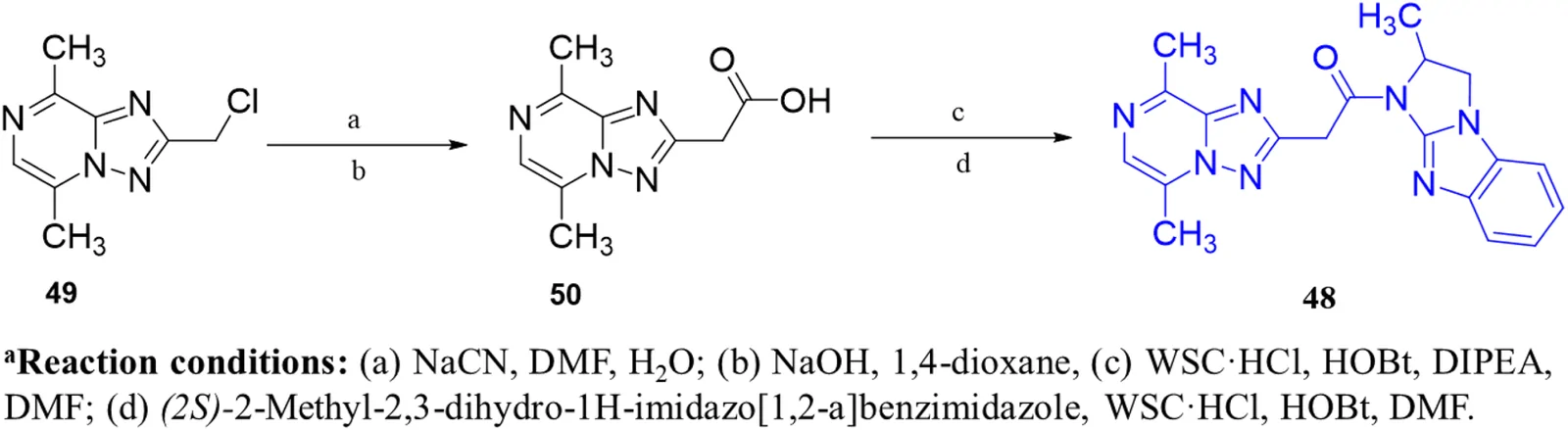
Synthesis of Compound **48**, as reported by Chino et al. (2019) *.*

The development of dihydro-imidazo benzimidazole-based PDE10A inhibitors was reported by Chino et al. (2019), focusing on overcoming the blood–brain barrier permeability issue identified in the previous molecule. Initially, Compound **45** demonstrated strong inhibitory activity against PDE10A, but poor brain penetration (Pint: 3.2 × 10^−6^ cm/s) limited its therapeutic potential. To address this, Compound **47** was developed through structural modifications, significantly reducing P-glycoprotein (P-gp) efflux liability, enhancing PDE10A inhibition, and improving brain penetration, thereby reversing MK-801-induced working memory deficits in preclinical models. SAR studies focused on modifying the pyrimidoindazole core. Various replacements, including aminoquinoline, quinoxaline, and quinazoline derivatives, were explored, in which the benzimidazole-based **46** demonstrated superior potency and permeability. Further refinement of the scaffold resulted in the identification of (S)-2-methyl-2,3-dihydro-1H-benzo[d]imidazo[1,2-a] imidazole containing Compound **47** (Figures 14A,B). The X-ray co-crystal structure of Compound **47** in complex with PDE10A enzyme demonstrated that the nitrogen atom at the 9-position of the imidazo[1,2-a]benzimidazole ring interacts with Y693. Additionally, the nitrogen group of the imidazo[1,2-a]benzimidazole ring engages in hydrogen bonding with Q726, which likely contributes to its enhanced potency. While Compound 47 showed improved the PDE10A inhibitory activity, it continued to exhibit significant P-gp liability (Figures 14A,B). In order to overcome this, efforts focused on modifying the nitrogen arrangement on the triazolopyrimidine ring. By varying the number and position of nitrogen atoms, particularly at key positions, researchers aimed to reduce P-gp interaction. Substitution with imidazopyrimidine and triazolopyridine moieties successfully lowered P-gp efflux but resulted in a marked decrease in PDE10A inhibitory potency, highlighting the importance of the 1- and 4-nitrogen atoms in maintaining both high inhibitory activity and minimal P-gp susceptibility. Finally, the triazolopyrazine-substituted Compound **48 (6d)** exhibited balanced PDE10A inhibition and low P-gp liability, along with favorable pharmacokinetics (PK). It also showed minimal CYP enzyme inhibition, highlighting its therapeutic potential. The MW of Compound **48** was 361.40 g/mol, with a synthetic accessibility score of 3.74, slightly higher but still within a drug-likeness range, with a bioavailability score of 0.55. Synthesis began with the cyanation of chloromethyl-triazolopyrazine **49**, followed by hydrolysis with NaOH to form intermediate **50**. Subsequent treatment with WSC·HCl and (2S)-2-methyl-2,3-dihydro-1H-imidazo[1,2-a]benzimidazole yielded Compound **48** (Scheme 7) (Chino et al., 2019).

#### 4.1.3 Pyrimido[1,2-b]indazole derivatives

**FIGURE 15:**
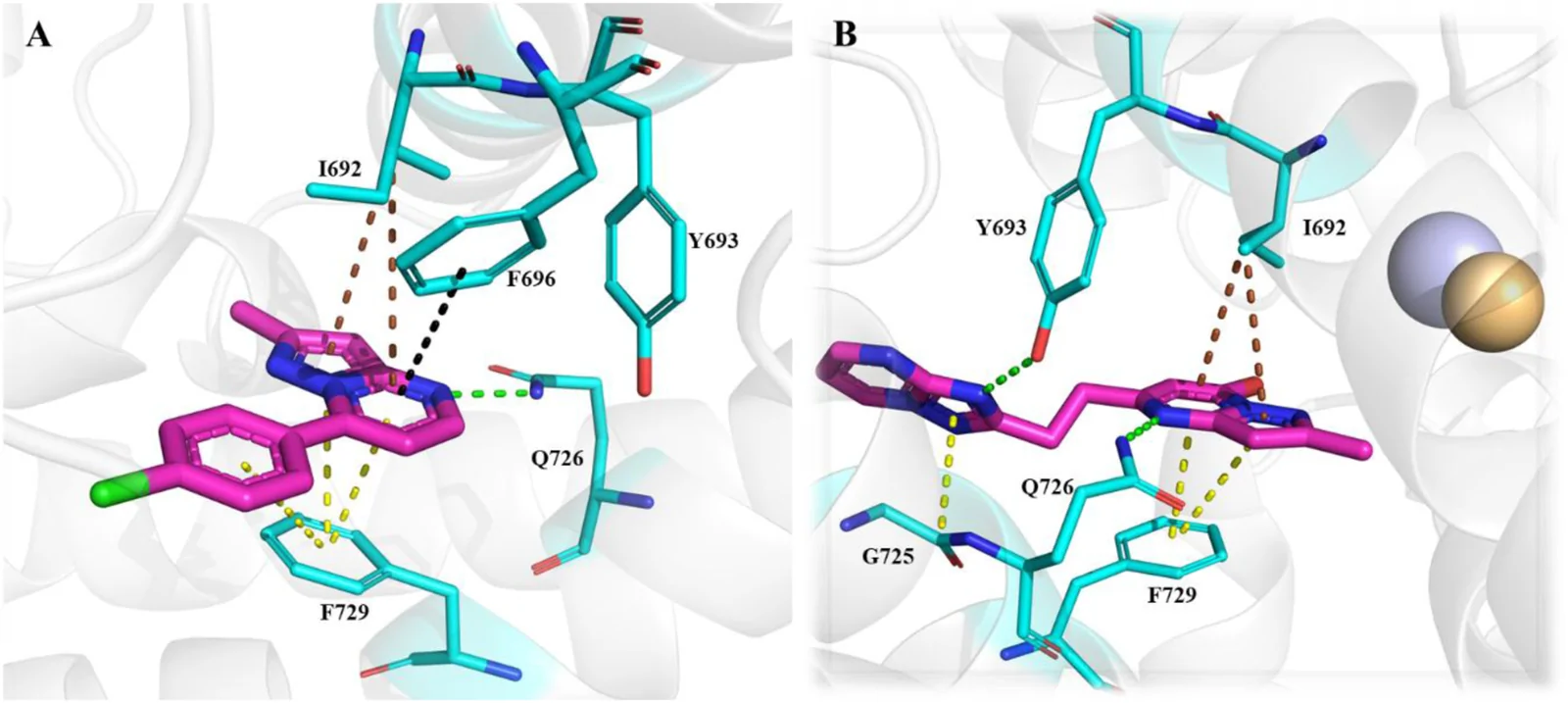
**(A)** Binding interaction of Compound **51** with PDE10A (PDB: 5xuj), displaying the pyrimidoindazole scaffold orientation within the active site, hydrogen bonds of the pyrazolopyrimidine ring with Q726, and hydrophobic interactions with the P-clamp. **(B)** Co-crystal structure of Compound **53** binding with PDE10A (PDB: 5xui), exhibiting an additional interaction with G725.

**FIGURE 16:**
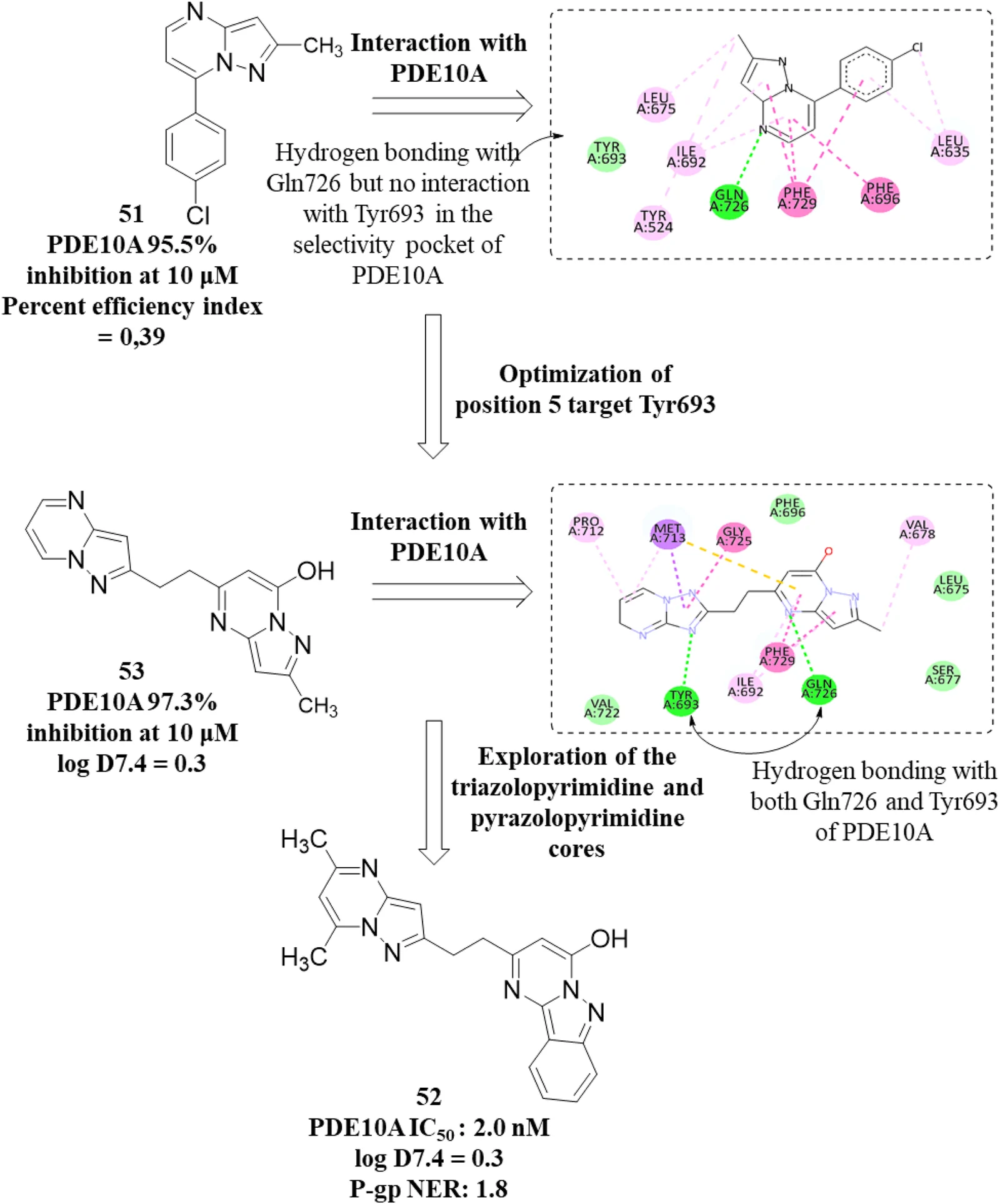
Structural modifications at the 7-position and triazolopyrimidine/pyrazolopyrimidine cores, resulting in Compound **52** and enhanced selectivity through hydrogen bonding with Y693.

**SCHEME 8:**
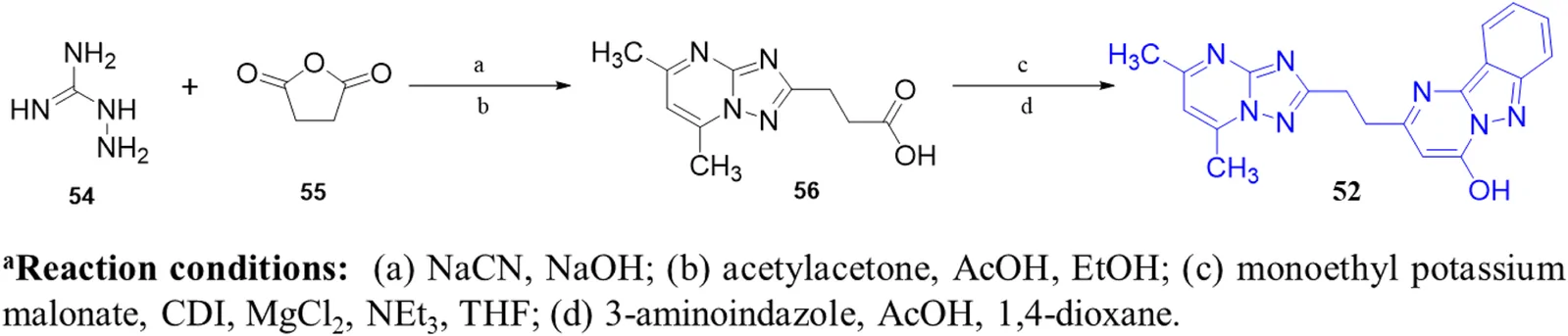
Synthesis of Compound **52**, as reported and executed by Chino et al. (2018) *.*

Chino et al. (2018) discovered a novel series of pyrimidoindazole derivatives as potent PDE10A inhibitors through fragment-based drug discovery (FBDD). The optimization began with Compound **51**, a fragment hit with promising ligand efficiency (PEI: 0.39) for PDE10A. Structural modifications led to the development of Compound **52**, a pyrimidoindazole derivative that exhibited potent PDE10A inhibition and favorable physicochemical properties. The X-ray co-crystallographic analysis revealed that the pyrazolopyrimidine ring of Compound **51** formed H-bonds with Q726 and hydrophobic interaction with the P-clamp in the PDE10A active site, although no interaction with Y693 in the selectivity pocket was observed (Figures 15A,B). To enhance binding to Y693 and improve inhibitory activity, the 4-chlorophenyl group at the 7-position was replaced with a hydroxyl group, aiming to reduce lipophilicity and promote stronger interactions. Subsequent modifications explored heteroaromatic substitutions with nitrogen atoms to target Y693. Among these, Compound **53**, a triazolopyrimidine derivative, demonstrated a balance of strong PDE10A inhibition (97.3% inhibition at 4 µM) and improved hydrophilicity (log D7.4: 0.3), making it a candidate for further optimization. Further refinement of the triazolopyrimidine and pyrazolopyrimidine cores yielded Compound **52**, which emerged as the most potent PDE10A inhibitor in the series, with an IC_50_ value of 2.0 nM (Figure 16). Crystallographic analysis confirmed that the 4-nitrogen atom of Compound **53** formed a key hydrogen bond with Y693, enhancing selectivity and potency (Figures 15A,B). Synthesis of Compound **52** began with the cyclocondensation of Compound **54** and succinic anhydride **55**, followed by condensation with acetylacetone to yield intermediate **56**. The final product was obtained by reacting **56** with monoethyl potassium malonate and 3-aminoindazole in 1,4-dioxane (Scheme 8) (Chino et al., 2018).

#### 4.1.4 Pyrazolo[1,5-a]pyrimidine derivative

**FIGURE 17:**
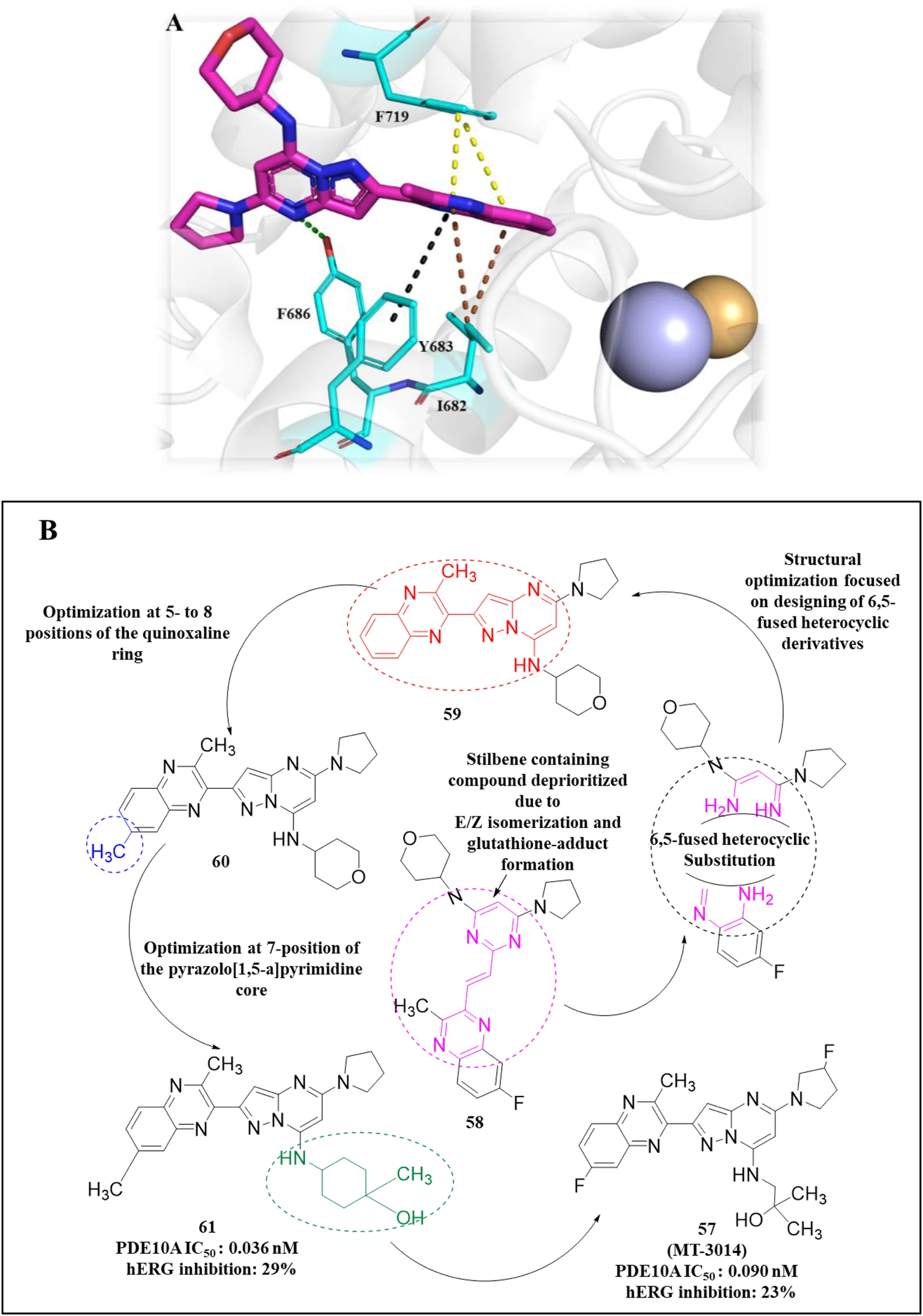
**(A)** Binding interaction of Compound **60** with PDE10A (PDB: 6ku9), illustrating the orientation of the pyrazolo[1,5-a]pyrimidine scaffold, π–π stacking of the quinoxaline moiety with F719 and F686, and a hydrogen bond between the pyrazolopyrimidine nitrogen and Y683. **(B)** Rational modification at the 7-position and quinoxaline ring yielding Compound **57**, with potent PDE10A inhibition, enhanced solubility, and decreased hERG liability.

**SCHEME 9:**
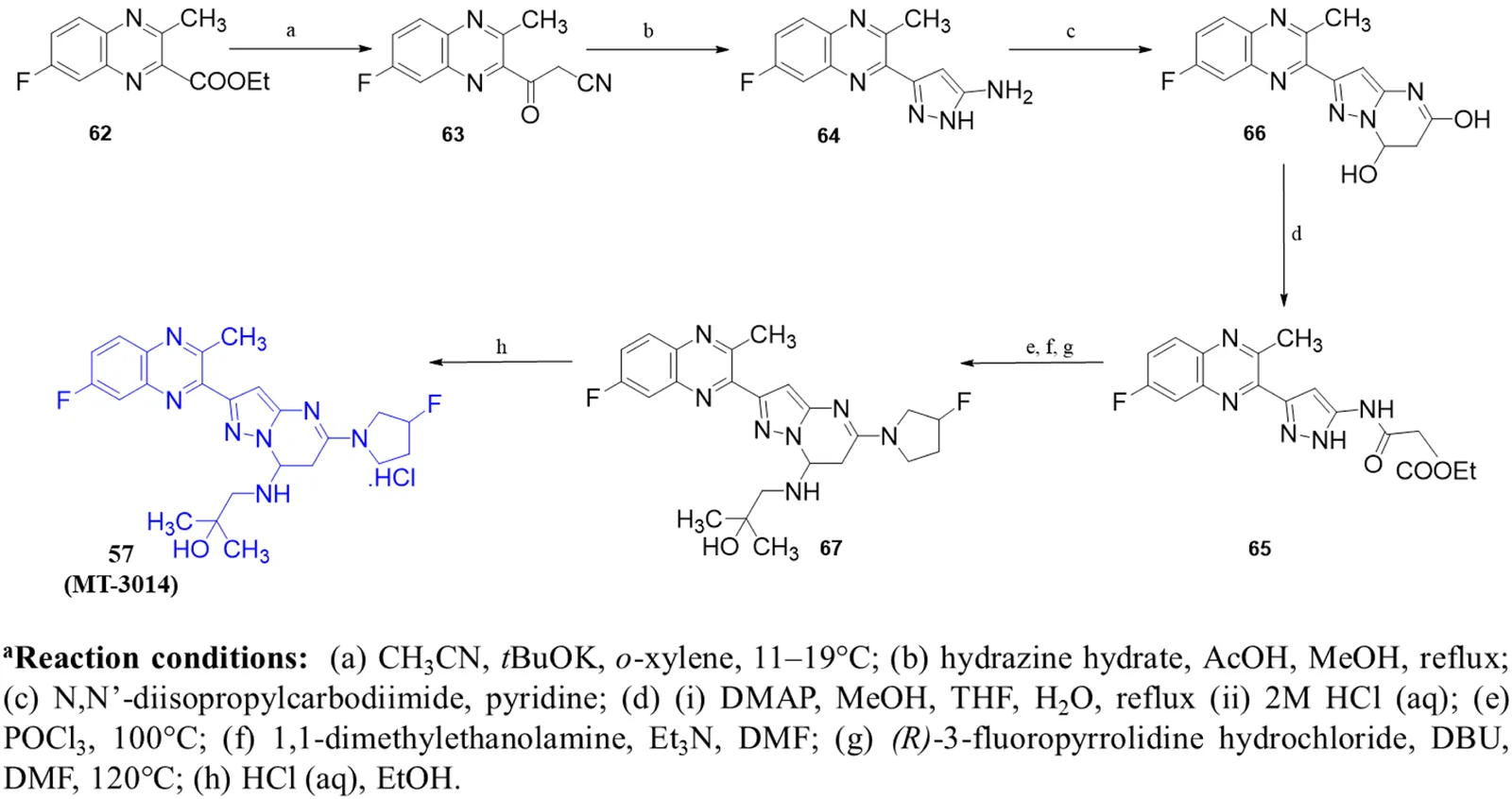
Synthesis of Compound **57**, as reported and employed by Koizumi et al. (2019) *.*

In another study, Koizumi et al. (2019) identified **57** (**MT-3014),** a potent pyrazolo[1,5-a]pyrimidine derivative, as a promising PDE10A inhibitor with an IC_50_ value of 0.062 nM. This compound emerged from a core structure transformation of a previously identified stilbene compound, **58**, which exhibited high selectivity for PDE10A but was deprioritized due to concerns over E/Z isomerization and glutathione-adduct formation. Compound **57** was selected for further profiling in clinical trials based on its favorable pharmacokinetic properties and efficacy in the rat-conditioned avoidance response test. Structural optimization focused on designing various 6,5-fused heterocyclic derivatives, which identified Compound **59** with the best PDE10A inhibitory activity among other derivatives. Further modifications targeted the 5- to 8-positions of the quinoxaline ring, yielding Compound **60**, which showed potent inhibition but exhibited strong hERG inhibition, necessitating additional optimization. Crystallographic analysis revealed that the quinoxaline core of Compound **60** formed key π–π interactions with F719 and F686 at the substrate-binding site of PDE10A. However, the steric clash between the 3-CH_3_ of quinoxaline and 3-H of the pyrazolopyrimidine ring controlled the torsion angle and binding properties. Furthermore, the nitrogen atom of the pyrazolopyrimidine ring makes a polar hydrogen bond with the OH of Y683 (Figure 17A). Subsequent modifications at position-7 of the pyrazolo[1,5-a]pyrimidine core led to the discovery of derivative **61**, which showed high PDE10A inhibition (IC_50_ of 0.036 nM) and improved solubilities, although Compound **61** also demonstrated reduced hERG inhibition (29%). Introducing a fluorine atom at position 3 of the quinoxaline ring further enhanced the solubility and reduced the lipophilicity, leading to the identification of Compound **57**, which balanced PDE10A inhibitory activity (IC_50_ of 0.090 nM), low hERG inhibition (23% at 1 µM), and favorable solubility in artificial intestinal fluid (118 μg/mL) (Figure 17B). **MT-3014 (57)** was synthesized *via* a linear route, starting with the reaction of quinoxaline **62** with an acetonitrile anion to produce Compound **63** with a 77% yield. Compound **63** was then refluxed with hydrazine hydrate to yield intermediate **64**, which reacted with N,N′-diisopropylcarbodiimide to afford Compound **65**. Further reaction of Compound **65** with DMAP resulted in the formation of Compound **66**. Chlorination of Compound **66** was performed using phosphorus(V) oxychloride, followed by successive treatment with methylated-ethanolamine and substituted pyrrolidine, which produced Compound **67**. Finally, Compound **67** was reacted with an HCl solution and recrystallized to obtain Compound **57** as an HCl salt (Scheme 9) (Koizumi et al., 2019).

#### 4.1.5 4H-chromone derivative

**FIGURE 18:**
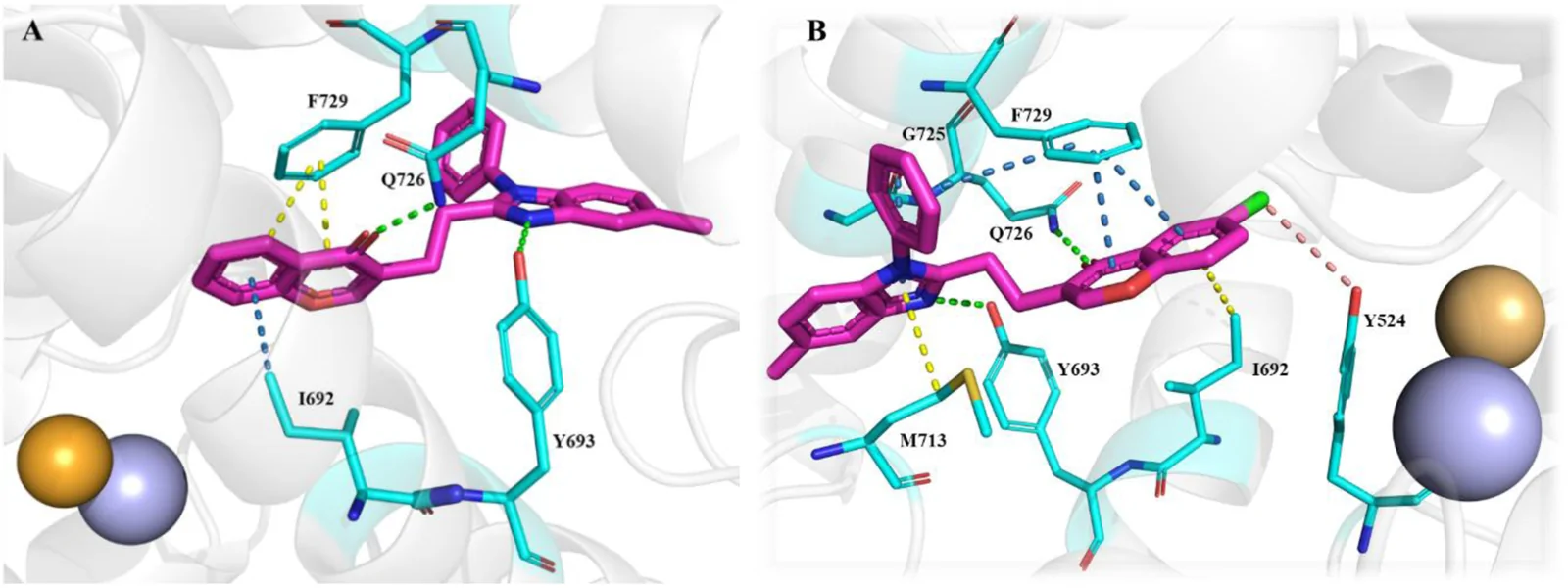
**(A)** Binding interaction of Compound **70** with PDE10A (PDB: 6ko0), depicting hydrogen bond interactions with F726/Y693 and proper positioning of the 5-methyl-1-phenyl benzimidazole moiety in the Q2 pocket. **(B)** Binding interaction of Compound **71** with PDE10A (PDB: 6ko1), showing an extra halogen bond formed between the chlorine atom of Compound **71** and Y524, which contributes to enhanced inhibitory activity.

**FIGURE 19:**
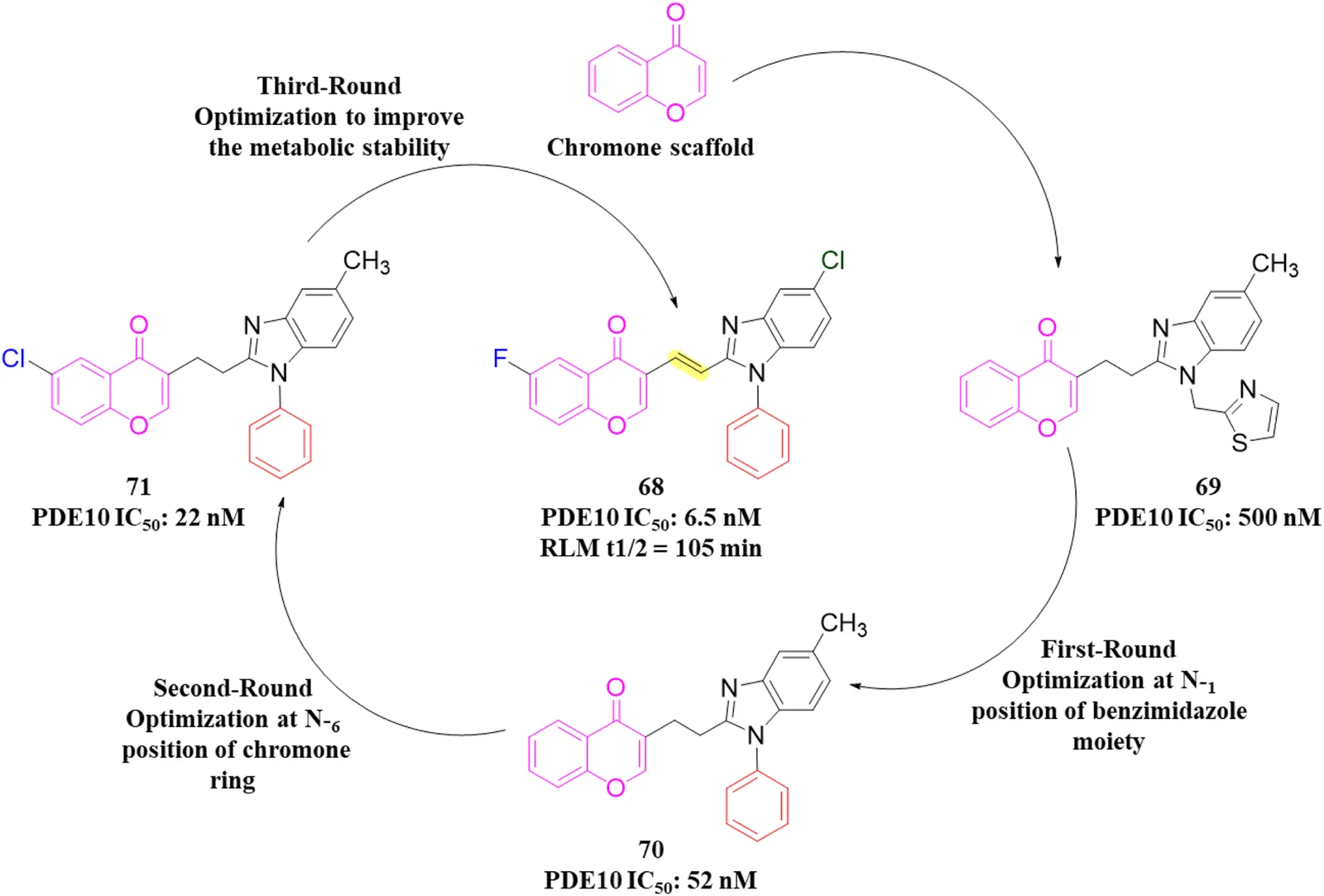
Structural refinement of Compound **68** through C-6 halogenation and ethyl-to-vinyl linker substitution, achieving enhanced PDE10A potency, selectivity, and metabolic stability.

**SCHEME 10:**
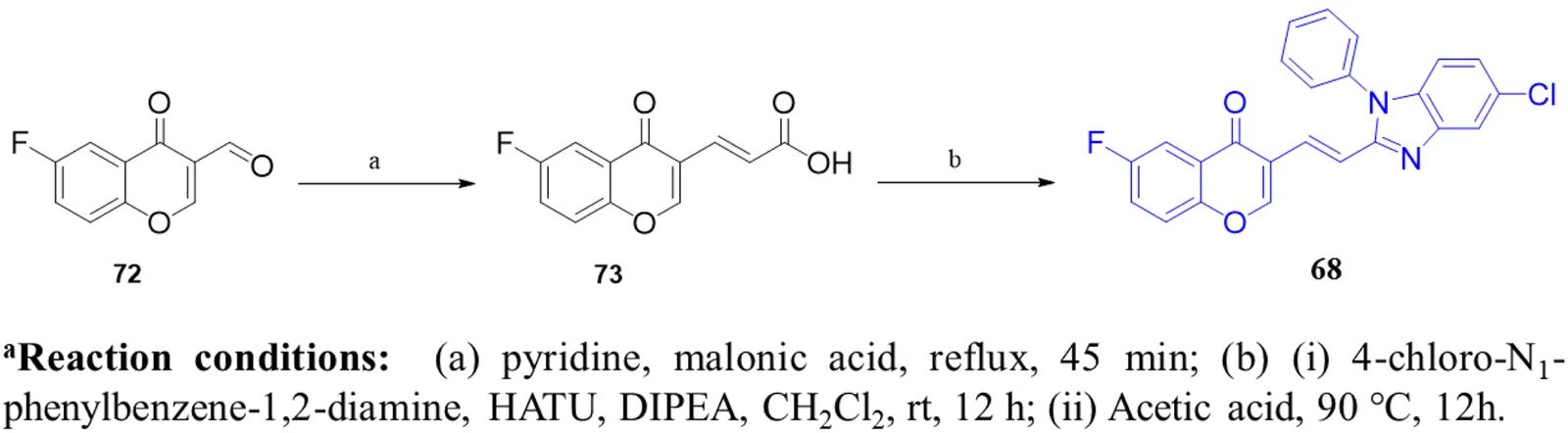
Synthesis of Compound **68**, as reported and employed by Yu et al. (2020) *.*

A novel chromone scaffold-containing molecule, Compound **68**, has been introduced as a PDE10 inhibitor, demonstrating significant PDE10 inhibition with an IC_50_ value of 6.5 nM, high selectivity (>95-fold over other PDEs), and excellent metabolic stability (RLM t_1/2_: 105 min). The development of Compound **68** resulted from three rounds of structure optimization and critical analysis of binding patterns with PDE10. The chromone scaffold of Compound **69** interacts with conserved Q726 *via* hydrogen bonding and with a hydrophobic clamp formed by F729 and I692/F696 through π–π stacking interactions. Additionally, the ethyl linker extends into the Q2 pocket, allowing the thiazol-5-yl methyl benzimidazole motif of 1 to form a hydrogen bond with Y693. As is known, the Q2 pocket provides unique selectivity to PDE10 inhibitors; therefore, structural optimization focused on the benzimidazole moiety was employed to enhance selectivity. The first-round optimization led to Compound **70**, which contains a 5-methyl-1-phenyl benzimidazole moiety and exhibited an excellent increase in potency (IC_50_: 52 nM). Compound **70** showed a 10-fold increase in potency compared to Compound **69**, indicating that the phenyl group at the N_5_-position of the benzimidazole moiety was preferred as it fit well into the Q2 pocket. The binding interaction analysis of Compound **70** with PDE10 showed that the 5-methyl-1-phenyl benzimidazole group of Compound **70** fit properly into the Q2 pocket. The oxygen atom of pyranone and the nitrogen atom of the benzimidazole group formed an H-bond with F726 and Y693 (Figure 18A). Further optimization at the C_6_-position of the chromone ring proposed that the halogenation at this position could enhance potency due to proximity (3.8 Å) to the phenolic hydroxyl group of Y524. The second-round optimization introduced chlorine at the C_6_-position, resulting in Compound **71**, which showed 2–3 times increased inhibitory activity compared to Compound **70**. The co-crystal structure of Compound **71** bound to PDE10A revealed that, in addition to existing interactions, an extra halogen bond formed between the chlorine atom of Compound **71** and Y524, contributing to enhanced inhibitory activity (Figure 18B). The third-round optimization aimed to improve the metabolic stability and pharmacokinetic properties of Compound **71**. Molecular docking suggested that a vinyl group, rather than an ethyl linker, would better extend into the Q2 pocket, resulting in molecules with improved metabolic stability and inhibitory potency. The extra halogen bond with Y524 indicated the C_6_-position of the chromone scaffold as a probable metabolic site. Introducing a fluorine atom at this position yielded Compound **68**, which demonstrated slightly improved inhibitory potency (IC_50_: 6.5 nM) and significantly enhanced metabolic stability (RLM t_1/2_: 105 min) (Figure 19). The binding mode of Compound **68** with PDE10A showed that the vinyl linker and benzimidazole group fit well into the narrow Q2 pocket, while the chromone scaffold engaged the hydrophobic clamp (F729 and F696/I692). Notably, H-bonds with Q726 and Y693, along with a halogen bond with Y524, contributed to the strong inhibitory potency of Compound **68**. Compound **68**, with a synthetic accessibility score of 3.27, indicates a moderately challenging synthesis. The synthesis of Compound **68** began with a Knoevenagel−Doebner condensation between the previously synthesized Compound **72** and malonic acid, yielding Compound **73**. Subsequent condensation of intermediate **73** with 4-chloro-N1-phenylbenzene-1,2-diamine, followed by cyclization, produced Compound **68** (Scheme 10) (Yu et al., 2020).

#### 4.1.6 Cyclopenta[4,5]thieno[2,3-d]pyrimidinone derivatives

**FIGURE 20:**
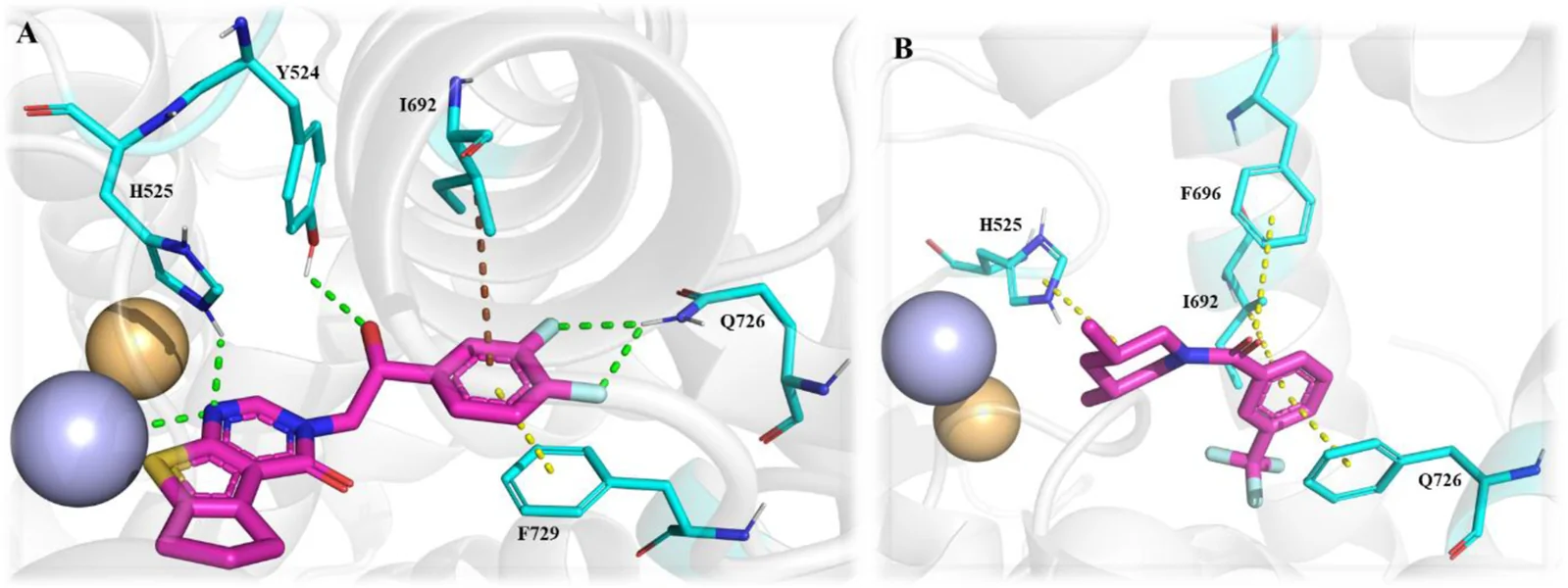
**(A)** Binding mode of Compound **74** with PDE10A (PDB: 6msa), highlighting scaffold orientation, P-clamp engagement, and key hydrogen bonds (Q726, Y524, H525, Mg) and stabilizing aromatic interactions. **(B)** Binding interaction of Compound **75** with PDE10A, showing P-clamp and aromatic contacts (F726, F696, and I692) and H525 hydrogen bonding, similar overall positioning but absent Q726/Y524 network.

In a recent study, Al-Nema et al. (2022b) identified **74 (Zinc42657360)**, containing a cyclopenta[4,5]thieno[2,3-d]pyrimidin-4-one scaffold, as a promising new PDE10A inhibitor. A systematic approach included a structure-based drug design program, combining pharmacophore modeling, molecular docking, and molecular dynamics simulations, followed by biological evaluation through the PDE-Glo phosphodiesterase assay, leading to the discovery of potent Compound **74**, with a PDE10A IC_50_ value of 1.60 µM. The development of Compound **74** involved a comprehensive virtual screening process. The screening identified six key pharmacophoric features in the co-crystallized structure of PDE10A (PDB ID: 5UWF) and the ligand 16d, which included one hydrogen bond acceptors (HBA) interacting with Q726, an aromatic ring (AR) represented by the thiophene moiety, and four hydrophobic areas (HA) represented by phenol, sulfide, and fluorine groups. A series of multi-step virtual screenings, such as pharmacophore-based screening, drug-likeness analysis, and pharmacophore fit score, resulted in 7541, 516, and 14 hits, respectively, which were subsequently subjected to the PAINS filter. These compounds passed the PAINS filter and were subjected to molecular docking studies. The docking studies revealed that compounds **74** and **75** (**Zinc47464611**) and the standard TAK063 exhibited the lowest binding energies, indicating high affinity for PDE10A. Structural analysis of Compound **74** with PDE10A showed that its cyclopenta-thiophene moiety occupies the P-clamp region, stabilized by aromatic interactions with the pyrimidine substituents. Notably, Compound **74** forms unique hydrogen bonds with Y524, Q726, and H525 and a coordination interaction with Mg, which collectively contribute to its high selectivity and affinity for PDE10A (Figure 20A). Compound **75** also engages the P-clamp region and forms aromatic interactions with F726, F696, and I692, along with interactions with H525 (Figure 20B). While both compounds **74** and **75** share P-clamp occupancy and aromatic contacts, Compound **74** distinguished itself by establishing unique hydrogen bonds.

**FIGURE 21:**
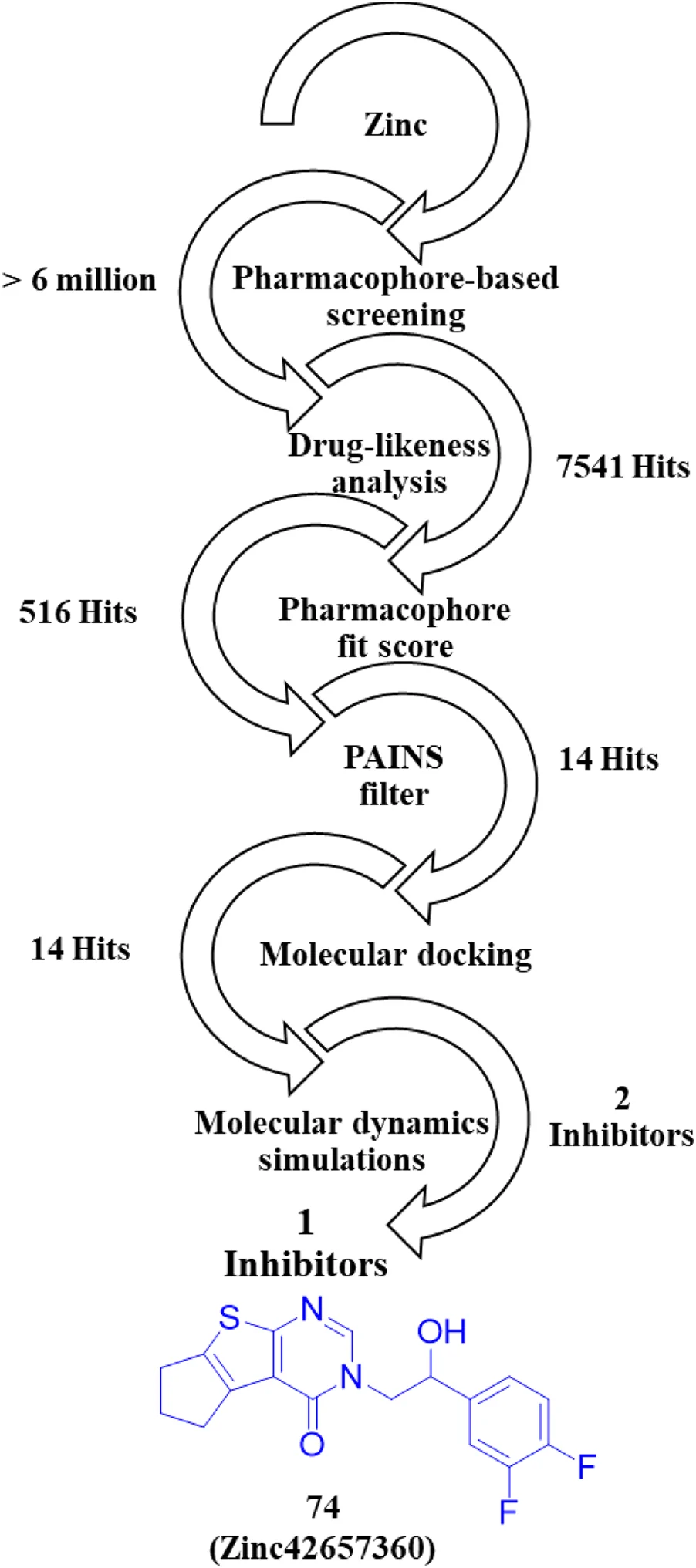
Workflow depicting virtual screening toward Compound **74**, emphasizing *in silico* methods, leading to a potent PDE10A inhibition (IC_50_: 1.6 µM).

Furthermore, the inhibitory potency of Compound **74** (IC_50_: 1.6 µM) was observed to be relatively weak compared with standard PDE10A inhibitors such as TAK-063 (IC_50_: 0.34 nM). Although the study provides a strong foundation for initial hit identification, it lacks critical follow-up characterization, including selectivity profiling against other PDE isoforms and essential ADME (Absorption, Distribution, Metabolism, and Excretion) parameters (e.g., aqueous solubility, microsomal stability, and plasma protein binding) required to assess the scaffold’s overall drug-likeness and development. Given the observed hydrogen-bonding interactions with Q726 and Y524 and considering the contribution of the P-clamp region to PDE10A selectivity, future optimization should prioritize (i) enhancing potency by incorporating structural constraints that properly orient key substituents to reinforce the Q726/Y524 hydrogen-bond network and (ii) modulating lipophilicity and polar surface area to achieve an optimal balance between CNS penetration and effective P-clamp engagement. The study demonstrated that a multistep *in silico* workflow (Figure 21), followed by *in vitro* analysis, enabled the identification of Compound **74**; however, the scaffold still requires comprehensive PK and preclinical assessment to establish its suitability as a suitable drug candidate (Al-Nema et al., 2022b).

#### 4.1.7 Methyl-amino-methoxypyrimidine derivatives

**FIGURE 22:**
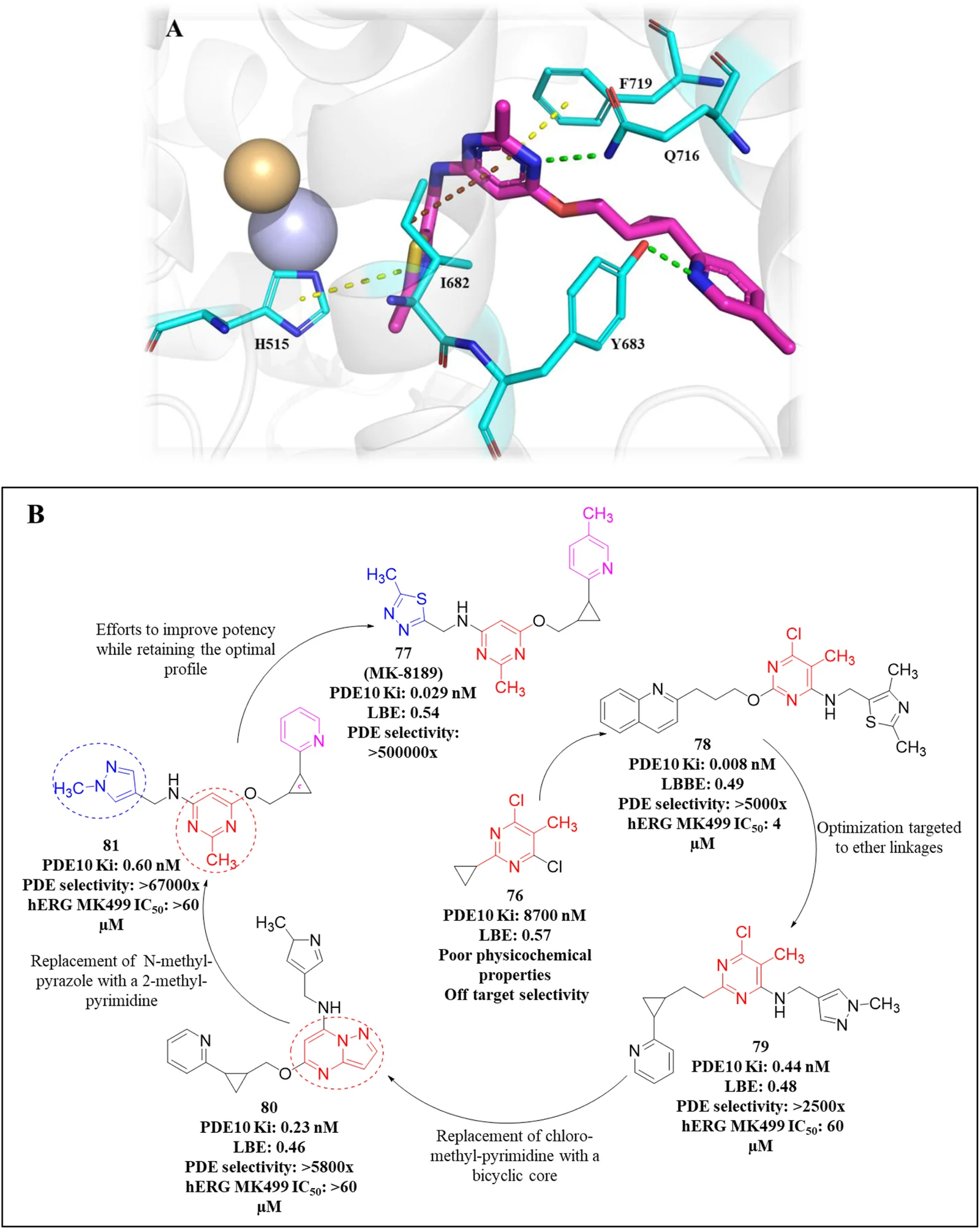
**(A)** Binding interaction of Compound **77** with PDE10A (PDB: 8DI4), highlighting the orientation of the bicyclic pyrazolopyrimidine core, π–π interactions with F719, hydrogen bond with Q716, and engagement of the 5-methylpyridine with Y683. **(B)** Rational structural optimization from pyrimidine **76** *via* intermediates **78–81**, incorporating ether and bicyclic motifs, culminating in Compound **77** with potent, selective, and PK-optimized PDE10A inhibition.

**SCHEME 11:**
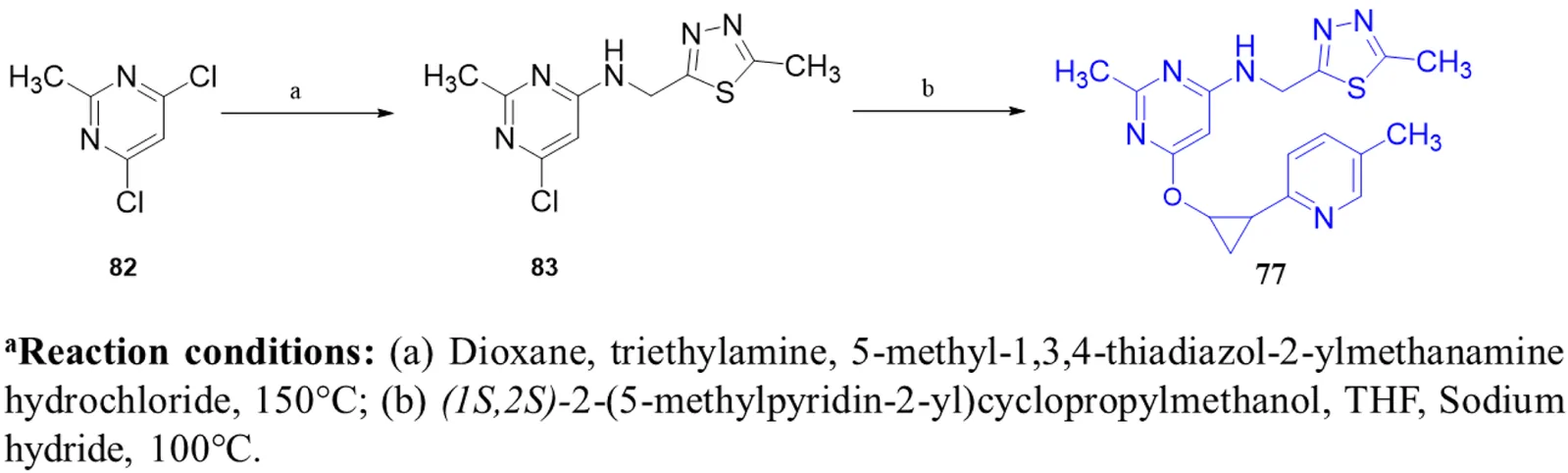
Synthesis of Compound **77**, as reported and employed by Layton et al. (2023) *.*

Layton et al. (2023) described a potent 2-methyl-4-amino-6-methoxypyrimidine inhibitor selective for PDE10A. Their earlier structural optimization efforts led to the identification of Compound **76**, a potent PDE10A inhibitor, with poor physicochemical properties and off-target activities. Furthermore, the rational design, combined with synthetic techniques and guided by inhibitor-bound X-ray crystal structures, led to the discovery of Compound **77 (MK-8189)** with a PDE10A Ki value of 1.6 nM and PDE selectivity >500,000. A novel scaffold, Compound **76**, was identified through a fragment screening as a PDE10A inhibitor with high ligand binding efficiency (LBE). It was optimized using structure-based design and parallel library synthesis, leading to the identification of Compound **78**. Compound **78** showed excellent potency, engaging key residues such as Y683 in the PDE10A selectivity pocket, but it still exhibited a poor pharmacokinetic profile, low aqueous solubility, and off-target ion channel activity against hERG. To address these challenges, further optimization targeted the ether linkages, resulting in Compound **79** with a methyl-pyrazole moiety. This step improved metabolic stability but still needed enhancement in CYP inhibition and clearance. Hypothesizing that the chloro-methyl-pyrimidine core was responsible for the remaining issues, they explored replacing it with a bicyclic core, leading to Compound **80**. This bicyclic pyrazolopyrimidine core improved permeability and minimized P-gp transporter liability. Subsequent optimization focused on improving potency by replacing the N-methyl-pyrazole with a 2-methylpyrimidine, yielding Compound **81**. This modification led to a notable improvement in selectivity, solubility, and pharmacokinetic properties, although with a slight compromise in potency. Efforts to improve potency while retaining the optimal profile led to the most balanced Compound **77**, which combines a methyl-pyridine and methyl-1,3,4-thiadiazole motif, achieving high potency, selectivity, and favorable PK, making it a lead candidate for further evaluation. Compound **77** demonstrated functional inhibition of PDE10A with a Ki value of 0.029 nM and over 500,000-fold selectivity over other PDE enzymes, making it a promising PDE10A inhibitor (Figures 22A,B). The co-crystal structure of Compound **77** with PDE10A reveals its binding interaction. The pendant 5-methyl-pyridine forms an H-bond with Y683 in the “selectivity pocket” of PDE10A, while the pyrimidine core of Compound **77** establishes π–π stacking with the side chain of F719. Additionally, a hydrogen bond is observed between N_1_ nitrogen and the side chain of Q716. The N_3_ nitrogen of the pyrimidine core is ideally positioned to form additional interactions with the binding site of PDE10A, enhancing the potency of Compound **77** (Figures 22A,B). The synthetic accessibility score and MW of Compound **77** were 4.13 and 382.48 g/mol, respectively, suggesting a more complicated synthetic strategy compared with other compounds (Daina et al., 2017). The synthesis started with pyrimidine **82**, which was exposed to a microwave-assisted reaction with (5-methyl-1,3,4-thiadiazol-2-yl)methanamine hydrochloride, yielding intermediate **83**. This intermediate was then treated with a mixture of ((1S,2S)-2-(5-methylpyridin-2-yl)cyclopropyl)methanol and tetrahydrofuran (THF), which yielded Compound 77 (Scheme 11). The SAR and mechanistic insights emerging from the MK-8189 highlight several key design principles. First, the pyrimidine-derived hinge-binding core provides a rigid π-stacking framework, most notably interacting with F719, while orienting heteroatoms to form essential H-bond interaction within the PDE10A catalytic pocket. Second, the pendant 5-methylpyridine, a motif preserved throughout late-stage optimization, projects deeply into the PDE10A selectivity pocket, forming a critical H-bond with Y683, which is a key contributor to the exceptional isoform selectivity of MK-8189. Third, systematic replacement of flexible ether linkages and metabolically vulnerable substituents with bicyclic and conformationally constrained motifs produced substantial improvements in physicochemical and ADME parameters, including increased solubility, enhanced microsomal stability, reduced CYP inhibition, and lower P-gp liability, while maintaining sub-nanomolar to picomolar potency (Layton et al., 2023).

## 5 Insight into dual PDE1B/PDE10A inhibitors

PDE inhibitors play a crucial role in modulating intracellular signaling pathways, particularly those involving cyclic nucleotides (Cardinale and Fusco, 2018). PDE1B and PDE10A are two enzymes co-localized with dopamine receptors and exhibit significant expression in the striatum, where they are believed to have overlapping functions (Russwurm et al., 2015). Their distribution and regulatory influence on dopaminergic signaling make them critical targets for addressing schizophrenia. PDE1B inhibition is hypothesized to enhance D_1_ receptor signaling, which can ameliorate the negative and cognitive deficits associated with schizophrenia (McQuown et al., 2019). Conversely, PDE10A inhibition reduces D_2_ receptor activity, which has the potential to alleviate positive symptoms (Al-Nema et al., 2022b). A dual PDE1B/PDE10A inhibitor offers a novel therapeutic approach for the comprehensive management of schizophrenia by simultaneously modulating D_1_ and D_2_ receptor signaling (Al-Nema et al., 2021). In recent studies, **84 (Zinc41306568)**, a dual PDE1B/PDE10A inhibitor, was evaluated for its pharmacological efficacy and safety in a rat model of schizophrenia. The compound significantly suppressed ketamine-induced hyperlocomotion, a model for the positive symptoms of schizophrenia. Furthermore, it effectively reduced social isolation caused by chronic ketamine administration and improved cognitive function, as demonstrated by enhanced recognition memory in the NOR test. These findings highlight the potential of **84 (Zinc41306568)** to reverse and prevent schizophrenia-like behavioral alterations, presenting a comprehensive approach to schizophrenia treatment (Al-Nema et al., 2021). Further investigations in this area are outlined below.

### 5.1 Benzoxazine derivative

**FIGURE 23:**
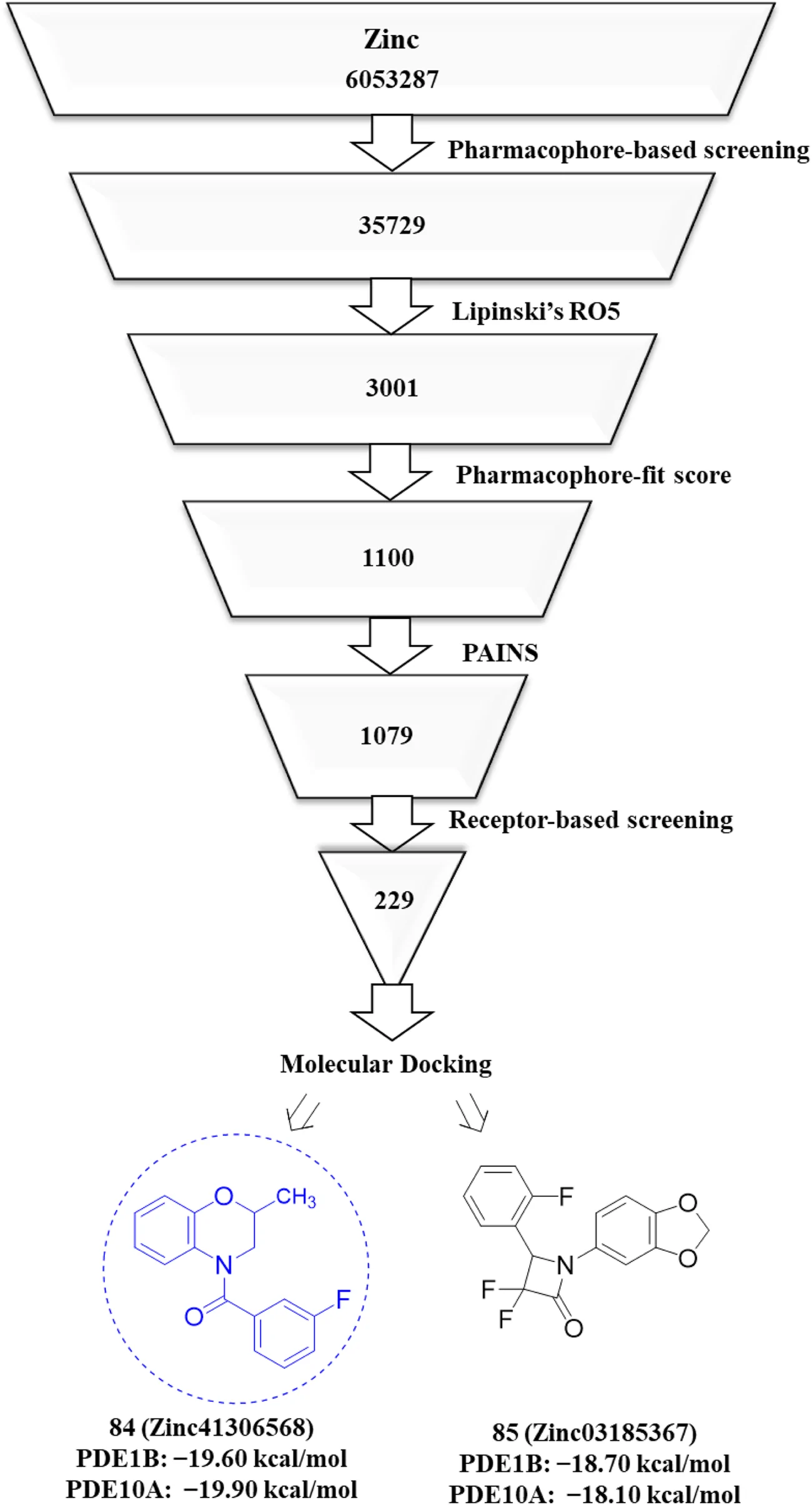
Optimization strategy leading to Compound **84**, from virtual screening to *in vitro* evaluation, resulting in dual-target inhibition.

**FIGURE 24:**
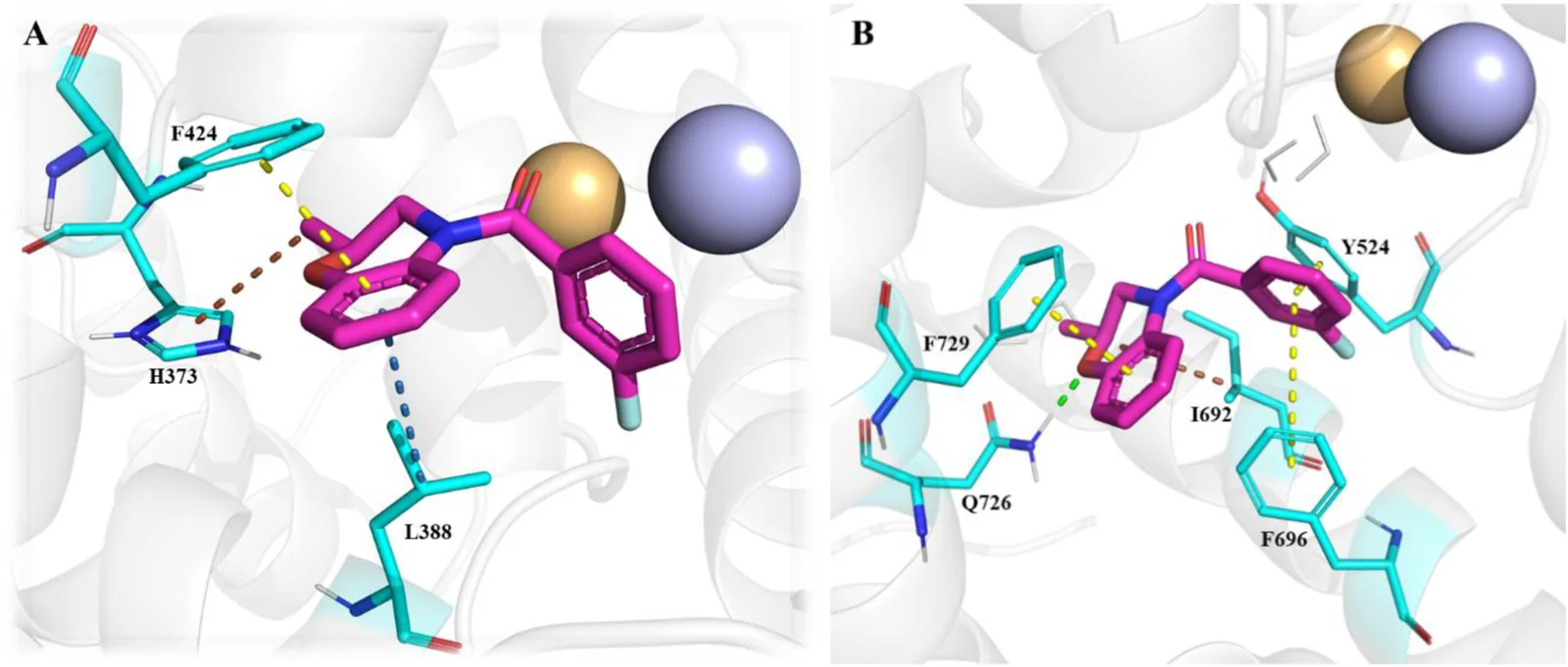
**(A)** Binding interaction between Compound **84** and PDE1B (PDB: 5b25), showing the cyclopenta[4,5]thieno[2,3-d]pyrimidin-4-one scaffold orientation within the active site, hydrophobic interactions with F424, H373, and L388, and key functional groups (phenyl, amine, and fluorine) contributing to ligand binding. **(B)** Binding interaction between Compound **84** and PDE10A (PDB: 6msa), illustrating occupancy of the P-clamp region, hydrophobic interactions with F692, I692, and F729 and hydrogen bonding with Q726 and Y524.

Al-Nema et al. (2021) reported the discovery of a dual PDE1B/10A inhibitor, Compound **84 (Zinc41306568)**, with IC_50_ values of 0.85 µM for PDE1B and 1.34 µM for PDE10A. The design strategy for Compound **84** employed a computer-aided drug design approach that integrated pharmacophore-based screening, Lipinski’s rule of five, pharmacophore fit scoring (PFS), PAINS filtering, and receptor-based screening. This systematic process initially yielded 35,729 hits, which were sequentially narrowed down to 3001, 1100, 1079, and finally 229 hits after successive filtering steps. Molecular docking studies of these 229 compounds identified two lead candidates, compounds **84 (Zinc41306568)** and **85 (Zinc03185367)**, with the lowest binding energies of −18.70 kcal/mol and −19.60 kcal/mol, respectively, for both PDE1B and PDE10A (Figure 23). Furthermore, the molecular docking studies of TAK-063, a reference for PDE10A, and 6-(2-chlorobenzyl)-8,9,10,11-tetrahydrobenzo[4,5]thieno[3,2-e][1,2,4]triazolo[1,5-c]pyrimidin-5(6H)-one, a reference for PDE1B, were performed. TAK-063 showed a docking score of −17.60 kcal/mol, whereas the PDE1B standard exhibited a binding energy of −17.20 kcal/mol. Notably, both compounds **84** and **85** demonstrated better binding energies than references, thereby supporting their high-affinity interactions within the catalytic domains of both PDE1B and PDE10A. The binding interaction analysis revealed that within the PDE1B active site, compounds **84** and **3** (standard) formed hydrophobic interactions with F424, H373, and L388 (Figure 24A). For PDE10A, docking analysis exhibited that compounds **84** and **85** and TAK-063 (standard) all occupied the P-clamp and interacted with key residues, including F692, I692, and F729. Notably, all three ligands exhibited binding with Q726, although Compound **84** displayed additional interactions with Y524, highlighting its distinct binding profile (Figure 24B). These findings highlight the critical role of specific functional groups, such as phenyl, amine, and fluorine, in mediating effective binding interactions with PDE1B and PDE10A. The docking results provided valuable insights into the preferred orientations and binding modes of these ligands, establishing Compound **84** as a promising dual PDE1B/10A inhibitor for further development. Furthermore, Compound **84** exhibited dual PDE1B/PDE10A inhibition within the sub-micromolar range, although its potency remains lower than that of the respective standard inhibitors for each isoform. While the dual-target strategy is therapeutically attractive, the advancement of such a molecule necessitates rigorous ADME, pharmacokinetic, and safety evaluations. In particular, ensuring adequate CNS exposure, maintaining appropriate isoform selectivity margins, and establishing a tolerable safety profile are essential requisites for its development as a drug (Al-Nema et al., 2021).

### 5.2 Indolizino[1,2-b]quinolinone derivative

**FIGURE 25:**
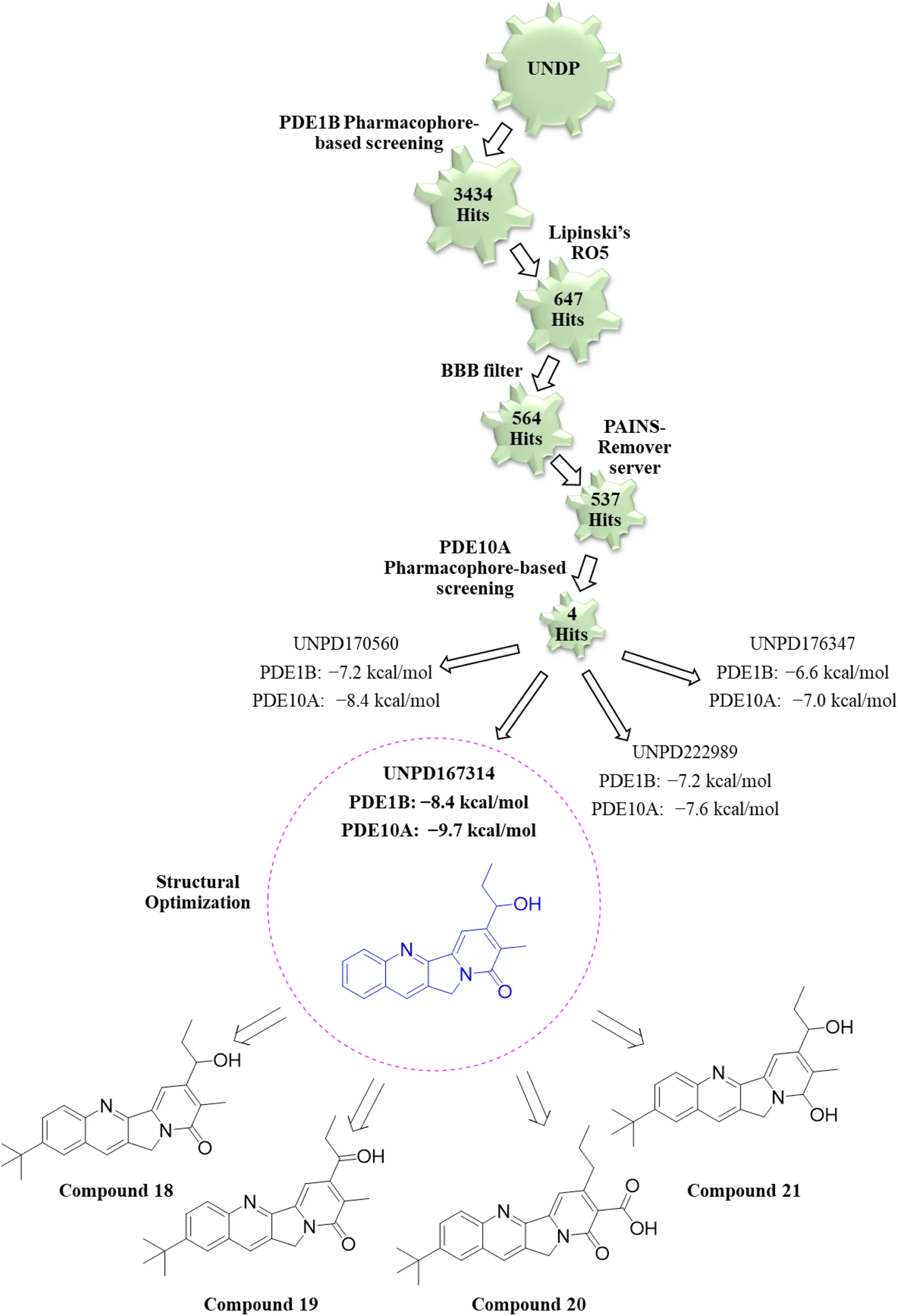
Discovery workflow for Compound **86**, including virtual screening of UNPD, pharmacophore modeling, Lipinski’s rule, BBB filtering, PAINS removal, and PDE10A pharmacophore fitting, resulting in the identification of dual PDE1B/PDE10A inhibitors **87–90**.

**FIGURE 26:**
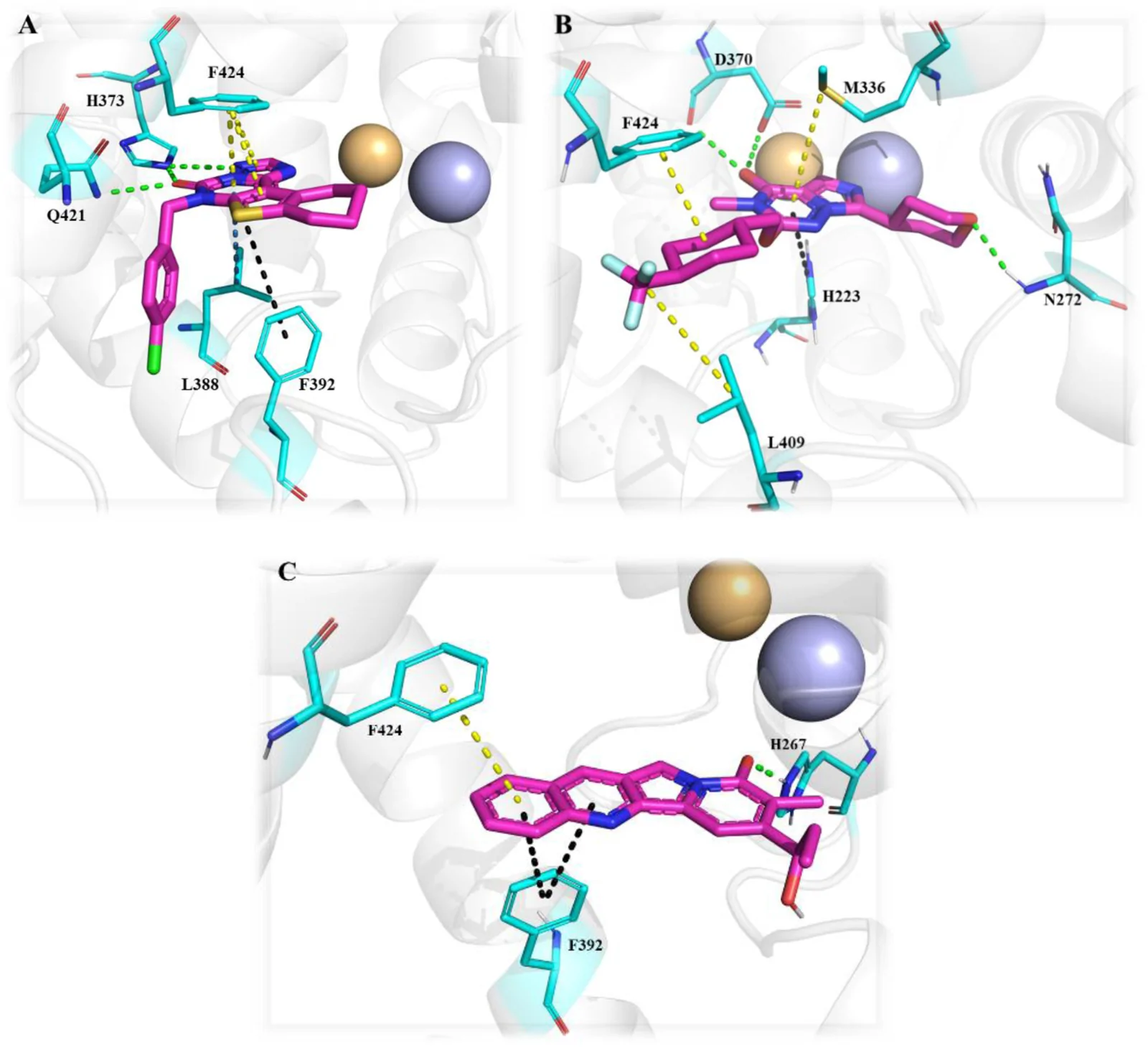
Binding interaction of inhibitors with PDE1B (PDB: 5up0). **(A)** Binding interactions of 8HP with PDE1B (PDB: 5up0), showing key interaction such as hydrogen bonding with H373 and Q421 and hydrophobic interactions with L388, F392, and F424. **(B)** Binding interactions of DSR-141562 with PDE1B, illustrating the hydrophobic contacts with L388, F392, F424, and H223 and hydrogen bonding with D370 and Y222. **(C)** Binding interactions of Compound **86** with PDE1B, showing the indolizino[1,2-b]quinolinone scaffold orientation within the active site, hydrophobic interactions with F392 and F424, and hydrogen bond with H267, highlighting its PDE1B binding potential.

**FIGURE 27:**
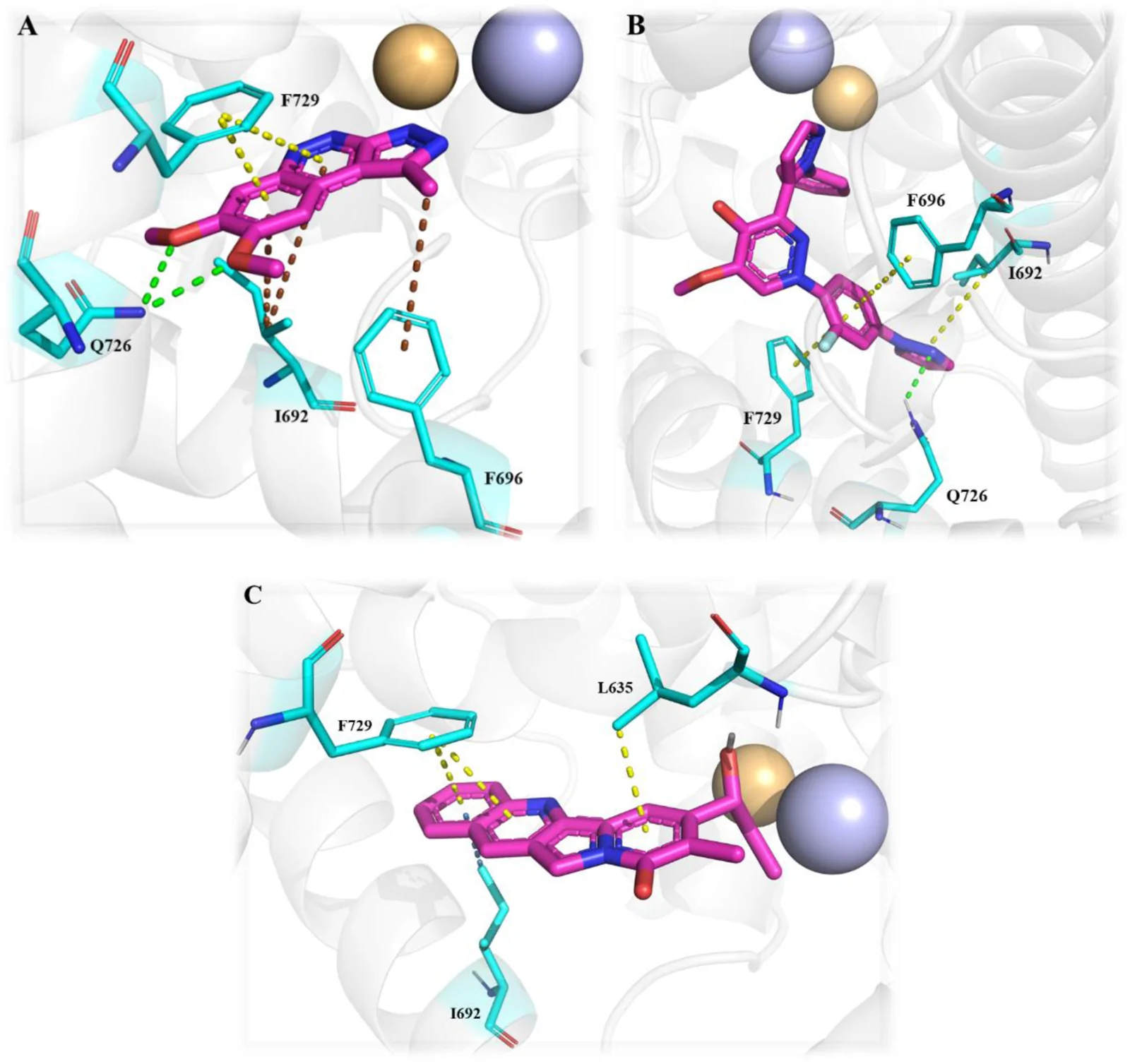
Binding interaction of inhibitors with PDE10A (PDB: 6msa). **(A)** Binding interactions of JY4 with PDE10A, showing occupancy of the P-clamp, hydrophobic interactions with I692, F696, and F729, and hydrogen bonds with D674 and Y524. **(B)** Binding interactions of TAK-063 with PDE10A, illustrating P-clamp occupancy, hydrophobic interactions with I692, F696, and F729, and hydrogen bonding with Q726. **(C)** Binding interactions of Compound **86** with PDE10A, illustrating occupancy of the P-clamp region and hydrophobic contacts with I692, F696, and F729.

In another study, Soon et al. (2024) described novel derivatives as dual PDE1B/PDE10A inhibitors. Their systematic approach integrated ligand-based pharmacophore modeling and multi-step virtual screening with molecular docking studies to identify promising compounds. The initial screening of the Universal Natural Product Database (UNPD) using the PDE1B pharmacophoric features resulted in 3434 hits. These hits were further refined through Lipinski’s RO5, the BBB filter, the PAINS-Remover server, and a PDE10A pharmacophore model, yielding 647, 564, 537, and 4 hits, respectively. Subsequent molecular docking studies of 4 hits revealed that Compound **86 (UNPD167314)** exhibited the lowest binding energies of −8.4 kcal/mol and −9.7 kcal/mol for PDE1B and PDE10A, respectively (Figure 25)**.** The binding interaction analysis compounds **86**, 8HP (co-crystallized ligand), and DSR-141562 (standard) showed that all ligands occupied the P-clamp and interacted with key hydrophobic residues L388, F392, and F424. Notably, Compound **86** engaged only F392 and F424 and formed a single hydrogen bond with H267, whereas 8HP and DSR-141562 displayed additional interactions with H373, Q421, D370, and Y222, with 8HP exhibiting the highest affinity due to extra aromatic contacts (Figures 26A–C). For PDE10A, Compound **86**, JY4 (co-crystallized ligand), and TAK-063 (standard), all occupied the P-clamp, interacting with I692, F696, and F729. Only JY4 and TAK-063 formed hydrogen bonds within the active site, whereas Compound **86** lacked hydrogen bonding, potentially explaining its lower affinity (Figures 27A–C).

SAR studies contributed to the design of 35 novel dual inhibitors, among which four compounds, compounds **87 (compound 18)**, **88 (compound 19)**, **89 (compound 20)**, and **90 (compound 21)** exhibited comparable binding affinities to PDE1/10A. The study identifies the indolizino[1,2-b]quinolinone scaffold as a promising framework for dual PDE1B/PDE10A inhibition; however, the docking-based affinity estimates and virtual SAR trends remain computational predictions and therefore require experimental validation. Advancement of these hits into biochemical PDE inhibition assays and early ADME characterization (including aqueous solubility, microsomal stability, and P-gp interaction assessment) is essential to determine whether the proposed binding modes translate into quantifiable enzymatic potency and brain-penetrant pharmacokinetic profiles (Soon et al., 2024).

## 6 Scaffold-specific ADME/PK trends in PDE1B, PDE10A, and dual PDE1B/PDE10A inhibitors

A comparative evaluation across scaffolds demonstrates characteristic ADME/PK patterns that are pivotal for translational optimization. Pyrazolo[4,3-e]pyrimidinone derivatives such as **ITI-214** deliver exceptional potency (Ki: 58 pM) but are hindered by high MW (507.6 g/mol) and synthetic complexity, limiting oral permeability (Li et al., 2016). In contrast, the thienotriazolopyrimidinone scaffold (**DNS-0056**) shows superior oral bioavailability and CNS exposure, although substitutions at C-6/C-8/C-9 markedly affect MDR1 efflux (Dyck et al., 2017). Pyrazolo[3,4-d]pyrimidinone hybrids (compound **18**) exhibits a more favorable balance between potency, metabolic stability, and MW (497.54 g/mol). Small hydrophobic N-7 substituents enhance potency but may reduce microsomal stability, requiring stabilizing modifications at N-2 (e.g., oxetane and sulfonylbenzyl groups). Lead compound **18** demonstrated good intrinsic clearance (rat microsomes, t½: 28.5 min) and chemical stability, indicating that this scaffold tolerates fine-tuning without compromising PDE1 selectivity (Zhang et al., 2021). Dihydrobenzofuran-based PDE1 inhibitors (compounds **27** and **28**), although lacking *in vivo* PK evidence, exhibit promising *in silico* properties (moderate MW: 450–470 g/mol and balanced lipophilicity) (Al-Nema et al., 2023). Quinazoline-based PDE1 inhibitors (compounds **31** and **32**) were specifically designed for CNS penetration; their moderate lipophilicity and low efflux liability support good brain exposure and moderate clearance, making them among the most CNS-compatible PDE1 scaffolds (Humphrey, 2014). Furthermore, PDE10A chemotypes exhibit distinct ADME/PK profiles that critically influence their translational development. Imidazo[1,2-a]pyrazine derivatives (Compound **40**) display moderate lipophilicity, high microsomal stability, favorable brain penetration, and strong *in vivo* exposure, although bulky R-group substitutions can impair permeability (Bartolomé-Nebreda et al., 2014). Imidazo-benzimidazoles (Compound **47**) exhibit high potency but poor CNS penetration due to strong P-gp efflux. Transition to the triazolopyrazine core (Compound **48**) reduces efflux and improves permeability, metabolic stability, and CYP cleanliness, highlighting the importance of nitrogen positioning in fused heterocycles (Chino et al., 2019). Fragment-derived pyrimido[1,2-b]indazoles (Compound **52**) maintain high ligand efficiency with low MW, very low lipophilicity (logD_7_._4_: 0.3), excellent microsomal stability, negligible CYP inhibition, and minimal P-gp efflux, yielding robust CNS penetration (Chino et al., 2018). Pyrazolo[1,5-a]pyrimidines (**MT-3014**) combine sub-nanomolar potency with balanced lipophilicity, high solubility, favorable clearance, and reduced hERG risk, facilitating transition to clinical studies (Koizumi et al., 2019). Chromone-derived PDE10 inhibitors (Compound **68**) show high metabolic stability (rat microsomal, t½: 105 min) and strong selectivity, with C-6 substitution improving potency and stability but requiring careful control to avoid solubility loss (Yu et al., 2020). Furthermore, beyond individual scaffold properties, general ADME/PK parameters such as pKa, pH-dependent ionization, and lipophilicity can influence binding orientation and promote ligand inversion, thereby favoring off-target activity. For instance, thienotriazolopyrimidinone derivatives such as Compound **10**, initially optimized as PDE1B inhibitors, adopt an inverted binding pose in PDE10A, resulting in unexpectedly high PDE10A potency. In this off-target interaction, the triazole nitrogen engages the conserved glutamine residue, and the carboxamide side chain projects into a narrow lipophilic channel. This channel is unfavorable to ionized groups, explaining why neutral or amide-substituted inhibitors can bind PDE10A more favorably than protonated amines (Dyck et al., 2017).

Emerging dual PDE1B/PDE10A scaffolds also reveal common ADME/PK requirements. The benzoxazine derivative Compound **84** demonstrates sub-micromolar dual potency and a CNS-oriented physicochemical profile, although *in vivo* PK evidence is still needed to validate brain exposure and metabolic stability (Al-Nema et al., 2021). Conversely, indolizino[1,2-b]quinolinones (compounds **86–90**), despite being CNS-oriented, exhibit limited hydrogen-bonding within catalytic pockets, a feature often associated with reduced microsomal stability and increased clearance (Soon et al., 2024). Collectively, these analyses indicate that effective dual PDE1B/PDE10A inhibitors will require finely tuned physicochemical properties, strong metabolic stability, and minimal efflux liability to achieve sustained brain exposure with adequate selectivity.

## 7 Clinical failures and translational limitations of PDE1B and PDE10A inhibitors in schizophrenia

Given compelling preclinical data supporting PDE1B and PDE10A inhibition as therapeutic strategies for schizophrenia, the translation of these findings into clinical success has been significantly limited (Table 2). Several mechanistic, pharmacokinetic, and methodological factors collectively contribute to this limited progression. A primary barrier is achieving adequate and sustained CNS target engagement at tolerable doses. In a randomized, parallel-group, 6-week study in adults with acutely exacerbated schizophrenia, TAK-063, a selective PDE10A inhibitor, administered at 20 mg daily, was compared with placebo. Although the drug was safe and generally well tolerated, with most adverse events being mild to moderate, the primary endpoint change in total Positive and Negative Syndrome Scale (PANSS) score was not met (least-squares mean difference vs. placebo: −5.46; p: 0.115; effect size: 0.308). Secondary endpoints, including PANSS subscales, BNSS, and CGI scores, showed nominal improvements but did not reach statistical significance. Several factors may contribute to these negative outcomes. First, the high placebo response, observed early and persisting throughout the trial, reduced the detectable treatment effect. Second, the study employed a single fixed dose without dose-ranging or active reference, limiting interpretation of efficacy across the dose-response relationship. Third, species-specific differences in PDE10A expression and dopaminergic circuits may have restricted the translation of observed preclinical efficacy to clinical (Macek et al., 2019).

Furthermore, the mechanistic specificity of selective PDE1B or PDE10A inhibition also exhibits notable translational limitations. Selective inhibition of PDE10A or PDE1B targets only specific symptom domains of schizophrenia. Consequently, administering a single selective inhibitor is insufficient to address the full spectrum of symptoms, necessitating the concurrent use of antipsychotics to manage the residual symptoms. This approach may, however, influence the overall therapeutic effectiveness of the selective inhibitor. For example, the selective PDE10A inhibitor RG7203 demonstrated minor improvement of reward-related brain activity at low doses; however, the drug ultimately worsened motivational and cognitive performance in patients. This unexpected outcome likely resulted from indirect attenuation of dopaminergic signaling due to combined PDE10A inhibition and D_2_ receptor blockade from antipsychotics, producing excessive suppression of dopaminergic signals. These outcomes highlight the intrinsic limitations of extrapolating from animal models, where dopamine circuitry and PDE10A distribution differ substantially from the chronically medicated, pathophysiologically altered human brain (Umbricht et al., 2021).

Similar translational discrepancies were observed with the selective PDE10A inhibitor MP-10. Although MP-10 significantly induced early gene expression (c-fos, egr-1, and arc) and activated both D1-and D2-modulated pathways in rodents, confirming target engagement, the behavioral outcomes did not align with therapeutic expectations. Co-administration of MP-10 and haloperidol studies revealed that even modest D_2_ antagonism profoundly altered the functional impact of PDE10A inhibition, exaggerating striatal activation at intermediate doses while abolishing these effects at higher exposures. These nonlinear, dose-dependent, and circuit-dependent interactions underscore the vulnerability of PDE10A modulation to background dopaminergic signal and highlight a key translational barrier: balanced activation of striatal medium spiny neuron (MSN) pathways observed in rodents does not reliably predict therapeutic outcomes in patients with chronic D_2_ blockade and altered corticostriatal network dynamics (Gentzel et al., 2015).

Another clinical candidate, the selective PDE10A inhibitor, MK-8189, further illustrates these challenges. Despite achieving sustained enzyme occupancy (∼65%) and showing small-to-moderate improvements in PANSS scores, MK-8189 may produce antipsychotic effects and associated weight loss. Species-related differences in PDE10A distribution suggested by differential responses among subgroups may have contributed to these inconclusive outcomes (Mukai et al., 2024).

Several other studies, including evaluations of the selective PDE10A inhibitor Lu AF11167 and the PDE1B inhibitor ITI-214, also exhibited suboptimal efficacy in clinical studies (Li et al., 2016; Meyer-Lindenberg et al., 2022). Additionally, the limited capacity of current animal models to fully recapitulate the multidimensional spectrum of schizophrenia, encompassing positive, negative, and cognitive symptoms, likely contributes to the poor translational success from preclinical to clinical. Collectively, these translational challenges provide a rationale for investigating dual PDE1B/PDE10A inhibition, which has the potential to achieve broader modulation of dopaminergic circuits and to improve symptomatic outcomes at lower, potentially safer doses compared with single-target approaches.

## 8 PDE1B/10A inhibition in schizophrenia: unresolved challenges and strategic future directions

Despite the promising link between PDE1B/PDE10A and schizophrenia, the translational success of PDE1B and PDE10A inhibitors has remained limited. A fundamental hurdle stems from the significant structural homology shared between the catalytic domains of PDE isoforms, particularly within the binding pocket regions. This homology complicates the rational design of isoform-selective inhibitors and frequently results in off-target selectivity, contributing to the failure of several candidates in clinical trials (Goff et al., 2009; Shirayama et al., 2011). Recent studies have identified key residues that can be exploited to achieve isoform selectivity for PDE1B and PDE10A. For PDE1B, selectivity can be enhanced by targeting unique active-site residues, including His373 and Gln421. Incorporation of small, sterically optimized substituents that interact with these residues may reduce off-target activity. In the case of PDE10A, selectivity is governed not only by the conserved glutamine and metal-binding residues but also by the Q2 sub-pocket, which comprises residues such as Y693. Ligand engagement with these sub-pocket residues enables discrimination from other PDE isoforms while preserving potent catalytic inhibition (Li et al., 2025). Collectively, a rational design strategy that integrates structure-guided optimization and targeted exploitation of isoform-specific residues within both the active site and selectivity pockets can minimize off-target interactions and enhance both safety and translational potential. Further complicating this landscape is the “glutamine switch” mechanism, a conformational determinant involving conserved glutamine residues that influence cyclic nucleotide specificity. While crystallographic studies have delineated this mechanism with structural clarity, recent studies challenge its universality under physiological conditions (Wang et al., 2007; Ke et al., 2011). This discrepancy highlights a critical limitation in current structure-based drug discovery paradigms, which often rely on static crystallographic snapshots that may inadequately capture the dynamic conformational plasticity of PDE enzymes in their native biological milieu.

Moreover, although selective PDE1B and PDE10A inhibitors have demonstrated preclinical efficacy in ameliorating both positive and negative symptom-like behaviors, clinical translation has encountered challenges, including inadequate CNS penetration, dose-limiting toxicities, and variable target engagement. For example, clinical trials evaluating PDE1 and PDE10A inhibitors have yielded inconsistent outcomes, with some compounds showing improvements in cognitive or negative symptoms while others failed to achieve primary outcomes (Table 2).

Another key obstacle lies in the dose balancing of dual inhibition. Achieving therapeutic efficacy against both PDE1B and PDE10A is confounded by their distinct tissue distribution, kinetic behavior, and differential roles in intracellular signaling. A dose optimized for one isoform may elicit sub-therapeutic effects or dose-limiting toxicity for the other. Furthermore, dual inhibitors frequently encounter challenges associated with off-target selectivity, poor brain penetration, and metabolic instability, which may contribute to the lack of clinical advancement of any single PDE1B/10A inhibitor to date.

To overcome these multifaceted challenges, novel therapeutic strategies such as proteolysis-targeting chimeras (PROTACs) have garnered attention. Unlike conventional occupancy-driven inhibitors, PROTACs facilitate the selective degradation of target proteins, thereby offering an opportunity to achieve sustained and isoform-specific knockdown. This strategy can potentially overcome challenges related to isoform compensation, poor target residence time, and adverse PK. Additionally, integrating imaging-based approaches such as positron emission tomography (PET) to quantify *in vivo* target occupancy would strengthen the clinical translatability of PDE1B/PDE10A-directed PROTACs. Recent advances in small-molecule radiotracer development demonstrate that PET ligands, originally optimized for mapping protein expression in psychiatric disorders, possess considerable utility for translational degrader studies. For example, emerging PROTAC studies have shown that PET tracers can be repurposed as tool degraders to validate target accessibility and intracellular protein removal, as illustrated by tau-directed and cereblon (CRBN)-recruiting degraders (Sun et al., 2021; Xiao et al., 2022). Further evidence indicates that structurally refined imaging ligands designed for robust brain penetration and high target selectivity can be adapted as radiochemical platforms for molecular neuroimaging (Zhang et al., 2017). Moreover, advances in radioligand chemistry and cyclic-nucleotide signaling PET methodologies (Schröder et al., 2021) support the feasibility of developing PDE-selective PET tracers capable of mapping regional enzyme distribution *in vivo*. Incorporating such PET-enabled methodologies into PDE1B/PDE10A PROTAC discovery programs would provide rigorous confirmation of CNS exposure, dose-dependent target degradation, and pharmacodynamic persistence, which are key determinants for advancing schizophrenia therapeutics. Encouragingly, PROTACs have shown therapeutic potential across a range of pathologies, including oncology, neurodegenerative diseases, inflammatory conditions, and viral infections, and may represent a transformative approach in the pursuit of efficacious PDE1B/10A-directed therapeutics for schizophrenia (Gustafson et al., 2015; Chen et al., 2022; Hirai et al., 2022).

## 9 Conclusion

Over the past decade, considerable efforts have been devoted to the discovery of selective PDE inhibitors for the treatment of schizophrenia, with PDE1B and PDE10A emerging as promising targets due to their regulatory roles in dopaminergic and glutamatergic neurotransmission. However, the advancement of selective inhibitors remains highly challenging as the PDE family comprises 11 isoforms with over 90% sequence homology. This structural similarity poses a significant obstacle to achieving isoform-specific inhibition of PDE1B and PDE10A. Moreover, the successful development of inhibitors is intricately linked to the efficient synthetic strategies, highlighting the need for an integrated and rational drug discovery approach. This review provides a comprehensive analysis of structure–activity relationships (SARs), binding site interactions, and synthetic strategies for both individual and dual PDE1B/10A inhibitors. Crystallographic studies have unveiled key PDE1B residues contributing to selectivity and affinity, such as pyrazolo-pyrimidinone, thieno-triazolo-pyrimidinone, and pyrazolo-pyrimidone, which engage the “hydrophobic clamp” region formed by residues L388, F424, H373, F392, M389, F392, and V417. Additional water-mediated hydrogen bonding between PDE1B inhibitors (e.g., pyrazolo-pyrimidone and quinazoline cores) and residues such as Q421, Y222, and H373 further enhances binding affinity. Similarly, in PDE10A, chromone and fluorophenyl-pyrazole scaffolds have demonstrated effective hydrophobic interactions with residues F729, I692, F696, Y683, and F719. Hydrogen bonding with Y683 near the Q2 selectivity pocket confers isoform specificity. Several heteroaromatic cores, including imidazo-benzimidazole, pyrimido-indazoles, and pyrazolo-pyrimidine, have been identified as critical pharmacophores that contribute to interactions with Y683, thereby enabling the rational design of future inhibitors.

Despite these advances, the discovery of dual PDE1B/PDE10A inhibitors remains largely underexplored. Only two studies to date have reported dual inhibitory compounds, specifically compounds **84 (Zinc41306568)** and **87**–**90**. Among these, only Compound **84** has undergone *in vivo* evaluation in ketamine-induced rodent models of schizophrenia, offering preliminary validation of dual inhibition as a viable therapeutic approach that warrants further investigation. However, several translational barriers remain unaddressed. Dose optimization is complicated by the distinct tissue distribution and enzymatic kinetics of PDE1B and PDE10A, potentially impacting the therapeutic index and adverse effect profiles. Additional challenges include limited blood–brain barrier permeability, metabolic instability, and off-target interactions, all of which hinder clinical advancement. Emerging technologies, such as PROTACs, provide a promising avenue for selectively degrading PDE isoforms, thereby mitigating issues related to functional redundancy and PK (Gustafson et al., 2015; Chen et al., 2022; Hirai et al., 2022).

To date, no selective PDE1B, PDE10A, or dual inhibitors have received clinical approval. Nevertheless, the identification of dual PDE1B/PDE10A inhibitors such as compounds **84 (Zinc41306568)** and **87**–**90** underscores growing interest in leveraging synergistic modulation of dopaminergic pathways by enhancing activity in the direct pathway while attenuating the indirect pathway. Future directions should emphasize the development of targeted degradation strategies, robust *in vivo* pharmacodynamic evaluations, and early-phase toxicity assessments to bridge the gap between promising lead molecules and clinically effective therapies for schizophrenia.

## References

[B1] Abi-Dargham A. Gil R. Krystal J. Baldwin R. M. Seibyl J. P. Bowers M. (1998). Increased striatal dopamine transmission in schizophrenia: confirmation in a second cohort. Am. J. Psychiatry 155, 761–767. 10.1176/ajp.155.6.761 9619147

[B2] Al-Nema M. Y. Gaurav A. (2020). Phosphodiesterase as a target for cognition enhancement in schizophrenia. Curr. Top. Med. Chem. 20, 2404–2421. 10.2174/1568026620666200613202641 32533817

[B3] Al-Nema M. Gaurav A. Lee V. S. Gunasekaran B. Lee M. T. Okechukwu P. (2021). Identification of dual inhibitor of phosphodiesterase 1B/10A using structure-based drug design approach. J. Mol. Liq. 342, 117485. 10.1016/j.molliq.2021.117485

[B4] Al-Nema M. Gaurav A. Lee M. T. Okechukwu P. Nimmanpipug P. Lee V. S. (2022a). Evaluation of the acute oral toxicity and antipsychotic activity of a dual inhibitor of PDE1B and PDE10A in rat model of schizophrenia. PLoS One 17, e0278216. 10.1371/journal.pone.0278216 36454774 PMC9714703

[B5] Al-Nema M. Gaurav A. Lee V. S. Gunasekaran B. Lee M. T. Okechukwu P. (2022b). Structure-based discovery and bio-evaluation of a cyclopenta[4,5]thieno[2,3-: D] pyrimidin-4-one as a phosphodiesterase 10A inhibitor. RSC Adv. 12, 1576–1591. 10.1039/d1ra07649c PMC8979230 35425186

[B6] Al-Nema M. Gaurav A. Lee V. S. (2023). Designing of 2,3-dihydrobenzofuran derivatives as inhibitors of PDE1B using pharmacophore screening, ensemble docking and molecular dynamics approach. Comput. Biol. Med. 159, 106869. 10.1016/j.compbiomed.2023.106869 37071939

[B7] Amin H. S. Parikh P. K. Ghate M. D. (2021). Medicinal chemistry strategies for the development of phosphodiesterase 10A (PDE10A) inhibitors - an update of recent progress. Eur. J. Med. Chem. 214, 113155. 10.1016/j.ejmech.2021.113155 33581555

[B8] Arakawa K. Maehara S. Yuge N. Ishikawa M. Miyazaki Y. Naba H. (2016). Pharmacological characterization of a novel potent, selective, and orally active phosphodiesterase 10A inhibitor, PDM-042 [(E)-4-(2-(2-(5,8-dimethyl-[1,2,4]triazolo[1,5-a]pyrazin-2-yl)vinyl)-6-(pyrrolidin-1-yl)pyrimidin-4-yl)morpholine] in rats: potential for the treatment of schizophrenia. Pharmacol. Res. Perspect. 4, e00241. 10.1002/prp2.241 28116094 PMC5242175

[B9] Argyrousi E. K. Heckman P. R. A. Prickaerts J. (2020). Role of cyclic nucleotides and their downstream signaling cascades in memory function: being at the right time at the right spot. Neurosci. Biobehav. Rev. 113, 12–38. 10.1016/j.neubiorev.2020.02.004 32044374

[B10] Bartolomé-Nebreda J. M. Delgado F. Martín-Martín M. L. Martínez-Viturro C. M. Pastor J. Tong H. M. (2014). Discovery of a potent, selective, and orally active phosphodiesterase 10A inhibitor for the potential treatment of schizophrenia. J. Med. Chem. 57, 4196–4212. 10.1021/jm500073h 24758746

[B11] Beavo J. A. (1995). Cyclic nucleotide phosphodiesterases: functional implications of multiple isoforms. Physiol. Rev. 75, 725–748. 10.1152/physrev.1995.75.4.725 7480160

[B12] Bhardwaj V. K. Purohit R. (2021). Computer simulation to identify selective inhibitor for human phosphodiesterase10A. J. Mol. Liq. 328, 115419. 10.1016/j.molliq.2021.115419

[B13] Bondarev A. D. Attwood M. M. Jonsson J. Chubarev V. N. Tarasov V. V. Liu W. (2022). Recent developments of phosphodiesterase inhibitors: clinical trials, emerging indications and novel molecules. Front. Pharmacol. 13, 1057083. 10.3389/fphar.2022.1057083 36506513 PMC9731127

[B14] Calabresi P. Picconi B. Tozzi A. Ghiglieri V. Di Filippo M. (2014). Direct and indirect pathways of basal ganglia: a critical reappraisal. Nat. Neurosci. 17, 1022–1030. 10.1038/nn.3743 25065439

[B15] Cardinale A. Fusco F. R. (2018). Inhibition of phosphodiesterases as a strategy to achieve neuroprotection in Huntington’s disease. CNS Neurosci. Ther. 24, 319–328. 10.1111/cns.12834 29500937 PMC6489766

[B16] Cerveri G. Gesi C. Mencacci C. (2019). Pharmacological treatment of negative symptoms in schizophrenia: update and proposal of a clinical algorithm. Neuropsychiatr. Dis. Treat. 15, 1525–1535. 10.2147/NDT.S201726 31239687 PMC6556563

[B17] Chappie T. A. Helal C. J. Hou X. (2012). Current landscape of phosphodiesterase 10A (PDE10A) inhibition. J. Med. Chem. 55, 7299–7331. 10.1021/jm3004976 22834877

[B18] Chen S. Li X. Li Y. Yuan X. Geng C. Gao S. (2022). Design of stapled peptide-based PROTACs for MDM2/MDMX atypical degradation and tumor suppression. Theranostics 12, 6665–6681. 10.7150/thno.75444 PMC9516243 36185610

[B19] Cheung W. Y. (1970). Cyclic 3′,5′-nucleotide phosphodiesterase. Demonstration of an activator. Biochem. Biophys. Res. Commun. 38, 533–538. 10.1016/0006-291X(70)90747-3 4315350

[B20] Chino A. Seo R. Amano Y. Namatame I. Hamaguchi W. Honbou K. (2018). Fragment-based discovery of pyrimido[1,2-B]indazole PDE10A inhibitors. Chem. Pharm. Bull. 66, 286–294. 10.1248/cpb.c17-00836 29491261

[B21] Chino A. Honda S. Morita M. Yonezawa K. Hamaguchi W. Amano Y. (2019). Synthesis, SAR study, and biological evaluation of novel 2,3-dihydro-1H-imidazo[1,2-a]benzimidazole derivatives as phosphodiesterase 10A inhibitors. Bioorg. Med. Chem. 27, 3692–3706. 10.1016/j.bmc.2019.07.010 31301949

[B22] Conti M. Beavo J. (2007). Biochemistry and physiology of cyclic nucleotide phosphodiesterases: essential components in cyclic nucleotide signaling. Annu. Rev. Biochem. 76, 481–511. 10.1146/annurev.biochem.76.060305.150444 17376027

[B23] Daina A. Michielin O. Zoete V. (2017). SwissADME: a free web tool to evaluate pharmacokinetics, drug-likeness and medicinal chemistry friendliness of small molecules. Sci. Rep. 7, 42717. 10.1038/srep42717 28256516 PMC5335600

[B24] Demartinis N. Lopez R. N. Pickering E. H. Schmidt C. J. Gertsik L. Walling D. P. (2019). A proof-of-concept study evaluating the phosphodiesterase 10A inhibitor PF-02545920 in the adjunctive treatment of suboptimally controlled symptoms of schizophrenia. J. Clin. Psychopharmacol. 39, 318–328. 10.1097/JCP.0000000000001047 31205187

[B25] Dyck B. Branstetter B. Gharbaoui T. Hudson A. R. Breitenbucher J. G. Gomez L. (2017). Discovery of selective phosphodiesterase 1 inhibitors with memory enhancing properties. J. Med. Chem. 60, 3472–3483. 10.1021/acs.jmedchem.7b00302 28406621

[B26] Essayan D. M. (2001). Cyclic nucleotide phosphodiesterases. J. Allergy Clin. Immunol. 108, 671–680. 10.1067/mai.2001.119555 11692087

[B27] Francis S. H. Blount M. A. Corbin J. D. (2011). Mammalian cyclic nucleotide phosphodiesterases: molecular mechanisms and physiological functions. Physiol. Rev. 91, 651–690. 10.1152/physrev.00030.2010 21527734

[B28] Garcia A. M. Redondo M. Martinez A. Gil C. (2014). Phosphodiesterase 10 inhibitors: new disease modifying drugs for parkinson’s disease? Curr. Med. Chem. 21, 1171–1187. 10.2174/0929867321666131228221749 24372206

[B29] Gentzel R. C. Toolan D. Roberts R. Koser A. J. Kandebo M. Hershey J. (2015). The PDE10A inhibitor MP-10 and haloperidol produce distinct gene expression profiles in the striatum and influence cataleptic behavior in rodents. Neuropharmacology 99, 256–263. 10.1016/j.neuropharm.2015.05.024 26044638

[B30] Giembycz M. A. Maurice D. H. (2014). Cyclic nucleotide-based therapeutics for chronic obstructive pulmonary disease. Curr. Opin. Pharmacol. 16, 89–107. 10.1016/j.coph.2014.04.001 24810285

[B31] Goff D. C. Cather C. Freudenreich O. Henderson D. C. Evins A. E. Culhane M. A. (2009). A placebo-controlled study of sildenafil effects on cognition in schizophrenia. Psychopharmacol. Berl. 202, 411–417. 10.1007/s00213-008-1278-5 18716761 PMC2704618

[B32] Goonathilake M. R. Waqar S. George S. Jean-Baptiste W. Yusuf Ali A. Inyang B. (2022). Can phosphodiesterase 4 inhibitor therapy be used in respiratory diseases other than chronic obstructive pulmonary disease? Cureus 14, e27132. 10.7759/cureus.27132 36017299 PMC9392891

[B33] Goraya T. A. Cooper D. M. F. (2005). Ca2+-calmodulin-dependent phosphodiesterase (PDE1): current perspectives. Cell. Signal. 17, 789–797. 10.1016/j.cellsig.2004.12.017 15763421

[B34] Grauer S. M. Pulito V. L. Navarra R. L. Kelly M. P. Kelley C. Graf R. (2009). Phosphodiesterase 10A inhibitor activity in preclinical models of the positive, cognitive, and negative symptoms of schizophrenia. J. Pharmacol. Exp. Ther. 331, 574–590. 10.1124/jpet.109.155994 19661377

[B35] Groenewegen H. J. (2003). The basal ganglia and motor control. Neural Plast. 10, 107–120. 10.1155/NP.2003.107 14640312 PMC2565420

[B36] Gustafson J. L. Neklesa T. K. Cox C. S. Roth A. G. Buckley D. L. Tae H. S. (2015). Small-molecule-mediated degradation of the androgen receptor through hydrophobic tagging. Angew. Chem. - Int. Ed. 54, 9659–9662. 10.1002/anie.201503720 26083457 PMC4547777

[B37] Halpin D. M. G. (2008). ABCD of the phosphodiesterase family: interaction and differential activity in COPD. Int. J. COPD 3, 543–561. 10.2147/copd.s1761 19281073 PMC2650605

[B38] Hamzehnejadi M. Ranjbar Tavakoli M. Abiri A. Ghasempour A. Langarizadeh M. A. Forootanfar H. (2022). A review on Phosphodiesterase-5 inhibitors as a topical therapy for erectile dysfunction. Sex. Med. Rev. 10, 376–391. 10.1016/j.sxmr.2022.02.002 35370122

[B39] Hayes J. Laursen B. Eneberg E. Kehler J. Rasmussen L. K. Langgard M. (2021). Phosphodiesterase type 1 inhibition alters medial prefrontal cortical activity during goal-driven behaviour and partially reverses neurophysiological deficits in the rat phencyclidine model of schizophrenia. Neuropharmacology 186, 108454. 10.1016/j.neuropharm.2021.108454 33444639

[B40] Heckman P. R. A. Van Duinen M. A. Bollen E. P. P. Nishi A. Wennogle L. P. Blokland A. (2016). Phosphodiesterase inhibition and regulation of dopaminergic frontal and striatal functioning: clinical implications. Int. J. Neuropsychopharmacol. 19, pyw030. 10.1093/ijnp/pyw030 27037577 PMC5091819

[B41] Hegarty J. D. Baldessarini R. J. Tohen M. Waternaux C. Oepen G. (1994). One hundred years of schizophrenia: a meta-analysis of the outcome literature. Am. J. Psychiatry 151, 1409–1416. 10.1176/ajp.151.10.1409 8092334

[B42] Hietala J. Syvälahti E. Kuoppamäki M. Hietala J. Syvälahti E. Haaparanta M. (1995). Presynaptic dopamine function in striatum of neuroleptic-naive schizophrenic patients. Lancet 346, 1130–1131. 10.1016/S0140-6736(95)91801-9 7475604

[B43] Hietala J. Syvälahti E. Vilkman H. Vuorio K. Räkköläinen V. Bergman J. (1999). Depressive symptoms and presynaptic dopamine function in neuroleptic-naive schizophrenia. Schizophr. Res. 35, 41–50. 10.1016/S0920-9964(98)00113-3 9988840

[B44] Hikida T. Kimura K. Wada N. Funabiki K. Nakanishi Shigetada S. (2010). Distinct roles of synaptic transmission in direct and indirect striatal pathways to reward and aversive behavior. Neuron 66, 896–907. 10.1016/j.neuron.2010.05.011 20620875

[B45] Hindmarch I. Fuchs H. H. Erzigkeit H. (1991). Efficacy and tolerance of vinpocetine in ambulant patients suffering from mild to moderate organic psychosyndromes. Int. Clin. Psychopharmacol. 6, 31–43. 10.1097/00004850-199100610-00005 2071888

[B46] Hirai K. Yamashita H. Tomoshige S. Mishima Y. Niwa T. Ohgane K. (2022). Conversion of a PROTAC mutant huntingtin degrader into small-molecule hydrophobic tags focusing on drug-like properties. ACS Med. Chem. Lett. 13, 396–402. 10.1021/acsmedchemlett.1c00500 35300080 PMC8919385

[B47] Humphrey J. M. (2014). “Selective new small-molecule inhibitors of phosphodiesterase 1,” in Phosphodiesterases and their inhibitors. Hoboken, NJ: John Wiley & Sons. 10.1002/9783527682348.ch10

[B48] Insel T. R. (2010). Rethinking schizophrenia. Nature 468, 187–193. 10.1038/nature09552 21068826

[B49] Jancic D. Lopez De Armentia M. Valor L. M. Olivares R. Barco A. (2009). Inhibition of cAMP response element-binding protein reduces neuronal excitability and plasticity, and triggers neurodegeneration. Cereb. Cortex 19, 2535–2547. 10.1093/cercor/bhp004 19213815

[B50] Janjua S. Fortescue R. Poole P. (2020). Phosphodiesterase-4 inhibitors for chronic obstructive pulmonary disease. Cochrane Database Syst. Rev. 5, CD002309. 10.1002/14651858.CD002309.pub6 32356609 PMC7193764

[B51] Kakiuchi S. Yamazaki R. (1970). Calcium dependent phosphodiesterase activity and its activating factor (PAF) from brain. Studies on cyclic 3′,5′-nucleotide phosphodiesterase (III). Biochem. Biophys. Res. Commun. 41, 1104–1110. 10.1016/0006-291X(70)90199-3 4320714

[B52] Kamel R. Leroy J. Vandecasteele G. Fischmeister R. (2023). Cyclic nucleotide phosphodiesterases as therapeutic targets in cardiac hypertrophy and heart failure. Nat. Rev. Cardiol. 20, 90–108. 10.1038/s41569-022-00756-z 36050457

[B53] Kandel E. R. (2012). The molecular biology of memory: CAMP, PKA, CRE, CREB-1, CREB-2, and CPEB. Mol. Brain 5, 14. 10.1186/1756-6606-5-14 22583753 PMC3514210

[B54] Ke H. Wang H. (2007). Crystal structures of phosphodiesterases and implications on substrate specificity and inhibitor selectivity. Curr. Top. Med. Chem. 7, 391–403. 10.2174/156802607779941242 17305581

[B55] Ke H. Wang H. Ye M. (2011). Structural insight into the substrate specificity of phosphodiesterases. Handb. Exp. Pharmacol. 204, 121–134. 10.1007/978-3-642-17969-3_4 21695637

[B56] Kehler J. Nielsen J. (2011). PDE10A inhibitors: novel therapeutic drugs for schizophrenia. Curr. Pharm. Des. 17, 137–150. 10.2174/138161211795049624 21355834

[B57] Keravis T. Lugnier C. (2012). Cyclic nucleotide phosphodiesterase (PDE) isozymes as targets of the intracellular signalling network: benefits of PDE inhibitors in various diseases and perspectives for future therapeutic developments. Br. J. Pharmacol. 165, 1288–1305. 10.1111/j.1476-5381.2011.01729.x 22014080 PMC3372715

[B58] Kim J. Kwon J. T. Kim H. S. Han J. H. (2013). CREB and neuronal selection for memory trace. Front. Neural Circuits. 7, 44. 10.3389/fncir.2013.00044 23519079 PMC3604628

[B59] Kochoian B. A. Bure C. Papa S. M. (2023). Targeting striatal glutamate and phosphodiesterases to control L-DOPA-Induced dyskinesia. Cells 12, 2754. 10.3390/cells12232754 38067182 PMC10706484

[B60] Koizumi Y. Tanaka Y. Matsumura T. Kadoh Y. Miyoshi H. Hongu M. (2019). Discovery of a pyrazolo[1,5-a]pyrimidine derivative (MT-3014) as a highly selective PDE10A inhibitor *via* core structure transformation from the stilbene moiety. Bioorg. Med. Chem. 27, 3440–3450. 10.1016/j.bmc.2019.06.021 31235264

[B61] Krause M. Zhu Y. Huhn M. Schneider-Thoma J. Bighelli I. Nikolakopoulou A. (2018). Antipsychotic drugs for patients with schizophrenia and predominant or prominent negative symptoms: a systematic review and meta-analysis. Eur. Arch. Psychiatry Clin. Neurosci. 268, 625–639. 10.1007/s00406-018-0869-3 29368205

[B62] Kuzmiszyn A. K. Selli A. L. Smaglyukova N. Kondratiev T. Fuskevåg O. M. Lyså R. A. (2022). Treatment of cardiovascular dysfunction with PDE3-Inhibitors in moderate and severe hypothermia—effects on cellular elimination of cyclic adenosine monophosphate and cyclic guanosine monophosphate. Front. Physiol. 13, 923091. 10.3389/fphys.2022.923091 35910566 PMC9326216

[B63] Kwiatkowski M. Zhang J. Zhou W. Gehring C. Wong A. (2024). Cyclic nucleotides – the rise of a family. Trends Plant Sci. 29, 915–924. 10.1016/j.tplants.2024.02.003 38480090

[B64] Lakics V. Karran E. H. Boess F. G. (2010). Quantitative comparison of phosphodiesterase mRNA distribution in human brain and peripheral tissues. Neuropharmacology 59, 367–374. 10.1016/j.neuropharm.2010.05.004 20493887

[B65] Lane C. A. Hardy J. Schott J. M. (2018). Alzheimer’s disease. Eur. J. Neurol. 25, 59–70. 10.1111/ene.13439 28872215

[B66] Layton M. E. Kern J. C. Hartingh T. J. Shipe W. D. Raheem I. Kandebo M. (2023). Discovery of MK-8189, a highly potent and selective PDE10A inhibitor for the treatment of schizophrenia. J. Med. Chem. 66, 1157–1171. 10.1021/acs.jmedchem.2c01521 36624931 PMC9884086

[B67] Li P. Zheng H. Zhao J. Zhang L. Yao W. Zhu H. (2016). Discovery of potent and selective inhibitors of phosphodiesterase 1 for the treatment of cognitive impairment associated with neurodegenerative and neuropsychiatric diseases. J. Med. Chem. 59, 1149–1164. 10.1021/acs.jmedchem.5b01751 26789933

[B68] Li J. Song P. Wang H. Lian W. Li J. Wang Z. (2025). Selectivity mechanism of inhibition towards phosphodiesterase 1B and phosphodiesterase 10A *in silico* investigation. Comput. Biol. Chem. 115, 108322. 10.1016/j.compbiolchem.2024.108322 39778285

[B69] Lindholm E. Ekholm B. Shaw S. Jalonen P. Johansson G. Pettersson U. (2001). A schizophrenia-susceptibility locus at 6q25, in one of the world’s largest reported pedigrees. Am. J. Hum. Genet. 69, 96–105. 10.1086/321288 11389481 PMC1226052

[B70] Loughney K. Snyder P. B. Uher L. Rosman G. J. Ferguson K. Florio V. A. (1999). Isolation and characterization of PDE10A, a novel human 3ʹ, 5ʹ-cyclic nucleotide phosphodiesterase. Gene 234, 109–117. 10.1016/S0378-1119(99)00171-7 10393245

[B71] Luo S. X. Huang E. J. (2016). Dopaminergic neurons and brain reward pathways: from neurogenesis to circuit assembly. Am. J. Pathol. 186, 478–488. 10.1016/j.ajpath.2015.09.023 26724386 PMC4816693

[B72] Lusardi M. Rapetti F. Spallarossa A. Brullo C. (2024). PDE4D: a multipurpose pharmacological target. Int. J. Mol. Sci. 25, 8052. 10.3390/ijms25158052 39125619 PMC11311937

[B73] Macek T. A. McCue M. Dong X. Hanson E. Goldsmith P. Affinito J. (2019). A phase 2, randomized, placebo-controlled study of the efficacy and safety of TAK-063 in subjects with an acute exacerbation of schizophrenia. Schizophr. Res. 204, 289–294. 10.1016/j.schres.2018.08.028 30190165

[B74] MacKenzie S. J. Baillie G. S. McPhee I. Bolger G. B. Houslay M. D. (2000). ERK2 mitogen-activated protein kinase binding, phosphorylation, and regulation of the PDE4D cAMP-specific phosphodiesterases. The involvement of COOH-terminal docking sites and NH2-terminal UCR regions. J. Biol. Chem. 275, 16609–16617. 10.1074/jbc.275.22.16609 10828059

[B75] Maurice D. H. Ke H. Ahmad F. Wang Y. Chung J. Manganiello V. C. (2014). Advances in targeting cyclic nucleotide phosphodiesterases. Nat. Rev. Drug Discov. 13, 290–314. 10.1038/nrd4228 24687066 PMC4155750

[B76] Mayr B. Montminy M. (2001). Transcriptional regulation by the phosphorylation-dependent factor creb. Nat. Rev. Mol. Cell Biol. 2, 599–609. 10.1038/35085068 11483993

[B77] McCleery A. Nuechterlein K. H. (2019). Cognitive impairment in psychotic illness: prevalence, profile of impairment, developmental course, and treatment considerations. Dialogues Clin. Neurosci. 21, 239–248. 10.31887/DCNS.2019.21.3/amccleery PMC6829172 31749648

[B78] McCutcheon R. A. Keefe R. S. E. McGuire P. K. (2023). Cognitive impairment in schizophrenia: aetiology, pathophysiology, and treatment. Mol. Psychiatry. 28, 1902–1918. 10.1038/s41380-023-01949-9 36690793 PMC10575791

[B79] McGrath J. Saha S. Welham J. El Saadi O. MacCauley C. Chant D. (2004). A systematic review of the incidence of schizophrenia: the distribution of rates and the influence of sex, urbanicity, migrant status and methodology. BMC Med. 2, 13. 10.1186/1741-7015-2-13 15115547 PMC421751

[B80] McQuown S. Xia S. Baumgärtel K. Barido R. Anderson G. Dyck B. (2019). Phosphodiesterase 1b (PDE1B) regulates spatial and contextual memory in hippocampus. Front. Mol. Neurosci. 12, 21. 10.3389/fnmol.2019.00021 30792627 PMC6374598

[B81] Merz K. Herold S. Lie D. C. (2011). CREB in adult neurogenesis - master and partner in the development of adult-born neurons? Eur. J. Neurosci. 33, 1078–1086. 10.1111/j.1460-9568.2011.07606.x 21395851

[B82] Meyer-Lindenberg A. Nielsen J. Such P. Lemming O. M. Zambori J. Buller R. (2022). A double-blind, randomized, placebo-controlled proof of concept study of the efficacy and safety of Lu AF11167 for persistent negative symptoms in people with schizophrenia. Eur. Neuropsychopharmacol. 61, 4–14. 10.1016/j.euroneuro.2022.05.009 35704951

[B83] Millar J. K. Pickard B. S. Mackie S. James R. Christie S. Buchanan S. R. (2005). Genetics: DISC1 and PDE4B are interacting genetic factors in schizoprenia that regulate cAMP signaling. Sci. (80) 310, 1187–1191. 10.1126/science.1112915 16293762

[B84] Mokra D. Mokry J. (2021). Phosphodiesterase inhibitors in acute lung injury: what are the perspectives? Int. J. Mol. Sci. 22, 1929. 10.3390/ijms22041929 33669167 PMC7919656

[B85] Morimoto B. H. Koshland D. E. (1991). Identification of cyclic AMP as the response regulator for neurosecretory potentiation: a memory model system. Proc. Natl. Acad. Sci. U. S. A. 88, 10835–10839. 10.1073/pnas.88.23.10835 1660152 PMC53026

[B86] Mukai Y. Lupinacci R. Marder S. Snow-adami L. Voss T. Smith S. M. (2024). Effects of PDE10A inhibitor MK-8189 in people with an acute episode of schizophrenia: a randomized proof-of-concept clinical trial. Schizophr. Res. 270, 37–43. 10.1016/j.schres.2024.05.019 38851166

[B87] Nadur N. F. de Azevedo L. L. Caruso L. Graebin C. S. Lacerda R. B. Kümmerle A. E. (2021). The long and winding road of designing phosphodiesterase inhibitors for the treatment of heart failure. Eur. J. Med. Chem. 212, 113123. 10.1016/j.ejmech.2020.113123 33412421

[B88] Neef J. Palacios D. S. (2021). Progress in mechanistically novel treatments for schizophrenia. RSC Med. Chem. 12, 1459–1475. 10.1039/d1md00096a PMC8459322 34671731

[B89] Nishi A. Snyder G. L. (2010). Advanced research on dopamine signaling to develop drugs for the treatment of mental disorders: biochemical and behavioral profiles of phosphodiesterase inhibition in dopaminergic neurotransmission. J. Pharmacol. Sci. 114, 6–16. 10.1254/jphs.10R01FM 20716858

[B90] Nishi A. Kuroiwa M. Miller D. B. O’Callaghan J. P. Bateup H. S. Shuto T. (2008). Distinct roles of PDE4 and PDE10A in the regulation of cAMP/PKA signaling in the striatum. J. Neurosci. 28, 10460–10471. 10.1523/JNEUROSCI.2518-08.2008 18923023 PMC2814340

[B91] Pekcec A. Schülert N. Stierstorfer B. Deiana S. Dorner-Ciossek C. Rosenbrock H. (2018). Targeting the dopamine D1 receptor or its downstream signalling by inhibiting phosphodiesterase-1 improves cognitive performance. Br. J. Pharmacol. 175, 3021–3033. 10.1111/bph.14350 29726015 PMC6016630

[B92] Pickard B. S. Thomson P. A. Christoforou A. Evans K. L. Morris S. W. Porteous D. J. (2007). The PDE4B gene confers sex-specific protection against schizophrenia. Psychiatr. Genet. 17, 129–133. 10.1097/YPG.0b013e328014492b 17417055

[B93] Prickaerts J. Heckman P. R. A. Blokland A. (2017). Investigational phosphodiesterase inhibitors in phase I and phase II clinical trials for Alzheimer’s disease. Expert Opin. Investig. Drugs 26, 1033–1048. 10.1080/13543784.2017.1364360 28772081

[B94] Rajput P. S. Kharmate G. Somvanshi R. K. Kumar U. (2009). Colocalization of dopamine receptor subtypes with dopamine and cAMP-regulated phosphoprotein (DARPP-32) in rat brain. Neurosci. Res. 65, 53–63. 10.1016/j.neures.2009.05.005 19465068

[B95] Reed T. M. Repaske D. R. Snyder G. L. Greengard P. Vorhees C. V. (2002). Phosphodiesterase 1B knock-out mice exhibit exaggerated locomotor hyperactivity and DARPP-32 phosphorylation in response to dopamine agonists and display impaired spatial learning. J. Neurosci. 22, 5188–5197. 10.1523/jneurosci.22-12-05188.2002 12077213 PMC6757711

[B96] Remington G. Foussias G. Fervaha G. Agid O. Takeuchi H. Lee J. (2016). Treating negative symptoms in schizophrenia: an update. Curr. Treat. Options Psychiatry 3, 133–150. 10.1007/s40501-016-0075-8 27376016 PMC4908169

[B97] Reneerkens O. A. H. Rutten K. Bollen E. Hage T. Blokland A. Steinbusch H. W. M. (2013). Inhibition of phoshodiesterase type 2 or type 10 reverses object memory deficits induced by scopolamine or MK-801. Behav. Brain Res. 236, 16–22. 10.1016/j.bbr.2012.08.019 22951181

[B98] Rhee C. K. Kim D. K. (2020). Role of phosphodiesterase-4 inhibitors in chronic obstructive pulmonary disease. Korean J. Intern. Med. 35, 276–283. 10.3904/kjim.2020.035 32131571 PMC7061018

[B99] Ricciarelli R. Fedele E. (2018). cAMP, cGMP and amyloid β: three ideal partners for memory formation. Trends Neurosci. 41, 255–266. 10.1016/j.tins.2018.02.001 29501262

[B100] Rombaut B. Kessels S. Schepers M. Tiane A. Paes D. Solomina Y. (2021). PDE inhibition in distinct cell types to reclaim the balance of synaptic plasticity. Theranostics 11, 2080–2097. 10.7150/thno.50701 33500712 PMC7797685

[B101] Russwurm C. Koesling D. Russwurm M. (2015). Phosphodiesterase 10A is tethered to a synaptic signaling complex in striatum. J. Biol. Chem. 290, 11936–11947. 10.1074/jbc.M114.595769 25762721 PMC4424333

[B102] Sadeghi M. A. Nassireslami E. Yousefi Zoshk M. Hosseini Y. Abbasian K. Chamanara M. (2023). Phosphodiesterase inhibitors in psychiatric disorders. Psychopharmacol. Berl. 240, 1201–1219. 10.1007/s00213-023-06361-3 37060470

[B103] Schröder S. Scheunemann M. Wenzel B. Brust P. (2021). Challenges on cyclic nucleotide phosphodiesterases imaging with positron emission tomography: novel radioligands and (pre-)clinical insights since 2016. Int. J. Mol. Sci. 22, 3832. 10.3390/ijms22083832 33917199 PMC8068090

[B104] Sharma B. Purohit R. (2023). Structural insights into the lead identification of sub-type selective PDE4B inhibitors from plant bioactive molecule analogues. J. Mol. Liq. 390, 123039. 10.1016/j.molliq.2023.123039

[B105] Sharma B. Bhattacherjee D. Zyryanov G. V. Purohit R. (2023). An insight from computational approach to explore novel, high-affinity phosphodiesterase 10A inhibitors for neurological disorders. J. Biomol. Struct. Dyn. 41, 9424–9436. 10.1080/07391102.2022.2141895 36336960

[B106] Sheng J. Zhang S. Wu L. Kumar G. Liao Y. Gk P. (2022). Inhibition of phosphodiesterase: a novel therapeutic target for the treatment of mild cognitive impairment and Alzheimer’s disease. Front. Aging Neurosci. 14, 1019187. 10.3389/fnagi.2022.1019187 36268188 PMC9577554

[B107] Shi M. Ding J. Li L. Bai H. Li X. Lan L. (2021). Effects of ketamine on learning and memory in the hippocampus of rats through erk, creb, and arc. Brain Sci. 11, 27. 10.3390/brainsci11010027 PMC7824469 33383707

[B108] Shirayama Y. Konishi T. Hashimoto K. (2011). Effects of add-on cilostazol on cognition in patients with schizophrenia: an open-label pilot trial. J. Clin. Psychopharmacol. 31, 659–661. 10.1097/JCP.0b013e31822c94fd 21881450

[B109] Silva A. J. Kogan J. H. Frankland P. W. Kida S. (1998). CREB and memory. Annu. Rev. Neurosci. 21, 127–148. 10.1146/annurev.neuro.21.1.127 9530494

[B110] Soderling S. H. Bayuga S. J. Beavo J. A. (1999). Isolation and characterization of a dual-substrate phosphodiesterase gene family: PDE10A. Proc. Natl. Acad. Sci. U. S. A. 96, 7071–7076. 10.1073/pnas.96.12.7071 10359840 PMC22059

[B111] Solmi M. Murru A. Pacchiarotti I. Undurraga J. Veronese N. Fornaro M. (2017). Safety, tolerability, and risks associated with first-and second-generation antipsychotics: a state-of-the-art clinical review. Ther. Clin. Risk Manag. 13, 757–777. 10.2147/TCRM.S117321 28721057 PMC5499790

[B112] Soon C. W. Gaurav A. Gautam V. Al-Nema M. (2024). Structural insight into the lead identification of a dual inhibitor of PDE1B and PDE10A: integrating pharmacophore-based virtual screening, molecular docking, and structure-activity-relationship approaches. Heliyon 10, e38305. 10.1016/j.heliyon.2024.e38305 39391487 PMC11466560

[B113] Sun J. Xiao Z. Haider A. Gebhard C. Xu H. Luo H. B. (2021). Advances in cyclic nucleotide phosphodiesterase-targeted PET imaging and drug discovery. J. Med. Chem. 64, 7083–7109. 10.1021/acs.jmedchem.1c00115 34042442

[B114] Suzuki K. Kimura H. (2018). TAK-063, a novel PDE10A inhibitor with balanced activation of direct and indirect pathways, provides a unique opportunity for the treatment of schizophrenia. CNS Neurosci. Ther. 24, 604–614. 10.1111/cns.12798 29318783 PMC6489916

[B115] Svenningsson P. Nishi A. Fisone G. Girault J. A. Nairn A. C. Greengard P. (2004). DARPP-32: an integrator of neurotransmission. Annu. Rev. Pharmacol. Toxicol. 44, 269–296. 10.1146/annurev.pharmtox.44.101802.121415 14744247

[B116] Świerczek A. Jankowska A. Chłoń-Rzepa G. Pawłowski M. Wyska E. (2019). Advances in the discovery of PDE10A inhibitors for CNS-related disorders. Part 2: focus on schizophrenia. Curr. Drug Targets 20, 1652–1669. 10.2174/1389450120666190801114210 31368871

[B117] Threlfell S. West A. R. (2013). Modulation of striatal neuron activity by cyclic nucleotide signalling and phosphodiesterase inhibition. Basal Ganglia 3, 137–146. 10.1016/j.baga.2013.08.001 24490129 PMC3904398

[B118] Tsai M. Chrones L. Xie J. Gevorkyan H. Macek T. A. (2016). A phase 1 study of the safety, tolerability, pharmacokinetics, and pharmacodynamics of TAK-063, a selective PDE10A inhibitor. Psychopharmacol. Berl. 233, 3787–3795. 10.1007/s00213-016-4412-9 27572830 PMC5063900

[B119] Tully T. Bourtchouladze R. Scott R. Tallman J. (2003). Targeting the creb pathway for memory enhancers. Nat. Rev. Drug Discov. 2, 267–277. 10.1038/nrd1061 12669026

[B120] Ukena D. Schudt C. Sybrecht G. W. (1993). Adenosine receptor-blocking xanthines as inhibitors of phosphodiesterase isozymes. Biochem. Pharmacol. 45, 847–851. 10.1016/0006-2952(93)90168-V 7680859

[B121] Umbricht D. Abt M. Tamburri P. Chatham C. Holiga Š. Frank M. J. (2021). Proof-of-Mechanism study of the phosphodiesterase 10 inhibitor RG7203 in patients with schizophrenia and negative symptoms. Biol. Psychiatry Glob. Open Sci. 1, 70–77. 10.1016/j.bpsgos.2021.03.001 36324430 PMC9616307

[B122] Vemulapalli S. Watkins R. W. Chintala M. Davis H. Ahn H. S. Fawzi A. (1996). Antiplatelet and antiproliferative effects of SCH 51866, a novel type 1 and type 5 phosphodiesterase inhibitor. J. Cardiovasc. Pharmacol. 28, 862–869. 10.1097/00005344-199612000-00018 8961086

[B123] Wallis R. M. (1999). The pharmacology of sildenafil, a novel and selective inhibitor of phosphodiesterase (PDE) type 5. Folia Pharmacol. Jpn. 114, 22P-26P. 10.1254/fpj.114.supplement_22 10629850

[B124] Wang H. Liu Y. Hou J. Zheng M. Robinson H. Ke H. (2007). Structural insight into substrate specificity of phosphodiesterase 10. Proc. Natl. Acad. Sci. U. S. A. 104, 5782–5787. 10.1073/pnas.0700279104 17389385 PMC1851569

[B125] Winterer G. (2006). Cortical microcircuits in schizophrenia - the dopamine hypothesis revisited. Pharmacopsychiatry 39 Suppl 1, S68–S71. 10.1055/s-2006-931498 16508900

[B126] Xiao Z. Wei H. Xu Y. Haider A. Wei J. Yuan S. (2022). Discovery of a highly specific 18F-labeled PET ligand for phosphodiesterase 10A enabled by novel spirocyclic iodonium ylide radiofluorination. Acta Pharm. Sin. B 12, 1963–1975. 10.1016/j.apsb.2021.11.014 35847497 PMC9279629

[B127] Xie Z. Adamowicz W. O. Eldred W. D. Jakowski A. B. Kleiman R. J. Morton D. G. (2006). Cellular and subcellular localization of PDE10A, a striatum-enriched phosphodiesterase. Neuroscience 139, 597–607. 10.1016/j.neuroscience.2005.12.042 16483723 PMC1464838

[B128] Yu Y. F. Zhang C. Huang Y. Y. Zhang S. Zhou Q. Li X. (2020). Discovery and optimization of chromone derivatives as novel selective phosphodiesterase 10 inhibitors. ACS Chem. Neurosci. 11, 1058–1071. 10.1021/acschemneuro.0c00024 32105440

[B129] Zagórska A. (2020). Phosphodiesterase 10 (PDE10) inhibitors: an updated patent review (2014-present). Expert Opin. Ther. Pat. 30, 147–157. 10.1080/13543776.2020.1709444 31874060

[B130] Zagorska A. Partyka A. Bucki A. Gawalskax A. Czopek A. Pawlowski M. (2018). Phosphodiesterase 10 inhibitors - novel perspectives for psychiatric and neurodegenerative drug discovery. Curr. Med. Chem. 25, 3455–3481. 10.2174/0929867325666180309110629 29521210

[B131] Zhang K. Y. J. Card G. L. Suzuki Y. Artis D. R. Fong D. Gillette S. (2004). A glutamine switch mechanism for nucleotide selectivity by phosphodiesterases. Mol. Cell. 15, 279–286. 10.1016/j.molcel.2004.08.015 15260978

[B132] Zhang L. Chen L. Beck E. M. Chappie T. A. Coelho R. V. Doran S. D. (2017). The discovery of a novel phosphodiesterase (PDE) 4B-Preferring radioligand for positron emission tomography (PET) imaging. J. Med. Chem. 60, 8538–8551. 10.1021/acs.jmedchem.7b01050 28957634

[B133] Zhang B. Huang Y. Zhang S. R. Huang M. X. Zhang C. Luo H. B. (2021). Design, synthesis and biological evaluation of novel pyrazolopyrimidone derivatives as potent PDE1 inhibitors. Bioorg. Chem. 114, 105104. 10.1016/j.bioorg.2021.105104 34186466

[B134] Zorn A. Baillie G. (2023). Phosphodiesterase 7 as a therapeutic target – where are we now? Cell. Signal. 108, 110689. 10.1016/j.cellsig.2023.110689 37120115

